# Diagnostic and Clinical Value of Targeted Next‐Generation Sequencing for Pediatric Respiratory Infections in Northern China

**DOI:** 10.1111/crj.70185

**Published:** 2026-04-12

**Authors:** Lixiang Wang, Xue Yang, Lexia Yang, Ke Yin, Hui Zhong

**Affiliations:** ^1^ Department of Paediatrics Qingdao Huangdao District People's Hospital Qingdao Shandong China

**Keywords:** diagnostics, outcomes, pediatric respiratory tract infections, tNGS

## Abstract

**Background:**

Pediatric patients are particularly susceptible to respiratory tract infections (RTIs) due to ongoing maturation of pulmonary and immune function, highlighting the need for rapid and accurate pathogen identification. Although targeted next‐generation sequencing (tNGS) is increasingly applied in infectious disease diagnostics, its real‐world clinical utility in pediatric RTIs remains underexplored.

**Methods:**

We conducted a retrospective study of 940 hospitalized children with RTIs in northern China between April and December 2023. All patients underwent tNGS alongside conventional microbiological tests (CMTs), including PCR, culture, and serology. Diagnostic performance metrics—including sensitivity, specificity, positive predictive value (PPV), and negative predictive value (NPV)—were calculated. The clinical impact of tNGS was assessed by examining treatment adjustments, turnaround time (TAT), and patient outcomes.

**Results:**

tNGS demonstrated superior diagnostic performance compared with CMTs: sensitivity 91.38% versus 29.68%, specificity 91.03% versus 90.17%, PPV 97.68% versus 77.78%, and NPV 73.39% versus 54.60%. tNGS identified a broader spectrum of pathogens, including RNA viruses and low‐abundance organisms frequently missed by CMTs, and detected polymicrobial infections in 17.77% of cases versus 1.17% by CMTs. Based on tNGS, treatment was escalated in 35.32% and de‐escalated in 29.04% of patients, with over 90% of adjustments made within 48 h, facilitated by a mean TAT of 28.5 h. Clinical improvement was observed in most adjusted cases. Pathogen distribution showed age‐ and season‐specific patterns, underscoring the need for context‐informed diagnostics and therapy.

**Conclusion:**

tNGS enhances pathogen detection accuracy in pediatric RTIs, enables timely and appropriate treatment modifications, and supports antimicrobial stewardship. Its high sensitivity, rapid TAT, and capacity to identify co‐infections reinforce its clinical utility in guiding optimized management of pediatric respiratory infections.

## Graphical abstract

A total of 1209 patients with suspected respiratory tract infections (RTIs) who underwent targeted next‐generation sequencing (tNGS) were screened at Qingdao Huangdao District People's Hospital, Qingdao, China, from April 1, 2023, to December 31, 2023. Of these, 940 patients were enrolled in the study. The clinical outcomes of tNGS were evaluated and compared with conventional microbiological tests (CMTs) and computed tomography (CT) scans.

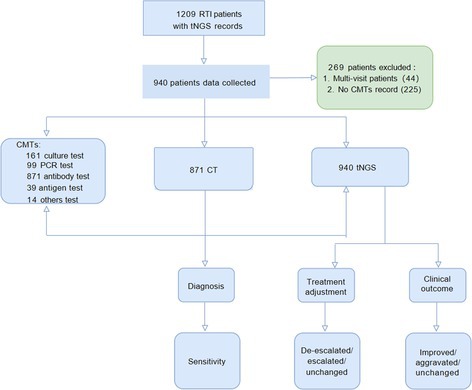

## 1 Introduction

Respiratory tract infections (RTIs) in children present critical clinical challenges due to their immature pulmonary function and relatively weaker immune systems [1, 2]. Compared with adults, pediatric patients with RTIs face a higher risk of severe complications and long‐term sequelae. Epidemiological studies report that 7%–13% of pediatric pneumonia cases progress to severe pneumonia, imposing a substantial burden on families and the healthcare system [3, 4, 5]. Moreover, frequent exposure to pathogens in communal settings—such as schools and daycare centers—further complicates infection control, contributing to the high prevalence and recurrent nature of RTIs in this population [6]. Thus, rapid and accurate pathogen identification, coupled with effective epidemiological surveillance, is essential for optimizing clinical management in children.

Conventional microbiological tests (CMTs)—including microbial culture, antigen–antibody assays, and PCR—are well established and widely used for diagnosing pediatric RTIs. However, these methods have notable limitations [7, 8]. Microbial cultures can only detect a limited spectrum of cultivable pathogens [9]; antigen tests often suffer from low sensitivity [10]; and PCR‐based assays require prior knowledge of the suspected pathogen, which limits their ability to detect atypical or unexpected agents and may result in high false‐negative rates. To address these issues, metagenomic next‐generation sequencing (mNGS) has emerged as an unbiased, high‐throughput approach capable of detecting a wide range of pathogens in a single test. Studies have shown that mNGS offers superior sensitivity over CMTs, especially for identifying rare or uncommon pathogens in pediatric RTIs [11, 12, 13, 14]. Nevertheless, mNGS also has drawbacks, including variable sensitivity depending on specimen type and disease severity, high costs, and difficulty in distinguishing true pathogens from environmental or commensal microorganisms due to its untargeted nature [15, 16, 17].

To mitigate these limitations, target enrichment–based next‐generation sequencing (tNGS) has been developed, which uses probe capture to selectively enrich genomic regions of interest. This approach preserves the broad detection capability of mNGS while increasing the proportion of target pathogen reads, improving turnaround time (TAT), and reducing costs. tNGS has exhibited promising performance in detecting pathogens across diverse infection sites—such as blood, joints, and the respiratory tract—and demonstrates enhanced sensitivity for low‐abundance pathogens compared to both mNGS and CMTs [18, 19]. For instance, tNGS has shown higher accuracy in identifying *Mycobacterium tuberculosis* and nontuberculous mycobacteria in pulmonary infections, underscoring its potential utility in respiratory diagnostics [20]. Additionally, tNGS can identify drug resistance–associated genes, aiding in personalized treatment decisions [21]. However, similar to mNGS, the diagnostic sensitivity of tNGS may be influenced by specimen type and disease severity. Therefore, further standardization of laboratory protocols and interpretation guidelines is needed before its widespread adoption in pediatric RTI management.

Most existing studies on tNGS in pediatric respiratory infections have primarily focused on bronchoalveolar lavage fluid, with limited research on other sample types and small‐scale patient cohorts [22, 23, 24]. The objective of this study is to evaluate the application of tNGS in the diagnosis of pediatric respiratory infections, with a particular focus on its potential in pathogen identification. Additionally, the study explores the clinical benefits of combining tNGS with traditional CMT in guiding treatment plan adjustments and assessing its impact on clinical decision‐making.

## 2 Methods

### 2.1 Patient Enrolment and Data Collection

**FIGURE 1:**
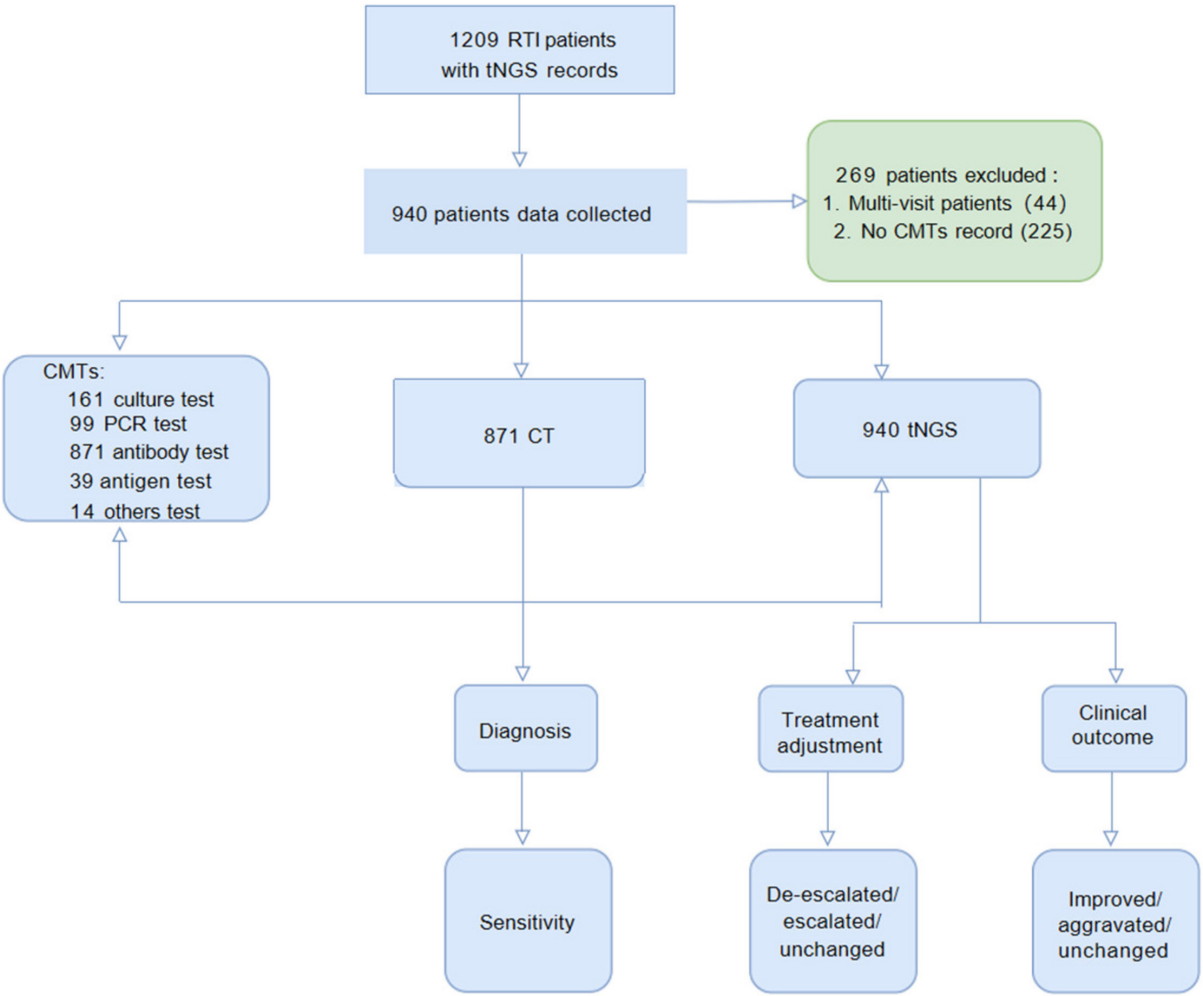
Flowchart of patient recruitment and sample collection. A total of 1209 patients with suspected RTIs who underwent tNGS were screened at Qingdao Huangdao District People's Hospital, Qingdao, China, from April 1, 2023, to December 31, 2023. Of these, 940 patients were enrolled in the study. The clinical outcomes of tNGS were evaluated and compared with CMTs and computed tomography (CT) scans. CT, computed tomography; RTI, respiratory tract infection; tNGS, targeted next‐generation sequencing.

This retrospective study was conducted at Qingdao Huangdao District People's Hospital in northern China. Children hospitalized in the pediatric department between April 1, 2023, and December 31, 2023, with RTIs who underwent tNGS testing were screened for eligibility (Figure 1). Demographic and clinical characteristics, including age, sex, and length of hospitalization, were extracted from electronic medical records. Additional data collected during hospitalization included complete blood count parameters, conventional pathogen test results, tNGS reports, imaging findings, and treatment details.

The diagnosis of RTI was established through comprehensive evaluation of clinical manifestations, epidemiological history, and pathogenic or serological evidence. Inclusion criteria required the presence of local respiratory symptoms (e.g., nasal congestion, sneezing, rhinorrhea, throat discomfort, sore throat, cough, sputum production, shortness of breath, wheezing, or hoarseness) and/or systemic manifestations (e.g., fever, fatigue, headache, restlessness, malaise, lethargy, decreased appetite, vomiting, diarrhea, or abdominal pain). Physical examination findings such as pharyngeal congestion, tonsillar enlargement, cervical lymphadenopathy, tachypnea, cyanosis, and pulmonary rales were also documented. Furthermore, patients were required to have at least one positive pathogenic or serological test result consistent with their clinical presentation, including positive PCR, antigen, or culture tests, detection of specific IgM during the convalescent phase, or a ≥ 4‐fold rise in IgG antibody titers between acute and convalescent phases.

Patients were excluded if they had multiple hospital visits or had incomplete medical records. The study adhered to the Declaration of Helsinki, and the protocol was approved by the Ethics Committee of Qingdao Huangdao District People's Hospital (LL‐LW‐2024001).

### 2.2 Sample Processing and CMTs

CMTs in this study included microbial culture, antigen and antibody tests, PCR assays, *Cryptococcus neoformans* ink stain microscopy, and (1,3)‐β‐D‐glucan (BDG) testing (Table S1). Microbial cultures were performed on urine, blood, or sputum samples. Urine and sputum samples were inoculated onto appropriate culture media and incubated at 37°C for 24–48 h. Blood cultures were inoculated into both aerobic and anaerobic culture bottles and processed in an automated blood culture system (e.g., BACTEC and BacT/Alert) for up to 5 days. Bacterial identification was carried out using the VITEK 2 Compact system (BioMérieux, France) according to the manufacturer's instructions.

Suspected pathogen antigens and antibodies were detected using immunofluorescence assays (IFA) targeting seven pathogens: rotavirus, influenza A, influenza B, adenovirus, *M. tuberculosis* , *Mycoplasma pneumoniae* , and Epstein–Barr virus. PCR tests targeted 10 pathogens: *M. tuberculosis* , *M. pneumoniae* , influenza A, influenza B, Epstein–Barr virus, cytomegalovirus, respiratory syncytial virus, rhinovirus, and adenovirus. DNA extraction for PCR tests was performed using reagents from Sansure Biotech Inc., following the manufacturer's instructions. *C. neoformans* ink stain microscopy was conducted on fresh cerebrospinal fluid (CSF) samples, which were centrifuged at 3000 rpm for 15 min, and the sediment was used for smears. BDG testing was performed using a commercial kit (Dynamiker Biotechnology (Tianjin) Co. Ltd.) for fungal cell wall detection, as per the manufacturer's instructions.

### 2.3 Nucleic Acid Extraction, Library Construction, and Sequencing

Throat swabs were collected from patients in tubes containing 1.5 mL of preservation liquid and thoroughly vortexed for 3 min. Subsequently, 1.3 mL of the homogenized sample was transferred to a new 1.5‐mL centrifuge tube, and 13 μL of external and internal reference was added. The sample was centrifuged at 12 000 rpm for 5 min to enrich the pathogen nucleic acids. After centrifugation, 700 μL of the supernatant was discarded, and the remaining sample was mixed thoroughly. A 250 μL aliquot was processed using the MasterPure DNA&RNA Extraction Kit (KS118‐BYTQ‐24, KingCreate Biotech) for nucleic acid extraction and purification.

Complementary DNA (cDNA) synthesis from RNA and targeted library construction were performed using the KM 50TM Plus Assay Kit (KingCreate Biotech), which included fragmentation, end repair, target region enrichment, adapter ligation, and library amplification. The enrichment panel targeted 107 common respiratory pathogens, including viruses (31 DNA viruses and 38 RNA viruses), bacteria (10 Gram‐positive bacteria and 12 Gram‐negative bacteria), mycoplasma (1), and chlamydia (4) (Table S2) [25] The targeted pathogens had at least one complete genome sequence available and had been previously reported in research studies. Probes were designed to target conserved regions such as ribosomal RNA genes (16S rRNA, 18S rRNA, or internal transcribed spacer) and housekeeping genes, enabling pathogen identification at the genus or species level. All libraries were sequenced on the KM MiniSeqDx‐CN platform (KingCreate Biotech) using a paired‐end model, with an average of 1 million reads per sample. Sequencing was performed by KingMed Diagnostics (Hong Kong) Limited.

The overall TAT for tNGS in this study averaged approximately 28.5 h from sample receipt to result reporting. To improve methodological transparency, the workflow can be divided into five major stages: (1) sample pretreatment and nucleic acid extraction (~2 h), (2) reverse transcription and library construction (~8 h), (3) hybrid capture and probe enrichment (~10 h), (4) sequencing on the MiniSeqDx‐CN platform (~6 h), and (5) bioinformatic processing and data interpretation (~2.5 h). These durations represent the average processing time under standard laboratory conditions at KingMed Diagnostics. The workflow was optimized for pediatric respiratory samples to achieve a balance between speed, sensitivity, and data quality, ensuring that clinically actionable results could be delivered within 30 h of specimen collection.

### 2.4 Bioinformatic Analysis

To obtain high‐quality reads, sequences containing adapters, low‐quality bases, excessive N bases, and those shorter than 35 bp were filtered using fastp (Version 0.20.1) [26]. Samples were considered valid for analysis if Q30 was ≥ 75%, the number of clean reads was ≥ 50 000, and the external/internal reference read numbers exceeded 200. Clean reads were aligned to the human reference genome (hg38) using Bowtie2 (Version 2.4.1) with the parameter “very‐sensitive” to remove human‐derived reads [27]. The remaining reads were then aligned to a classification reference genome database, which included all targeted pathogens and related species. This database also included species from the same genus and those with a genome average nucleotide identity greater than 80%. Genome sequences were obtained from the National Centre for Biotechnology Information (NCBI) genome database (ftp://ftp.ncbi.nlm.nih.gov/genomes/). The number of reads per 100 000 sequencing reads (RPhK) at the species and genus levels was calculated (Table S3). Bioinformatic analysis was performed by KingMed Diagnostics (Hong Kong) Limited.

### 2.5 Criteria for Diagnosis Decisions

Species in the database were classified into four categories: Category A (pathogenic species), Category B (opportunistic pathogens), Category C (normal respiratory microbiota), and Category D (others). For Category A pathogens, detection of any sequencing reads was considered positive; for Category B pathogens, a read count per 100 000 sequencing reads (RPhK) exceeding 2000 was required; and for Category C organisms, an RPhK value greater than 3500 was used as the positivity threshold. For Category D organisms, additional clinical confirmation by a physician was required based on symptoms and clinical findings.

All samples were interpreted in conjunction with clinical presentation, imaging findings, CMT results, and tNGS results by at least two attending physicians. Given that all participants were hospitalized patients with clinically diagnosed respiratory infections, the presence of a pathogen detected by either CMTs or tNGS that was consistent with the clinical presentation was considered a true positive result for diagnostic comparison purposes. Samples in which no pathogen was detected by either method were classified as false negatives.

Treatment decisions in routine clinical practice were based on the combined interpretation of both CMT and tNGS results. However, to specifically evaluate the clinical impact attributable to tNGS, treatment modifications were retrospectively categorized according to the timing and diagnostic contribution of each test. In cases where tNGS identified a pathogen that was not detected by CMTs and the treatment adjustment occurred after the tNGS report became available, the change in therapy was considered primarily attributable to tNGS. In cases where both CMTs and tNGS detected the same pathogen, treatment adjustments were attributed to the test that first provided actionable results based on the reporting time documented in the medical record. When treatment decisions were made before the availability of tNGS results, these changes were considered to be guided by CMTs or clinical judgment rather than tNGS.

### 2.6 Evaluation of Clinical Effectiveness of tNGS

The clinical effectiveness of tNGS was assessed by evaluating its influence on treatment decisions and subsequent patient outcomes. Two independent attending physicians retrospectively reviewed each patient's medical records, including laboratory reporting times, treatment initiation records, and physician notes, to determine whether tNGS results contributed to therapeutic modifications.

Treatment adjustments were categorized into three groups: escalation, de‐escalation, or no change (Table S4). Escalation referred to the initiation or broadening of antimicrobial therapy based on pathogen identification, whereas de‐escalation referred to narrowing or discontinuation of antimicrobial therapy when bacterial infection was ruled out or a viral pathogen was identified.

To distinguish the impact of tNGS from conventional diagnostic methods, treatment adjustments were attributed to tNGS only when the following criteria were met:

1. The pathogen identified by tNGS was not detected by CMTs or was detected earlier by tNGS than by CMTs.
2. The treatment modification occurred after the release of the tNGS report.
3. Physician documentation indicated that the tNGS result contributed to the therapeutic decision.

If both CMTs and tNGS detected the same pathogen and treatment changes were initiated prior to the availability of the tNGS result, the adjustment was attributed to CMTs or clinical judgment rather than tNGS. Discrepancies between reviewers were resolved through discussion with a senior infectious disease specialist.

Patient outcomes following treatment adjustment were categorized as improvement, deterioration, or no significant change according to predefined clinical criteria (Table S4).

### 2.7 Statistical Analysis

The sensitivity, specificity, positive predictive value (PPV), and negative predictive value (NPV) of tNGS and CMTs were calculated based on their agreement with the final clinical diagnosis. Comparisons between groups were performed using the chi‐square test through an online calculator (https://www.shuxuele.com/data/chi‐square‐calculator.html).

It should be noted that predictive values are influenced by the prevalence of disease in the study population. Because this study included a cohort of hospitalized children with clinically suspected respiratory infections, the prior probability of infection was relatively high. Under such conditions, the NPV of diagnostic tests may appear lower despite high sensitivity and specificity. Therefore, PPV and NPV values reported in this study should be interpreted within the context of this high‐prevalence clinical population rather than as estimates applicable to general screening settings.

Data analysis was performed using SPSS software (Version 21.0; IBM Corp., Armonk, NY, USA), and results were visualized using the ggplot2 package in R [28]. All statistical tests were two‐tailed, and statistical significance was defined as *p* < 0.05.

## 3 Results

### 3.1 Patient Baseline Characteristics

Data were collected from pediatric patients diagnosed with RTIs at Qingdao Huangdao District People's Hospital between April 1, 2023, and December 31, 2023. A total of 1209 pediatric patients whose electronic medical records were available and who underwent tNGS testing during this period were initially identified (Figure 1). Of these, 269 patients were excluded due to multiple hospital visits or incomplete records of conventional pathogen testing. Ultimately, 940 patients were included in the final analysis.

Among the 940 patients, 785 (83.51%) were diagnosed with pneumonia, and 86 (9.15%) with severe pneumonia, whereas the remaining patients were diagnosed with other respiratory conditions such as tonsillitis and bronchitis (Table S5). The mean age of the patients was 4.83 years, and males accounted for 43.34% of the cohort. The average length of hospital stay was 6.14 days. Laboratory findings indicated that inflammatory markers were frequently elevated, with 91.80% of patients showing increased procalcitonin (PCT) levels and 64.68% presenting with elevated erythrocyte sedimentation rates (ESR) (Table 1).

**TABLE 1:** Demographic characteristics.

Characteristics	Total number (%)	Average	Outlier (%)
Age (year)	940 (100)	4.825	
Male	445 (43.34)		
Female	495 (52.66)		
Length of hospital stay (day)	940	6.14	
Blood laboratory examination (range)			
Neutrophil number, 10^9^/L (2–7)	442 (47.02)	3.88	165 (37.33)
Monocyte number, 10^9^/L (0.12–0.8)	442 (47.02)	0.56	65 (14.71)
Basophil number, 10^9^/L (0–0.1)	442 (47.02)	0.028	2 (0.45)
Eosinophil number, 10^9^/L (0.05–0.5)	442 (47.02)	0.11	204 (46.15)
Lymphocyte, 10^9^/L (0.8–4)	443 (47.12)	2.95	87 (19.64)
Procalcitonin, ng/mL (0–0.05)	841 (89.47)	0.30	772 (91.80)
IgE IU/mL (0–100)	590 (62.77)	201.13	263 (44.58)
Erythrocyte sedimentation rate, mm/h (0–15/20)	252 (26.81)	28.82	163 (64.68)

To evaluate the diagnostic performance of tNGS, we compared it individually with specific CMT components, including PCR, culture, and serology, rather than treating CMTs as a composite group. Based on known diagnostic limitations—such as the low specificity of IgM assays and the limited sensitivity of β‐D‐glucan testing in pediatric populations—these results were excluded from comparative analyses.

Among the 940 pediatric patients with RTIs, the overall pathogen detection rate was significantly higher using tNGS (88.2%) than PCR (38.7%, χ^2^ = 90.17, *p* < 0.001), culture (10.96%, χ^2^ = 139.86, *p* < 0.001), or serology (16.81%, χ^2^ = 87.38, *p* < 0.001). In patients with severe pneumonia, tNGS achieved a detection rate of 94.2%, which was significantly higher than that of PCR (44.2%, χ^2^ = 21.65, *p* < 0.001). Notably, tNGS identified potential pathogens in 67.5% of patients in whom conventional tests yielded negative results, suggesting improved diagnostic sensitivity.

Furthermore, tNGS detected polymicrobial infections (≥ 2 pathogens) in 41.5% of positive cases, compared with only 7.6% identified by conventional methods (χ^2^ = 122.81, *p* < 0.001). The most frequently detected pathogens by tNGS included respiratory syncytial virus (RSV), *Streptococcus pneumoniae* , *Haemophilus influenzae* , and *Moraxella catarrhalis* .

### 3.2 Diagnostic Performance of CMTs and tNGS

**FIGURE 2:**
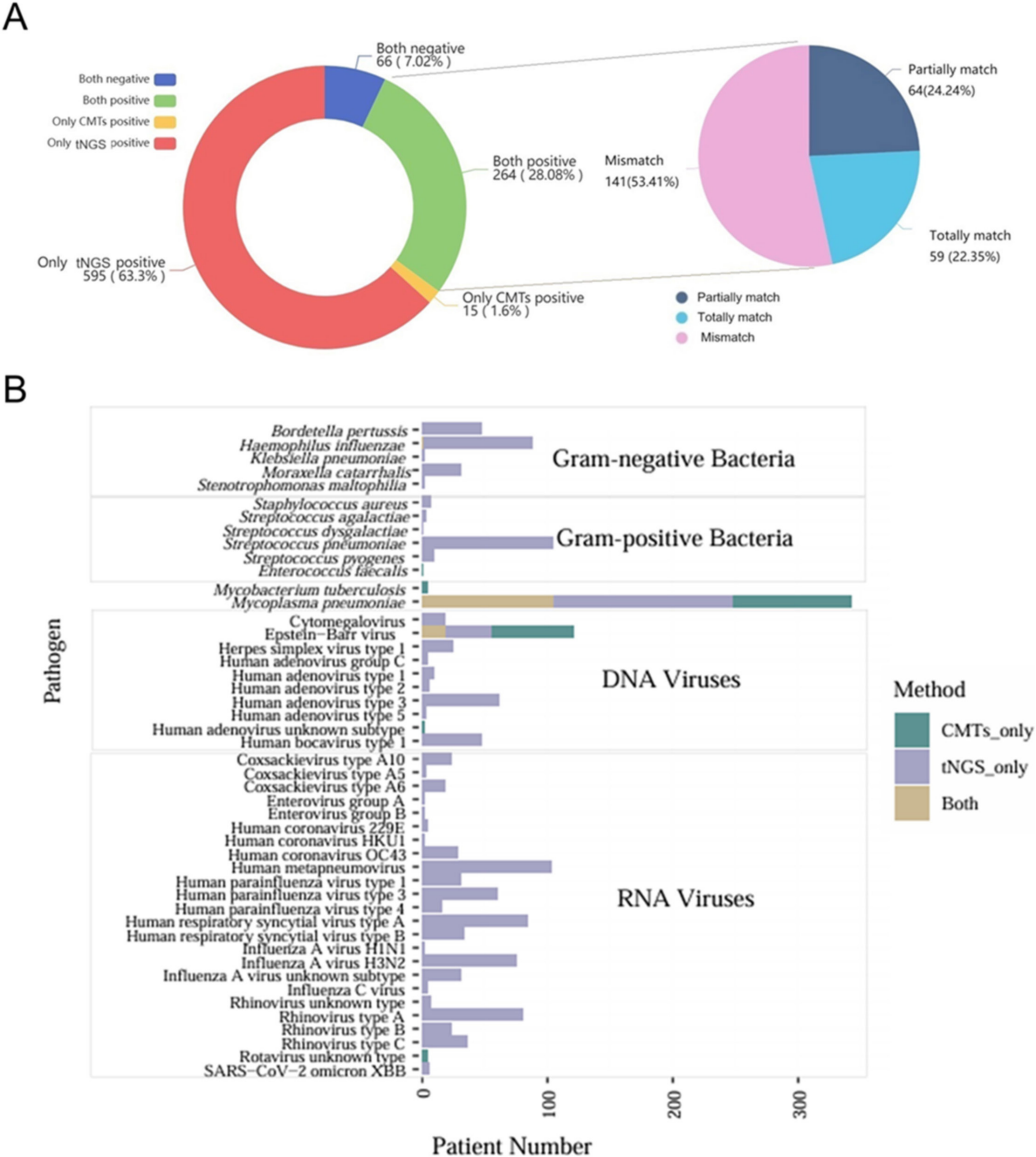
Consistency between CMTs and tNGS results. Among the 940 patients with RTIs, 595 patients (63.3%) were negative for CMTs but positive for tNGS, and 15 patients (1.6%) were positive for CMTs but negative for tNGS. Sixty‐six cases (7.02%) were negative for both CMTs and tNGS. Additionally, 264 patients (28.08%) were positive for both CMTs and tNGS.

All enrolled patients underwent both CMT and tNGS testing. The distribution of CMT methods is shown in Table S1. When comparing diagnostic outcomes, 28.08% of patients were identified as infected by both methods, among which 22.35% had concordant pathogen identification (Figure 2 and Table S4). However, discordant findings were common, with 53.41% of infected cases showing completely different pathogen profiles between the two approaches. Among these discordant cases, 63.3% were positive only by tNGS, whereas 1.6% were positive only by CMTs.

**FIGURE 3:**
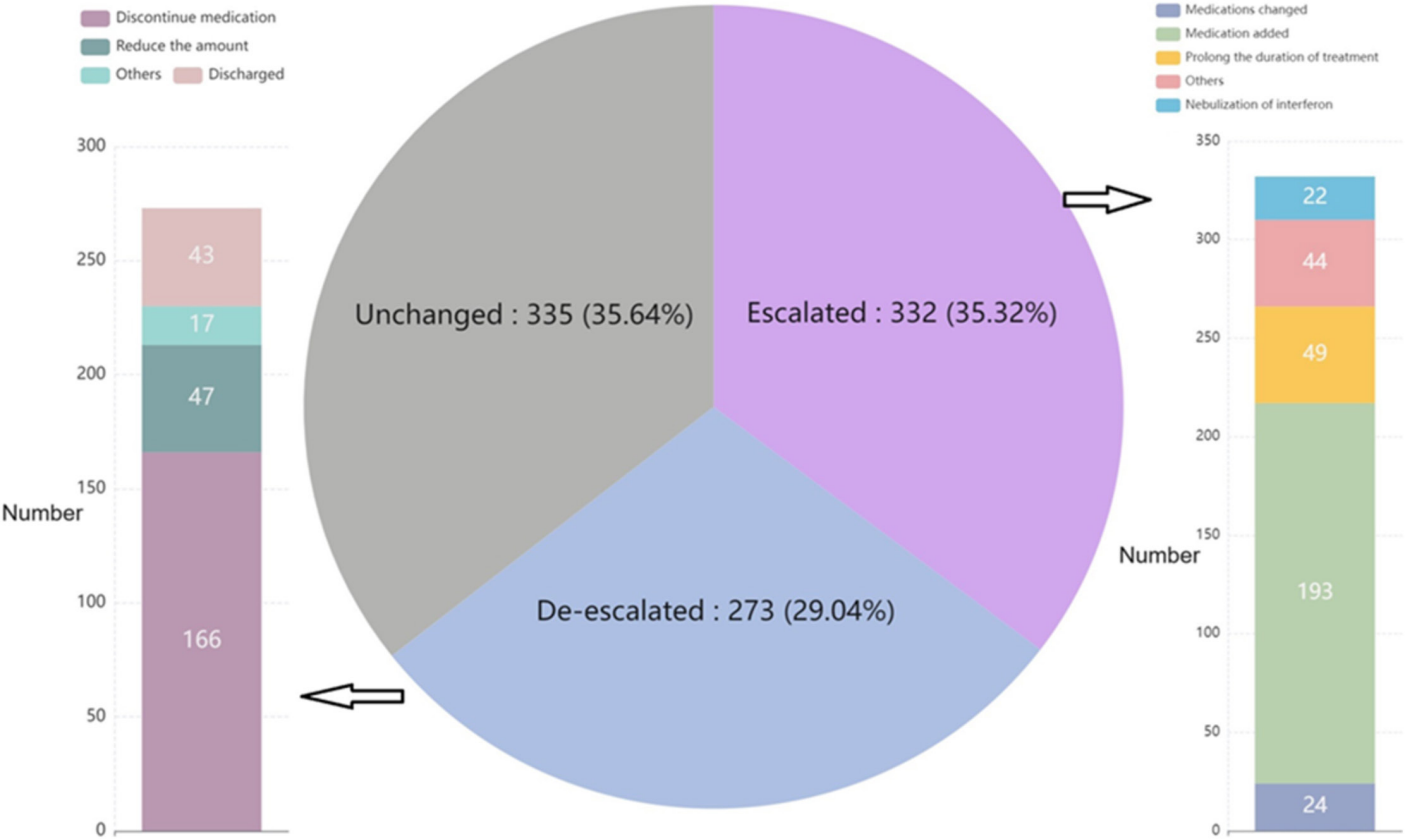
Detection performance of CMTs and tNGS at the pathogen level. A total of 47 pathogens were detected across all patients. tNGS demonstrated significant advantages over CMTs in detecting both viruses and bacteria. The *p* values were calculated using the chi‐square test, with *p* < 0.05 and *p* < 0.001 indicating statistical significance.

At the pathogen level, tNGS detected a significantly broader spectrum of organisms than CMTs, including both viral and bacterial pathogens (*p* < 0.05) (Figure 3). In total, 47 pathogens were identified. *M. pneumoniae* , *H. influenzae* , and Epstein–Barr virus were detected by both approaches in some patients; however, tNGS demonstrated significantly higher detection rates (*p* < 0.05). In contrast, *Enterococcus faecium* and *M. tuberculosis* were detected exclusively by CMTs. CMTs also reported untyped adenovirus strains in two patients and rotavirus in four patients that were not detected by tNGS. Notably, all RNA respiratory viruses detected in this study were identified by tNGS, which also allowed subtype‐level identification.

To further assess diagnostic consistency, tNGS results were compared with chest CT findings in cases where CMT results were negative or discordant. In several patients in whom CMTs failed to detect pathogens or detected non‐respiratory viruses (e.g., rotavirus), tNGS identified respiratory viruses such as RSV, adenovirus, or parainfluenza virus. These findings were consistent with CT imaging patterns suggestive of viral pneumonia, including ground‐glass opacities and bilateral interstitial infiltrates. In such cases, the concordance between tNGS findings and radiologic features supported clinical decisions to reduce or discontinue antibacterial therapy when bacterial coinfection was considered unlikely.

When compared with imaging‐based clinical diagnoses, the sensitivity of tNGS for detecting infections was 91.38%, whereas imaging alone demonstrated a sensitivity of 82.45%. Both methods showed substantially higher sensitivity than CMTs (29.68%, *p* < 0.001) (Table 2).

**TABLE 2:** Diagnostic sensitivity of the three methods.

Method	True positive	False negative	Sensitivity (%)	Specificity (%)	PPV (%)	NPV (%)
tNGS	859	81	91.38	0	98.85	−14.08
CMTs	279	661	29.68	0	96.54	−1.54
CT	775	96	88.98	85.51	98.73	38.06

### 3.3 Treatment Adjustment and Clinical Outcomes Associated With tNGS

Treatment strategies were modified during hospitalization based on the combined interpretation of clinical findings, CMT results, and tNGS reports. Using the predefined attribution criteria described in Section 2, treatment modifications that occurred after the release of tNGS results and were supported by tNGS pathogen identification were considered attributable to tNGS‐guided decision‐making.

Among the 940 patients, treatment escalation was observed in 332 cases (35.32%), including 193 cases in which new medications were introduced, 49 cases in which treatment duration was extended, and 90 cases involving other therapeutic interventions (Figure S1). In addition, treatment de‐escalation occurred in 273 patients (29.04%), including 166 cases involving discontinuation of antimicrobial agents, 47 cases involving dosage reduction, and 60 cases involving other adjustments. The remaining 335 patients (35.64%) did not experience changes in their treatment regimens.

Overall, most patients demonstrated clinical improvement following treatment modifications. Among patients whose therapy was escalated, three individuals experienced clinical deterioration, whereas the remaining patients improved. All patients who underwent treatment de‐escalation showed clinical improvement without evidence of deterioration (Table S5). The three patients who deteriorated were all infected with *M. pneumoniae* , including one case of refractory pneumonia and two cases of severe pneumonia.

These findings suggest that treatment modifications supported by tNGS results were generally associated with favorable clinical outcomes.

### 3.4 Differences in Pathogen Infections Across Age, Gender, and Seasons

Comparative analyses were conducted across demographic groups (Table S6). Twenty‐eight pathogens exhibited significant variation across age groups. The highest number of infections was observed among children aged 4–7 years (432 cases, 49.59%), followed by those aged 1–3 years (242 cases, 27.78%).

Certain pathogens demonstrated clear age‐related distribution patterns. Cytomegalovirus (*p* < 0.001) and respiratory syncytial virus type A (*p* < 0.001) were more frequently detected in infants younger than 1 year. Human bocavirus type 1 (*p* < 0.001) and rhinovirus type A (*p* < 0.001) were more common among children aged 1–3 years. *S. pneumoniae* (*p* < 0.001), human metapneumovirus (*p* < 0.001), and rhinovirus type C (*p* < 0.001) were most prevalent among children aged 4–7 years. In contrast, *M. pneumoniae* (*p* < 0.001), Epstein–Barr virus (*p* < 0.001), and influenza A H3N2 (*p* < 0.001) were significantly more common in children older than 7 years.

No significant gender differences were observed for most pathogens, except for *H. influenzae* , which was slightly more prevalent in male patients (*p* < 0.05), and human coronavirus 229E, which was more frequently detected in female patients (*p* < 0.05).

Seasonal variation in pathogen distribution was also observed. A total of 157 infections were detected during summer, 320 during autumn, and 394 during winter, suggesting an increasing trend from summer to winter. Eighteen pathogens showed significant seasonal variation (*p* < 0.001), with respiratory syncytial virus type A being particularly prevalent during winter.

### 3.5 TAT and Clinical Decision‐Making

The average reporting TAT for tNGS in this study was approximately 28.5 h. This duration was shorter than that required for conventional culture‐based diagnostics (> 72 h) and slightly longer than routine PCR assays (typically 12–24 h). The workflow included approximately 2 h for nucleic acid extraction, 8 h for library construction, 10 h for target enrichment, 6 h for sequencing, and 2.5 h for bioinformatic analysis.

**FIGURE 4:**
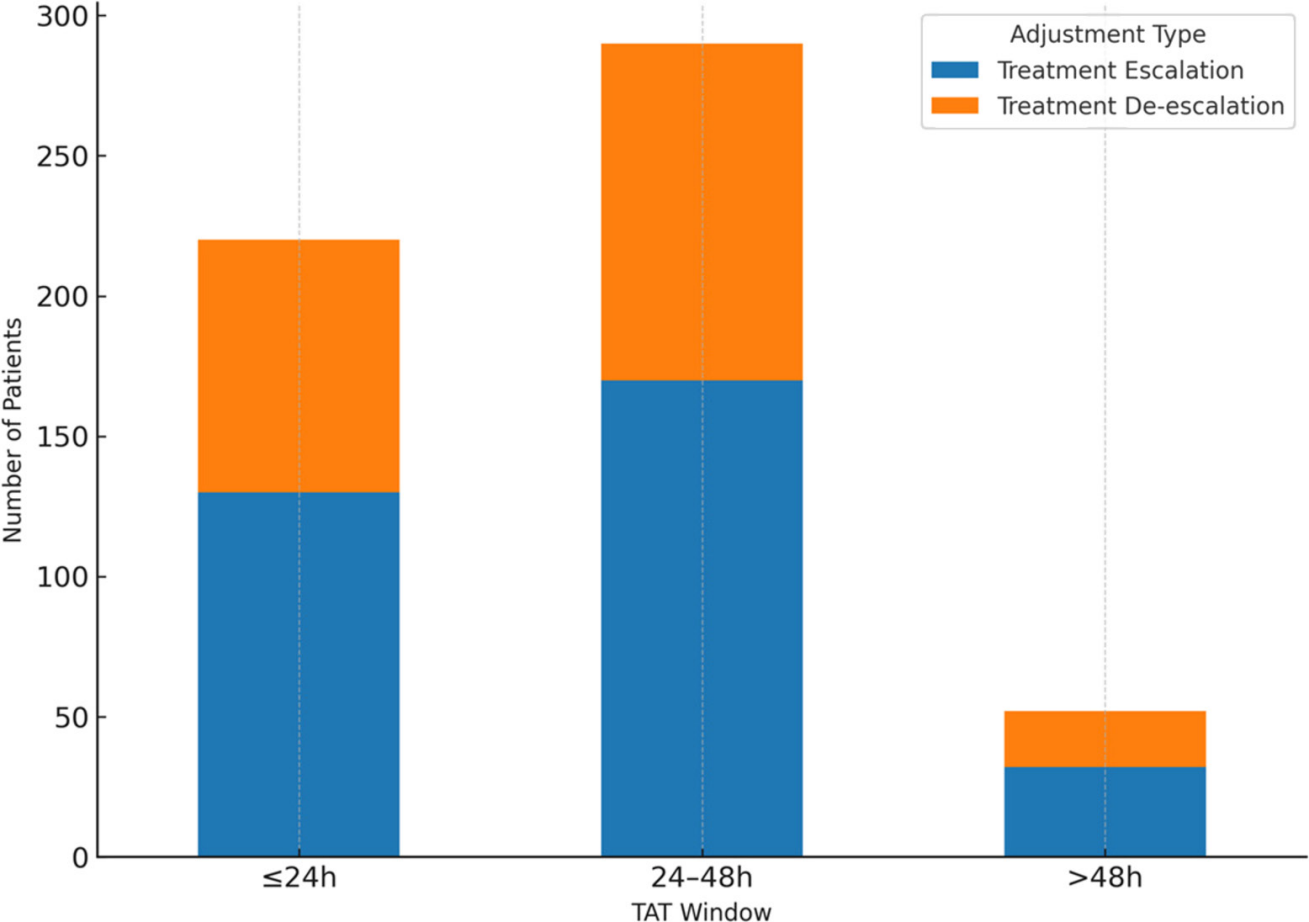
Distribution of treatment adjustments by TAT window. The majority of treatment escalations (*n* = 130 + 170) and de‐escalations (*n* = 90 + 120) occurred within 48 h of admission, aligning with the average tNGS reporting time of 28.5 h. This supports the clinical utility of tNGS for timely therapeutic decision‐making in pediatric respiratory tract infections.

Among patients who underwent treatment escalation or de‐escalation, most therapeutic modifications occurred within the first 48 h after hospital admission. As shown in Figure 4, the majority of treatment adjustments occurred within the ≤ 24‐h and 24–48‐h intervals following diagnostic reporting. These observations suggest that the relatively rapid availability of tNGS results allowed clinicians to incorporate sequencing findings into early therapeutic decision‐making in selected cases.

Notably, no clinical deterioration was observed among patients whose antimicrobial therapy was de‐escalated following pathogen identification consistent with viral infection.

## 4 Discussion

RTIs in pediatric patients carry significant long‐term risks, including wheezing, asthma, and impaired lung function. Accurate pathogen identification is therefore essential for guiding appropriate therapy and improving clinical outcomes. tNGS has emerged as a promising diagnostic approach that may provide higher pathogen detection rates than CMTs. However, evidence regarding its real‐world clinical utility in pediatric respiratory infections remains limited. In this study, we investigated the diagnostic performance and clinical impact of tNGS in 940 hospitalized children with RTIs over a 9‐month period in northern China.

The 28.5‐h average TAT of tNGS provided several tangible clinical benefits. First, therapeutic precision was improved: nearly 90% of escalated therapies (e.g., macrolide treatment for *M. pneumoniae* ) were initiated within 48 h after the availability of tNGS results, which aligns with evidence that early targeted therapy improves outcomes in pediatric pneumonia [3, 4]. Second, antimicrobial stewardship was enhanced, as treatment de‐escalation decisions occurred earlier than with culture‐dependent diagnostic pathways, thereby reducing unnecessary antibiotic exposure. Third, resource utilization was improved when tNGS results ruled out bacterial coinfection, allowing clinicians to discontinue antibiotics and shorten hospital stays in patients with viral infections. These findings suggest that the clinical value of tNGS extends beyond improved diagnostic sensitivity and contributes to real‐time clinical decision‐making.

When comparing TAT, it should be noted that routine PCR assays may produce results within 6–24 h depending on laboratory workflows, particularly in urgent testing scenarios. Therefore, the advantage of tNGS should not be interpreted primarily in terms of speed. Instead, its major strength lies in its broader pathogen coverage and hypothesis‐free detection capability, enabling the simultaneous identification of a wide range of pathogens, including uncommon or unexpected organisms.

Whereas previous studies often group CMTs together for analysis, our findings highlight the importance of disaggregating individual conventional diagnostic methods. In our cohort, tNGS demonstrated significantly higher sensitivity than each individual CMT component, including PCR, culture, and serological assays. For example, tNGS showed substantially higher sensitivity than PCR (tNGS: 88.2% vs. PCR: 38.7%, *p* < 0.001). Interestingly, pathogens such as *E. faecium* and *M. tuberculosis* (MTB) were detected only by CMTs but not by tNGS, despite being included in the tNGS panel. This discrepancy suggests that these CMT detections are unlikely to represent true respiratory infections. *E. faecium* is not recognized as a typical respiratory pathogen and is more commonly associated with gastrointestinal colonization or healthcare‐associated infections. Similarly, detection of MTB from throat swab specimens warrants particular caution. Throat swabs are anatomically part of the upper respiratory tract, and current evidence indicates that their sensitivity and specificity for detecting MTB are markedly lower than those of lower airway or sputum specimens [29]. Consequently, a positive MTB signal from such samples may more plausibly reflect contamination, transient colonization, or detection of nonviable DNA rather than active pulmonary tuberculosis. Therefore, such results should be interpreted with extreme caution and require confirmation using appropriate diagnostic methods such as sputum culture, GeneXpert, or BALF‐based assays. These considerations reinforce the importance of interpreting molecular diagnostic results within the broader clinical, radiological, and epidemiological context.

In addition, the role of syndromic multiplex PCR respiratory panels should be acknowledged. These panels—such as FilmArray Respiratory Panel, ePlex Respiratory Pathogen Panel, and Luminex NxTAG—have been widely implemented in clinical microbiology laboratories for nearly a decade. They enable simultaneous detection of multiple common bacterial, viral, and fungal respiratory pathogens within approximately 1.5–2 h, offering high analytical sensitivity and specificity with standardized external quality assurance [30, 31]. Compared with these well‐established panels, tNGS provides broader and hypothesis‐free detection capacity, including uncommon, emerging, or novel pathogens not covered by fixed PCR panels. However, the higher cost and longer TAT of tNGS currently limit its widespread routine use. In clinical practice, multiplex PCR panels remain the preferred first‐line diagnostic tools for most suspected RTIs. tNGS may therefore be most appropriately positioned as a complementary or second‐line diagnostic strategy, particularly in patients with severe disease, atypical clinical presentations, immunocompromised status, or persistent symptoms despite negative or inconclusive results from conventional microbiological testing or multiplex PCR panels.

Importantly, we also examined the clinical outcomes associated with tNGS‐guided treatment adjustments. Among patients tested with tNGS, 35.3% (*n* = 332) experienced treatment escalation, and most showed clinical improvement, with only three cases deteriorating. In contrast, 24.5% (*n* = 230) underwent treatment de‐escalation without adverse outcomes. These findings support the clinical utility of tNGS not only in pathogen detection but also in optimizing therapeutic decisions by enabling more targeted antimicrobial therapy and reducing unnecessary antibiotic exposure in viral infections.

Our study revealed that the majority of patients exhibited abnormal blood test results, with 91.8% showing elevated PCT levels and 64.68% having an accelerated ESR (Table 1). PCT, which is relatively stable and not influenced by hormone levels, has been widely used as a biomarker for distinguishing bacterial from nonbacterial infections and for evaluating the severity of inflammatory diseases [32]. Previous studies have shown that elevated PCT levels correlate with infection severity and may provide prognostic information in infectious diseases [33, 34, 35]. However, although PCT is commonly used as an indicator of bacterial infection, our data demonstrated that elevated PCT levels were also observed in some viral or *M. pneumoniae* infections. In these cases, the magnitude of elevation generally appeared lower than that typically observed in confirmed bacterial infections. Previous studies suggest that certain pathogens, particularly *M. pneumoniae* , may induce moderate PCT elevation due to systemic inflammatory responses. These findings indicate that although PCT testing is sensitive for detecting infection severity, its specificity for differentiating bacterial from nonbacterial respiratory infections may be limited when used in isolation [36]. Elevated ESR, which can occur in a variety of inflammatory conditions including pneumonia, active tuberculosis, rheumatic fever, severe anemia, and acute infections, is considered a nonspecific marker of inflammation [37]. Although 64.68% of children in our study exhibited elevated ESR levels, this marker alone lacks sufficient sensitivity and specificity to reliably indicate pediatric respiratory infections.

mNGS has been widely applied in RTI studies and has demonstrated superior sensitivity compared with CMTs. In contrast, tNGS, based on probe‐capture technology, improves the proportion of target reads, processing efficiency, and cost‐effectiveness. In the present study, tNGS exhibited significantly higher sensitivity (91.38%) for pathogen detection compared with CMTs (29.68%) (Table 2), demonstrating performance comparable to mNGS and superior to CMTs [38, 39]. In terms of pathogen detection, tNGS identified a broader spectrum of pathogens, including low‐abundance organisms and RNA viruses that are frequently missed by conventional diagnostic methods (Figure 3). Even among pathogens detected by both approaches, tNGS demonstrated significantly higher sensitivity than CMTs (*p* < 0.05). In addition, tNGS frequently detected multiple pathogens within a single patient, whereas CMTs typically identified only one pathogen, consistent with findings reported in other studies [40, 41, 42].

It should also be noted that certain pathogens were detected exclusively by CMTs because they were not included in the predefined tNGS panel. For example, rotavirus was detected only by CMTs in our study. Rotavirus is primarily recognized as an etiologic agent of gastroenteritis rather than RTIs [42]. Therefore, its occasional detection in respiratory samples is most likely attributable to contamination from the gastrointestinal tract or transient viral shedding rather than true respiratory infection. Current literature provides limited evidence supporting a pathogenic role for rotavirus in respiratory disease. This observation highlights the deliberate and evidence‐based design of our tNGS panel, which focuses on pathogens with well‐established roles in respiratory infections, thereby maintaining diagnostic specificity and clinical relevance. Nevertheless, expanding the pathogen coverage of tNGS panels in future studies may further enhance their diagnostic utility.

Respiratory pathogens often exhibit seasonal variation [43]. A previous epidemiological study conducted in Guangzhou, a southern city in China, reported that human adenovirus infections were most common during summer months [44]. In contrast, our study observed a higher proportion of adenovirus infections during autumn and winter, which may reflect climatic differences between northern and southern regions of China. The seasonal trend of influenza virus observed in our study is consistent with findings from a long‐term epidemiological investigation in China [45]. Because the immune systems of children are still developing, age‐related differences in susceptibility to respiratory pathogens are commonly observed. Numerous studies have shown that infants and preschool‐aged children are particularly vulnerable to acute respiratory infections [44, 45, 46]. Consistent with these findings, our study found the highest incidence of RTIs among preschool‐aged children (1–6 years). No significant differences in infection rates were observed between male and female patients, which is consistent with previous reports [46].

In a subset analysis using chest CT findings as a reference for radiologic consistency, tNGS was able to detect viral pathogens such as respiratory syncytial virus, adenovirus, and parainfluenza virus that were not identified by CMTs. These results were consistent with CT features suggestive of viral pneumonia. In contrast, CMTs occasionally detected organisms with questionable respiratory pathogenicity or failed to identify pathogens in these cases. These observations suggest that tNGS may help guide therapy de‐escalation when clinical and radiological findings support a viral etiology.

Several limitations of this study should be acknowledged. First, the study population consisted predominantly of hospitalized children with clinically significant respiratory infections, as reflected by the high proportion of pneumonia diagnoses (83.51%) and elevated PCT levels (91.80%). This indicates a cohort with a relatively high pretest probability of infection. Consequently, the diagnostic yield and clinical impact of tNGS observed in this study may be greater than what might be expected in a broader pediatric population with milder or undifferentiated RTIs. Therefore, the generalizability of these findings to outpatient settings or mild community‐acquired infections should be interpreted with caution. Second, throat swab specimens were used for tNGS analysis. Although throat swabs are noninvasive and easier to obtain in pediatric populations, they represent upper respiratory tract samples and may not fully reflect the microbial composition of the lower respiratory tract. Bronchoalveolar lavage fluid remains the gold‐standard specimen for identifying pathogens responsible for lower RTIs, particularly pneumonia. However, BALF collection is invasive and often not clinically feasible in young children with mild to moderate illness. Therefore, our findings primarily reflect upper respiratory microbial profiles. Additional limitations include the single‐center retrospective design and the lack of longitudinal follow‐up to evaluate long‐term outcomes after treatment adjustment. Furthermore, similar to PCR‐based diagnostics, tNGS may detect nucleic acids from nonviable organisms or prolonged viral shedding, particularly for viruses such as adenovirus or rhinovirus that may persist for several weeks after acute infection. Therefore, molecular detection results should always be interpreted in conjunction with clinical symptoms, imaging findings, and epidemiological context.

## 5 Conclusion

Although tNGS offers clear advantages in pathogen detection breadth and diagnostic sensitivity, the technology has not yet been fully standardized for routine clinical use. At present, tNGS should be considered an adjunctive diagnostic approach that complements existing microbiological methods, particularly in complex, severe, or diagnostically challenging infections. Future multicenter studies, interlaboratory standardization efforts, and cost‐effectiveness analyses will be essential to define the optimal role of tNGS in clinical microbiology and pediatric infectious disease management.

## Author Contributions

L.W., L.Y., H.Z., and X.Y. designed the study and wrote and revised the manuscript. H.Z. and Y.F. were involved in collecting, analyzing, or interpreting research data and writing the manuscript. L.W. and L.Y. analyzed research data. All authors contributed to the article and approved the submitted version.

## Funding

The authors have nothing to report.

## Ethics Statement

The study protocol was approved by the Ethics Committee of Qingdao Huangdao District People's Hospital (LL‐LW‐2024001). Written informed consent was obtained from all adult participants or their legal guardians for participation in this study.

## Consent

As authors of this manuscript, we hereby give our full consent for its submission and publication.

## Conflicts of Interest

The authors declare no conflicts of interest.

## Supporting information


**Figure S1:** Supporting Information.

**Table S1:** RPhK assigned to pathogens for each patient.

**Table S2:** Clinical diagnosis, treatment, and outcomes for collected patients.

**Table S3:** RPhK assigned to pathogens for each patient.

**Table S4:** Criteria of treatment plan adjustments and response based on tNGS diagnosis.

**Table S5:** Clinical diagnosis, treatment, and outcomes for collected patients.

**Table S6:** Statistic of *p* values across different comparison groups.

## Data Availability

The targeted next‐generation sequencing data have been deposited in the China National GeneBank Sequence Archive (CNSA; https://db.cngb.org/cnsa/; 10.1093/h/uhaf036) under BioProject accession number CNP0005922 (https://db.cngb.org/data_resources/project/CNP0005922). Additional data supporting the findings of this study are available from the corresponding author upon reasonable request.
