# Supplementary figures and images for: Diagnostic and Clinical Value of Targeted Next‐Generation Sequencing for Pediatric Respiratory Infections in Northern China
Source: Clin Respir J. 2026 Apr 12;20(4):e70185. doi: 10.1111/crj.70185 (PMC13070711; doi:10.1111/crj.70185)

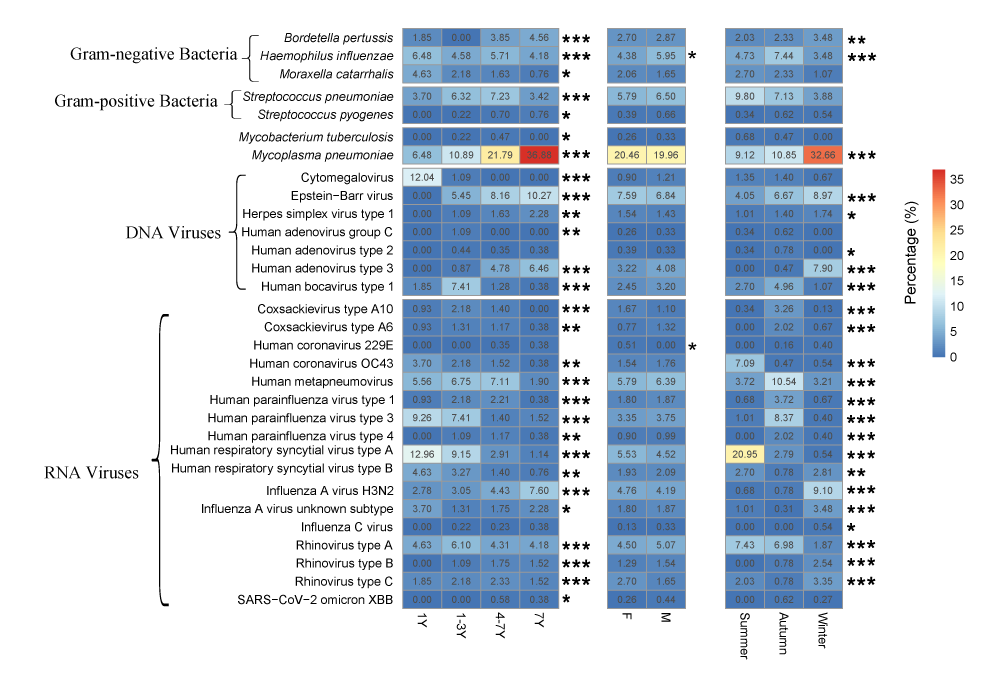

Supplement: Supplementary file 1 — Figure S1: Supporting Information. [file CRJ-20-e70185-s001.tif]
