# Supplementary material for: Diagnostic and Clinical Value of Targeted Next‐Generation Sequencing for Pediatric Respiratory Infections in Northern China
Source: Clin Respir J. 2026 Apr 12;20(4):e70185. doi: 10.1111/crj.70185 (PMC13070711; doi:10.1111/crj.70185)
Supplement: Supplementary file 2 — Table S1: RPhK assigned to pathogens for each patient. [file CRJ-20-e70185-s004.docx]

| **Supplementary Table 1. RPhK assigned to pathogens for each patient.** | | |
| --- | --- | --- |
| **Patient ID** | **Pathogens** | **RPhK** |
| 1031572 | *Stenotrophomonas maltophilia* | 152 |
| 1029455 | *Staphylococcus aureus* | 125 |
|  | *Streptococcus intermedius* | 22867 |
|  | *Streptococcus constellatus* | 16778 |
| 1029877 | *Human Metapneumovirus (HMPV)* | 55479 |
|  | *Haemophilus influenzae* | 3745 |
| 1029888 | *Human respiratory syncytial virus type A* | 76239 |
|  | *Moraxella catarrhalis* | 171 |
|  | *Streptococcus anginosus* | 4212 |
| 1029880 | *Rhinovirus A* | 53925 |
|  | *Haemophilus influenzae* | 876 |
|  | *Streptococcus intermedius* | 505 |
| 952541 | *Bordetella pertussis* | 38 |
|  | *Haemophilus influenzae* | 38 |
|  | *Streptococcus intermedius* | 486 |
| 907729 | *Streptococcus pneumoniae* | 575 |
|  | *Streptococcus anginosus* | 32048 |
| 1029909 | *Human respiratory syncytial virus type A* | 667 |
| 1029893 | *Rhinovirus A* | 14478 |
|  | *Haemophilus influenzae* | 47452 |
|  | *Streptococcus pneumoniae* | 4629 |
| 1029898 | *Human bocavirus type 1* | 47611 |
| 1024095 | *Rhinovirus type C* | 35033 |
|  | *Human respiratory syncytial virus type A* | 20010 |
|  | *Moraxella catarrhalis* | 16980 |
|  | *Streptococcus intermedius* | 11116 |
|  | *Streptococcus pneumoniae* | 309 |
| 1029073 | *Human respiratory syncytial virus type A* | 16844 |
|  | *Streptococcus constellatus* | 2928 |
|  | *Streptococcus pneumoniae* | 337 |
| 1029063 | *Streptococcus anginosus* | 1430 |
|  | *Human respiratory syncytial virus type A* | 716 |
|  | *Streptococcus pneumoniae* | 100 |
| 1029076 | *Human respiratory syncytial virus type A* | 2609 |
| 1029111 | *Human respiratory syncytial virus type A* | 65064 |
|  | *Streptococcus pneumoniae* | 384 |
| 1029144 | *Human respiratory syncytial virus type A* | 55716 |
|  | *Human coronavirus OC43* | 4195 |
|  | *Streptococcus pneumoniae* | 1000 |
| 931421 | *Rhinovirus A* | 24088 |
| 1029095 | *Streptococcus pneumoniae* | 10075 |
|  | *Haemophilus influenzae* | 9524 |
|  | *Human respiratory syncytial virus type B* | 93 |
| 1029164 | *Human respiratory syncytial virus type A* | 6604 |
|  | *Streptococcus pneumoniae* | 2534 |
| 1021620 | *Human coronavirus OC43* | 7326 |
| 1029192 | *Klebsiella pneumoniae* | 110 |
| 1029815 | *Human Metapneumovirus (HMPV)* | 5737 |
|  | *Human adenovirus type 2* | 43 |
| 1029826 | *Rhinovirus A* | 57 |
|  | *Streptococcus pneumoniae* | 81 |
| 1029808 | *Rhinovirus A* | 20455 |
|  | *Human adenovirus type 1* | 98 |
|  | *Haemophilus influenzae* | 54983 |
| 1023832 | *Rhinovirus type C* | 61883 |
|  | *Streptococcus anginosus* | 9141 |
|  | *Haemophilus influenzae* | 126 |
| 1023835 | *Streptococcus pneumoniae* | 32343 |
|  | *Human respiratory syncytial virus type A* | 28474 |
|  | *Influenza A virusH1N1(2009)* | 13411 |
|  | *Moraxella catarrhalis* | 45 |
| 1029334 | *Mycoplasma pneumoniae* | 10931 |
| 989903 | *Human coronavirus OC43* | 73620 |
|  | *Streptococcus pneumoniae* | 5087 |
| 1029330 | *Rhinovirus A* | 1519 |
|  | *Streptococcus constellatus* | 537 |
| 1029331 | *Human coronavirus OC43* | 47707 |
|  | *Rhinovirus A* | 22161 |
| 1029332 | *Rhinovirus* | 48262 |
| 1029341 | *Moraxella catarrhalis* | 1820 |
|  | *Streptococcus constellatus* | 1752 |
|  | *Staphylococcus aureus* | 966 |
| 1010239 | *Human respiratory syncytial virus type A* | 404 |
|  | *Haemophilus influenzae* | 52 |
| 1029692 | *Haemophilus influenzae* | 1781 |
|  | *Streptococcus intermedius* | 408 |
| 1026556 | *Streptococcus intermedius* | 11206 |
| 1029628 | *Human respiratory syncytial virus type A* | 22003 |
|  | *Streptococcus intermedius* | 994 |
| 1008630 | *Rhinovirus A* | 69 |
|  | *Streptococcus intermedius* | 15496 |
| 948385 | *Human respiratory syncytial virus type A* | 40060 |
|  | *Coxsackievirus type A10* | 11529 |
|  | *Mycoplasma pneumoniae* | 84 |
|  | *Streptococcus pneumoniae* | 15818 |
| 1029603 | *Human coronavirus OC43* | 77 |
|  | *Streptococcus constellatus* | 6466 |
|  | *Streptococcus intermedius* | 3820 |
| 960973 | *Streptococcus pneumoniae* | 9022 |
|  | *Haemophilus influenzae* | 1426 |
|  | *Streptococcus constellatus* | 38477 |
|  | *Streptococcus anginosus* | 27957 |
| 1030297 | *Streptococcus agalactiae* | 35 |
|  | *Human coronavirus OC43* | 27 |
| 1030303 | *Human Metapneumovirus (HMPV)* | 14316 |
|  | *Haemophilus influenzae* | 34593 |
|  | *Streptococcus constellatus* | 30253 |
| 1030311 | *Mycoplasma pneumoniae* | 29810 |
| 1030200 | *Streptococcus constellatus* | 1495 |
| 1030321 | *Human Metapneumovirus (HMPV)* | 15196 |
|  | *Streptococcus constellatus* | 3054 |
| 1030372 | *Rhinovirus A* | 40880 |
|  | *Bordetella pertussis* | 12940 |
|  | *Streptococcus pneumoniae* | 26658 |
|  | *Haemophilus influenzae* | 247 |
| 1020418 | *Human coronavirus OC43* | 18154 |
|  | *Streptococcus anginosus* | 2536 |
| 1030384 | *Streptococcus constellatus* | 2719 |
|  | *Streptococcus intermedius* | 184 |
|  | *Acinetobacter baumannii* | 114 |
| 1025631 | *Human respiratory syncytial virus type A* | 40414 |
|  | *Streptococcus constellatus* | 92 |
|  | *Acinetobacter baumannii* | 72 |
| 1028896 | *Staphylococcus aureus* | 6662 |
|  | *Human respiratory syncytial virus type B* | 6355 |
|  | *Streptococcus pneumoniae* | 2542 |
| 1028909 | *Human respiratory syncytial virus type A* | 21242 |
|  | *Rhinovirus A* | 240 |
|  | *Streptococcus pneumoniae* | 83 |
| 1018349 | *Streptococcus constellatus* | 725 |
|  | *Streptococcus pneumoniae* | 400 |
| 1028904 | *Streptococcus anginosus* | 1504 |
|  | *Human respiratory syncytial virus type A* | 1332 |
| 1029651 | *Rhinovirus A* | 20967 |
|  | *Haemophilus influenzae* | 241 |
|  | *Streptococcus intermedius* | 995 |
| 1029709 | *Human respiratory syncytial virus type A* | 310 |
| 1029804 | *Human coronavirus OC43* | 15391 |
|  | *Rhinovirus A* | 142 |
|  | *Streptococcus pneumoniae* | 2465 |
| 1029735 | *Human respiratory syncytial virus type A* | 69671 |
|  | *Streptococcus intermedius* | 5061 |
| 1029705 | *Human parainfluenza virus type 1* | 37613 |
|  | *Moraxella catarrhalis* | 27065 |
|  | *Streptococcus pneumoniae* | 2116 |
|  | *Haemophilus influenzae* | 866 |
|  | *Streptococcus constellatus* | 21367 |
| 1030201 | *Human parainfluenza virus type 3* | 54802 |
| 1029214 | *Human respiratory syncytial virus type B* | 79233 |
| 1029240 | *Streptococcus constellatus* | 58 |
| 1029244 | *Streptococcus intermedius* | 3233 |
| 1029256 | *Human respiratory syncytial virus type A* | 74448 |
|  | *Streptococcus constellatus* | 2210 |
| 1029259 | *Streptococcus constellatus* | 507 |
| 1024632 | *Streptococcus anginosus* | 19028 |
|  | *Streptococcus constellatus* | 10031 |
|  | *Influenza A virus H3N2* | 4576 |
| 985342 | *Streptococcus intermedius* | 3255 |
|  | *Streptococcus constellatus* | 3015 |
|  | *Rhinovirus A* | 2077 |
|  | *Haemophilus influenzae* | 791 |
| 1005193 | *Streptococcus intermedius* | 1551 |
|  | *Streptococcus constellatus* | 770 |
|  | *Rhinovirus A* | 59 |
| 1026570 | *Human respiratory syncytial virus type A* | 56325 |
|  | *Streptococcus constellatus* | 498 |
|  | *Staphylococcus aureus* | 109 |
| 1026596 | *Influenza A virus* | 29416 |
|  | *Moraxella catarrhalis* | 3027 |
|  | *Acinetobacter baumannii* | 290 |
| 1026630 | *Human respiratory syncytial virus type A* | 88430 |
|  | *Streptococcus pneumoniae* | 224 |
| 1026562 | *Human respiratory syncytial virus type A* | 3658 |
|  | *Acinetobacter baumannii* | 165 |
| 1024908 | *Streptococcus constellatus* | 41949 |
|  | *Streptococcus pneumoniae* | 17333 |
|  | *Bordetella pertussis* | 9636 |
|  | *Human adenovirus type 1* | 5611 |
| 882943 | *Rhinovirus type C* | 68994 |
|  | *Haemophilus influenzae* | 8945 |
|  | *Streptococcus intermedius* | 109 |
| 1025209 | *Human respiratory syncytial virus type A* | 74336 |
|  | *Streptococcus pneumoniae* | 3820 |
|  | *Moraxella catarrhalis* | 1112 |
|  | *Streptococcus intermedius* | 538 |
|  | *Staphylococcus aureus* | 96 |
| 1025639 | *Influenza A virus* | 1098 |
|  | *Acinetobacter baumannii* | 438 |
| 1024190 | *Streptococcus anginosus* | 45071 |
|  | *Streptococcus constellatus* | 10103 |
|  | *Influenza A virusH1N1(2009)* | 105 |
| 1024306 | *Influenza A virus H3N2* | 53805 |
|  | *Streptococcus pyogenes* | 14892 |
|  | *Streptococcus anginosus* | 6454 |
|  | *Streptococcus constellatus* | 4836 |
|  | *Streptococcus pneumoniae* | 3848 |
|  | *Moraxella catarrhalis* | 357 |
|  | *Haemophilus influenzae* | 45 |
| 1024336 | *Human respiratory syncytial virus type A* | 46996 |
|  | *Streptococcus intermedius* | 3885 |
|  | *Streptococcus constellatus* | 3352 |
| 1025090 | *Human respiratory syncytial virus type A* | 52347 |
|  | *Streptococcus intermedius* | 8708 |
|  | *Streptococcus constellatus* | 6207 |
| 1024910 | *Streptococcus intermedius* | 560 |
| 1025201 | *Human respiratory syncytial virus type A* | 40736 |
|  | *Streptococcus pneumoniae* | 5113 |
|  | *Haemophilus influenzae* | 402 |
| 1025251 | *Rhinovirus type C* | 1077 |
|  | *Streptococcus constellatus* | 137 |
|  | *Haemophilus influenzae* | 39 |
| 1026733 | *Human respiratory syncytial virus type A* | 65785 |
|  | *Streptococcus pneumoniae* | 4306 |
| 1011445 | *Human respiratory syncytial virus type A* | 32803 |
|  | *Streptococcus intermedius* | 2145 |
| 1026467 | *Human respiratory syncytial virus type A* | 129 |
| 1026394 | *Rhinovirus A* | 153 |
|  | *Herpes simplex virus type 1* | 48 |
| 1026370 | *Acinetobacter baumannii* | 130 |
|  | *Human respiratory syncytial virus type A* | 77 |
| 1025558 | *Human respiratory syncytial virus type A* | 28535 |
|  | *Streptococcus constellatus* | 1050 |
|  | *Streptococcus anginosus* | 706 |
|  | *Staphylococcus aureus* | 21 |
| 1028993 | *Human coronavirus OC43* | 609 |
|  | *Haemophilus influenzae* | 164 |
| 1028991 | *Human respiratory syncytial virus type B* | 83347 |
| 970345 | *Streptococcus pneumoniae* | 7625 |
|  | *Human respiratory syncytial virus type A* | 2234 |
| 1028972 | *Human coronavirus OC43* | 18741 |
|  | *Streptococcus pneumoniae* | 9806 |
|  | *Moraxella catarrhalis* | 850 |
|  | *Klebsiella pneumoniae* | 343 |
|  | *Staphylococcus aureus* | 239 |
| 1026195 | *Mycoplasma pneumoniae* | 4954 |
|  | *Streptococcus pneumoniae* | 17 |
| 1026294 | *Human respiratory syncytial virus type A* | 31208 |
| 1028009 | *Human respiratory syncytial virus type A* | 40095 |
| 1028017 | *Human respiratory syncytial virus type A* | 78134 |
| 1028116 | *Human bocavirus type 1* | 80868 |
|  | *Streptococcus pneumoniae* | 267 |
| 1028123 | *Human coronavirus OC43* | 6609 |
|  | *Streptococcus intermedius* | 1451 |
|  | *Haemophilus influenzae* | 371 |
| 1025466 | *Rhinovirus A* | 40137 |
|  | *Acinetobacter baumannii* | 1218 |
|  | *Moraxella catarrhalis* | 1024 |
|  | *Staphylococcus aureus* | 193 |
| 1026727 | *Streptococcus intermedius* | 14444 |
|  | *Streptococcus constellatus* | 13836 |
|  | *Influenza A virus* | 385 |
| 1008417 | *Human respiratory syncytial virus type A* | 60410 |
|  | *Acinetobacter baumannii* | 1128 |
|  | *Streptococcus intermedius* | 645 |
| 1027925 | *Streptococcus anginosus* | 5741 |
|  | *Haemophilus influenzae* | 3875 |
|  | *Human coronavirus OC43* | 574 |
| 1027910 | *Streptococcus anginosus* | 376 |
| 1004262 | *Streptococcus anginosus* | 15045 |
|  | *Human respiratory syncytial virus type A* | 713 |
| 1027286 | *Human respiratory syncytial virus type A* | 23887 |
|  | *Streptococcus intermedius* | 7072 |
| 1025713 | *Streptococcus intermedius* | 1340 |
|  | *Streptococcus pneumoniae* | 1214 |
| 1028532 | *Human respiratory syncytial virus type B* | 56246 |
|  | *Streptococcus pneumoniae* | 20327 |
| 1028598 | *Acinetobacter baumannii* | 381 |
|  | *Streptococcus anginosus* | 328 |
|  | *Human Metapneumovirus (HMPV)* | 116 |
| 925496 | *Rhinovirus A* | 32923 |
| 1028706 | *Acinetobacter baumannii* | 541 |
|  | *Pseudomonas aeruginosa* | 87 |
| 1028419 | *Human bocavirus type 1* | 33408 |
|  | *Rhinovirus A* | 17617 |
|  | *Human respiratory syncytial virus type A* | 10111 |
|  | *Moraxella catarrhalis* | 265 |
| 988570 | *Human Metapneumovirus (HMPV)* | 57 |
| 1023416 | *Acinetobacter baumannii* | 562 |
|  | *Streptococcus intermedius* | 439 |
|  | *Streptococcus constellatus* | 427 |
| 1028429 | *Streptococcus constellatus* | 3203 |
| 1028732 | *Human coronavirus OC43* | 55390 |
|  | *Moraxella catarrhalis* | 22075 |
|  | *Streptococcus intermedius* | 3277 |
|  | *Staphylococcus aureus* | 92 |
| 1028714 | *Human adenovirus group C* | 46794 |
|  | *Streptococcus pneumoniae* | 16437 |
|  | *Human respiratory syncytial virus type A* | 15459 |
|  | *Human coronavirus OC43* | 11920 |
| 1031125 | *Coxsackievirus type A10* | 52592 |
| 1004571 | *Rhinovirus type C* | 1339 |
|  | *Haemophilus influenzae* | 11520 |
|  | *Streptococcus intermedius* | 18819 |
| 1031050 | *Streptococcus anginosus* | 172 |
| 1031058 | *Human respiratory syncytial virus type A* | 76796 |
| 1031041 | *Mycoplasma pneumoniae* | 3442 |
|  | *Haemophilus influenzae* | 6251 |
| 1031054 | *Human Metapneumovirus (HMPV)* | 447 |
|  | *Streptococcus anginosus* | 2543 |
| 1031055 | *Bordetella pertussis* | 69851 |
|  | *Rhinovirus type C* | 22278 |
| 973091 | *Coxsackievirus type A5* | 36368 |
|  | *Haemophilus influenzae* | 1363 |
| 1031063 | *Streptococcus constellatus* | 14029 |
|  | *Streptococcus intermedius* | 11307 |
| 1031057 | *Human adenovirus group C* | 7559 |
|  | *Streptococcus pneumoniae* | 3684 |
|  | *Moraxella catarrhalis* | 1864 |
| 1031157 | *Human parainfluenza virus type 1* | 1816 |
|  | *Moraxella catarrhalis* | 39039 |
|  | *Streptococcus pneumoniae* | 157 |
|  | *Haemophilus influenzae* | 108 |
| 1031132 | *Streptococcus anginosus* | 211 |
| 1031168 | *Human bocavirus type 1* | 67485 |
|  | *Streptococcus intermedius* | 714 |
| 1014277 | *Human bocavirus type 1* | 50330 |
| 1030430 | *Human Metapneumovirus (HMPV)* | 23489 |
|  | *Bordetella pertussis* | 8570 |
|  | *Haemophilus influenzae* | 24525 |
|  | *Staphylococcus aureus* | 2208 |
|  | *Streptococcus intermedius* | 23560 |
| 1030447 | *Human bocavirus type 1* | 85126 |
|  | *Klebsiella pneumoniae* | 509 |
| 1030428 | *Human Metapneumovirus (HMPV)* | 16245 |
|  | *Bordetella pertussis* | 12933 |
|  | *Streptococcus pneumoniae* | 31705 |
|  | *Haemophilus influenzae* | 1209 |
| 1030484 | *Human bocavirus type 1* | 76523 |
|  | *Haemophilus influenzae* | 313 |
|  | *Streptococcus intermedius* | 1065 |
| 1030740 | *Human respiratory syncytial virus type A* | 897 |
|  | *Rhinovirus A* | 350 |
|  | *Bordetella pertussis* | 73 |
|  | *Streptococcus pneumoniae* | 330 |
|  | *Streptococcus anginosus* | 9443 |
| 1030615 | *Human coronavirus OC43* | 7449 |
|  | *Haemophilus influenzae* | 2781 |
|  | *Streptococcus constellatus* | 31529 |
| 1025973 | *Klebsiella pneumoniae* | 149 |
| 1030618 | *Streptococcus constellatus* | 12470 |
|  | *Streptococcus intermedius* | 9854 |
| 1030644 | *Coxsackievirus type A5* | 49881 |
|  | *Streptococcus pneumoniae* | 229 |
| 976749 | *Herpes simplex virus type 1* | 1413 |
|  | *Streptococcus pneumoniae* | 1778 |
|  | *Streptococcus constellatus* | 3015 |
| 1030651 | *Human Metapneumovirus (HMPV)* | 2958 |
|  | *Haemophilus influenzae* | 31618 |
|  | *Streptococcus pneumoniae* | 6391 |
| 1030733 | *Human bocavirus type 1* | 64979 |
|  | *Streptococcus pneumoniae* | 227 |
|  | *Streptococcus constellatus* | 1073 |
| 1030724 | *Enterovirus group A* | 35945 |
|  | *Streptococcus anginosus* | 1595 |
| 1030931 | *Human respiratory syncytial virus type A* | 52410 |
| 1030939 | *Human Metapneumovirus (HMPV)* | 58174 |
|  | *Moraxella catarrhalis* | 2282 |
| 1030918 | *Human parainfluenza virus type 3* | 858 |
|  | *Haemophilus influenzae* | 3646 |
| 1030913 | *Haemophilus influenzae* | 42110 |
|  | *Moraxella catarrhalis* | 348 |
|  | *Streptococcus pneumoniae* | 140 |
|  | *Streptococcus anginosus* | 5868 |
| 1031740 | *Bordetella pertussis* | 74385 |
|  | *Streptococcus pneumoniae* | 600 |
|  | *Haemophilus influenzae* | 115 |
| 1031726 | *Human adenovirus type 1* | 80107 |
|  | *Haemophilus influenzae* | 765 |
|  | *Streptococcus constellatus* | 2094 |
| 972682 | *Human Metapneumovirus (HMPV)* | 42137 |
|  | *Streptococcus constellatus* | 5367 |
| 1031894 | *Rhinovirus A* | 25382 |
|  | *Human parainfluenza virus type 3* | 124 |
|  | *Streptococcus pneumoniae* | 29414 |
|  | *Acinetobacter baumannii* | 1035 |
| 1031908 | *Streptococcus intermedius* | 3945 |
| 1031949 | *Human Metapneumovirus (HMPV)* | 23389 |
|  | *Rhinovirus type B* | 1817 |
|  | *Haemophilus influenzae* | 37560 |
|  | *Streptococcus pneumoniae* | 315 |
|  | *Moraxella catarrhalis* | 122 |
|  | *Streptococcus intermedius* | 5803 |
| 1032016 | *Human Metapneumovirus (HMPV)* | 19775 |
|  | *Streptococcus pneumoniae* | 5977 |
| 1027742 | *Human respiratory syncytial virus type A* | 69441 |
|  | *Streptococcus constellatus* | 16494 |
|  | *Streptococcus anginosus* | 8607 |
|  | *Streptococcus pneumoniae* | 3670 |
| 1027689 | *Streptococcus anginosus* | 17581 |
|  | *Human coronavirus OC43* | 8213 |
|  | *Streptococcus pneumoniae* | 6948 |
|  | *Haemophilus influenzae* | 53 |
| 1027678 | *Human respiratory syncytial virus type A* | 75304 |
|  | *Streptococcus anginosus* | 261 |
| 1027667 | *Streptococcus pneumoniae* | 90832 |
| 1027670 | *Human respiratory syncytial virus type A* | 79011 |
|  | *Streptococcus intermedius* | 2163 |
|  | *Moraxella catarrhalis* | 1597 |
| 1027860 | *Streptococcus intermedius* | 10001 |
| 1031437 | *Human Metapneumovirus (HMPV)* | 194 |
|  | *Streptococcus pneumoniae* | 95 |
| 1031439 | *Rhinovirus A* | 11 |
|  | *Streptococcus intermedius* | 624 |
| 1031463 | *Coxsackievirus type A10* | 35960 |
|  | *Streptococcus pneumoniae* | 1901 |
|  | *Moraxella catarrhalis* | 1792 |
|  | *Haemophilus influenzae* | 232 |
| 994161 | *Bordetella pertussis* | 3544 |
|  | *Streptococcus pneumoniae* | 30102 |
|  | *Haemophilus influenzae* | 2945 |
|  | *Moraxella catarrhalis* | 508 |
| 1031548 | *Human parainfluenza virus type 3* | 36201 |
|  | *Streptococcus intermedius* | 1739 |
| 1031555 | *Herpes simplex virus type 1* | 4224 |
|  | *Streptococcus pneumoniae* | 572 |
|  | *Streptococcus anginosus* | 22262 |
| 853311 | *Mycoplasma pneumoniae* | 4419 |
|  | *Rhinovirus type C* | 991 |
|  | *Streptococcus pneumoniae* | 80 |
| 1027751 | *Rhinovirus A* | 2983 |
|  | *Staphylococcus aureus* | 372 |
| 1027767 | *Human respiratory syncytial virus type A* | 3656 |
|  | *Streptococcus pneumoniae* | 52 |
| 1028214 | *Moraxella catarrhalis* | 453 |
| 1028261 | *Human respiratory syncytial virus type A* | 59359 |
| 1028889 | *Human respiratory syncytial virus type A* | 48464 |
|  | *Streptococcus anginosus* | 3287 |
|  | *Streptococcus intermedius* | 2522 |
| 1028807 | *Streptococcus intermedius* | 491 |
|  | *Streptococcus constellatus* | 478 |
| 1028825 | *Human respiratory syncytial virus type A* | 20988 |
| 879128 | *Rhinovirus* | 12632 |
| 1002057 | *Human coronavirus OC43* | 33380 |
|  | *Streptococcus pneumoniae* | 21584 |
|  | *Moraxella catarrhalis* | 19065 |
|  | *Staphylococcus aureus* | 28 |
| 1027184 | *Streptococcus intermedius* | 7502 |
|  | *Human respiratory syncytial virus type A* | 489 |
| 1027134 | *Human respiratory syncytial virus type A* | 23418 |
|  | *Streptococcus intermedius* | 325 |
| 1027213 | *Human respiratory syncytial virus type A* | 54207 |
|  | *Streptococcus intermedius* | 2207 |
|  | *Moraxella catarrhalis* | 680 |
| 1027215 | *Human respiratory syncytial virus type A* | 46530 |
|  | *Streptococcus pneumoniae* | 17950 |
|  | *Moraxella catarrhalis* | 11375 |
|  | *Rhinovirus type C* | 5846 |
|  | *Staphylococcus aureus* | 37 |
| 1028012 | *Streptococcus pneumoniae* | 117 |
|  | *Human coronavirus OC43* | 91 |
| 1028308 | *Human respiratory syncytial virus type B* | 84701 |
| 1028301 | *Human coronavirus OC43* | 74954 |
|  | *Moraxella catarrhalis* | 360 |
|  | *Streptococcus pneumoniae* | 278 |
| 1018870 | *Human respiratory syncytial virus type A* | 3767 |
|  | *Klebsiella pneumoniae* | 2021 |
|  | *Streptococcus intermedius* | 334 |
| 1028312 | *Human respiratory syncytial virus type A* | 71181 |
|  | *Human coronavirus OC43* | 155 |
| 1028511 | *Acinetobacter baumannii* | 283 |
|  | *Pseudomonas aeruginosa* | 12 |
| 1026295 | *Human Metapneumovirus (HMPV)* | 19989 |
|  | *Streptococcus constellatus* | 7504 |
| 1030834 | *Moraxella catarrhalis* | 202 |
|  | *Streptococcus constellatus* | 9897 |
|  | *Streptococcus intermedius* | 6736 |
| 1030986 | *Human parainfluenza virus type 3* | 67030 |
|  | *Haemophilus influenzae* | 5005 |
| 1030964 | *Human parainfluenza virus type 1* | 240 |
|  | *Streptococcus pneumoniae* | 273 |
|  | *Moraxella catarrhalis* | 149 |
|  | *Acinetobacter baumannii* | 17164 |
|  | *Streptococcus intermedius* | 6427 |
| 1031635 | *Human Metapneumovirus (HMPV)* | 2962 |
|  | *Haemophilus influenzae* | 3786 |
|  | *Streptococcus intermedius* | 7032 |
| 1031636 | *Coxsackievirus type A10* | 27457 |
|  | *Human adenovirus type 2* | 1420 |
|  | *Moraxella catarrhalis* | 3546 |
|  | *Streptococcus pneumoniae* | 375 |
| 957818 | *Haemophilus influenzae* | 204 |
|  | *Streptococcus intermedius* | 13503 |
| 1031666 | *Human parainfluenza virus type 4* | 8957 |
|  | *Staphylococcus aureus* | 2968 |
|  | *Streptococcus intermedius* | 36282 |
| 1031663 | *Human parainfluenza virus type 3* | 144 |
|  | *Streptococcus constellatus* | 319 |
| 1031719 | *Human bocavirus type 1* | 60870 |
|  | *Streptococcus constellatus* | 2579 |
| 1032413 | *Human parainfluenza virus type 3* | 62477 |
|  | *Streptococcus pneumoniae* | 5341 |
|  | *Staphylococcus aureus* | 1274 |
|  | *Streptococcus anginosus* | 3646 |
| 1032355 | *Human Metapneumovirus (HMPV)* | 7464 |
|  | *Streptococcus pneumoniae* | 223 |
|  | *Streptococcus intermedius* | 1291 |
| 1022428 | *Human Metapneumovirus (HMPV)* | 30953 |
|  | *Human respiratory syncytial virus type A* | 4935 |
|  | *Streptococcus pneumoniae* | 18313 |
|  | *Streptococcus constellatus* | 14823 |
| 919935 | *Human parainfluenza virus type 1* | 68010 |
|  | *Haemophilus influenzae* | 56 |
| 1032287 | *Human Metapneumovirus (HMPV)* | 79 |
|  | *Streptococcus pneumoniae* | 113 |
|  | *Streptococcus anginosus* | 2275 |
| 1027501 | *Streptococcus pneumoniae* | 44962 |
|  | *Human respiratory syncytial virus type A* | 40859 |
|  | *Staphylococcus aureus* | 5661 |
| 1027131 | *Human respiratory syncytial virus type A* | 28918 |
|  | *Streptococcus intermedius* | 16770 |
|  | *Herpes simplex virus type 1* | 1341 |
|  | *Human bocavirus type 1* | 35 |
| 1027468 | *Human respiratory syncytial virus type A* | 87211 |
|  | *Streptococcus intermedius* | 655 |
| 1027431 | *Human respiratory syncytial virus type A* | 2202 |
|  | *Streptococcus intermedius* | 1426 |
| 1027492 | *Streptococcus anginosus* | 2688 |
| 1027039 | *Human coronavirus OC43* | 50802 |
|  | *Moraxella catarrhalis* | 10964 |
| 1026997 | *Acinetobacter baumannii* | 2472 |
|  | *Rhinovirus A* | 123 |
| 1027392 | *Streptococcus intermedius* | 19821 |
|  | *Streptococcus pneumoniae* | 5960 |
|  | *Human respiratory syncytial virus type A* | 2447 |
| 1027388 | *Haemophilus influenzae* | 3905 |
|  | *Human respiratory syncytial virus type A* | 2855 |
|  | *Streptococcus pneumoniae* | 581 |
|  | *Staphylococcus aureus* | 515 |
|  | *Moraxella catarrhalis* | 482 |
| 1027471 | *Human respiratory syncytial virus type A* | 18222 |
|  | *Streptococcus intermedius* | 611 |
| 1029905 | *Human respiratory syncytial virus type B* | 38087 |
| 876702 | *Staphylococcus aureus* | 868 |
|  | *Streptococcus intermedius* | 14203 |
| 0997104 | *Rhinovirus A* | 14882 |
|  | *Haemophilus influenzae* | 1143 |
|  | *Streptococcus constellatus* | 988 |
| 1030153 | *Mycoplasma pneumoniae* | 19899 |
|  | *Streptococcus constellatus* | 1800 |
| 1030142 | *Human respiratory syncytial virus type B* | 1042 |
| 1030151 | *Human adenovirus type 1* | 84277 |
| 980753 | *Streptococcus constellatus* | 424 |
| 1001542 | *Coxsackievirus type A10* | 22787 |
|  | *Streptococcus anginosus* | 96 |
| 1031215 | *Rhinovirus A* | 5735 |
|  | *Haemophilus influenzae* | 2970 |
| 1031308 | *Human bocavirus type 1* | 62 |
|  | *Streptococcus constellatus* | 730 |
| 1031339 | *Human respiratory syncytial virus type A* | 16942 |
|  | *Streptococcus pneumoniae* | 534 |
| 1024047 | *Coxsackievirus type A10* | 15503 |
|  | *Moraxella catarrhalis* | 252 |
| 1034033 | *Human parainfluenza virus type 4* | 147 |
|  | *Haemophilus influenzae* | 1847 |
| 1034015 | *Bordetella pertussis* | 209 |
| 1034125 | *Human Metapneumovirus (HMPV)* | 695 |
|  | *Haemophilus influenzae* | 451 |
|  | *Streptococcus constellatus* | 1972 |
| 1034123 | *Human Metapneumovirus (HMPV)* | 23388 |
|  | *Streptococcus pneumoniae* | 7716 |
|  | *Streptococcus anginosus* | 6807 |
| 1023512 | *Rhinovirus type B* | 22207 |
|  | *Haemophilus influenzae* | 30489 |
|  | *Streptococcus pneumoniae* | 209 |
|  | *Moraxella catarrhalis* | 115 |
| 1034116 | *Coxsackievirus type A10* | 444 |
|  | *Human parainfluenza virus type 3* | 263 |
|  | *Human parainfluenza virus type 4* | 107 |
|  | *Streptococcus pneumoniae* | 50362 |
| 1034117 | *Human Metapneumovirus (HMPV)* | 28809 |
|  | *Haemophilus influenzae* | 20726 |
|  | *Streptococcus intermedius* | 1818 |
| 1034141 | *Human parainfluenza virus type 3* | 21858 |
|  | *Human bocavirus type 1* | 21480 |
|  | *Moraxella catarrhalis* | 3019 |
|  | *Streptococcus constellatus* | 5141 |
| 983914 | *Human Metapneumovirus (HMPV)* | 464 |
|  | *Klebsiella pneumoniae* | 386 |
|  | *Streptococcus constellatus* | 10585 |
| 1020419 | *Human parainfluenza virus type 3* | 5704 |
|  | *Staphylococcus aureus* | 71 |
| 1034139 | *Coxsackievirus type A10* | 15471 |
|  | *Human adenovirus type 1* | 4599 |
|  | *Moraxella catarrhalis* | 39098 |
|  | *Haemophilus influenzae* | 9819 |
|  | *Streptococcus pneumoniae* | 495 |
|  | *Streptococcus anginosus* | 891 |
| 1034191 | *Haemophilus influenzae* | 18721 |
|  | *Streptococcus pneumoniae* | 9505 |
|  | *Streptococcus intermedius* | 9124 |
| 1032691 | *Human Metapneumovirus (HMPV)* | 39999 |
|  | *Streptococcus pneumoniae* | 42898 |
| 1032707 | *Mycoplasma pneumoniae* | 16824 |
|  | *Streptococcus anginosus* | 3121 |
| 1032688 | *Rhinovirus A* | 14879 |
|  | *Haemophilus influenzae* | 445 |
|  | *Streptococcus intermedius* | 1297 |
| 1032716 | *Rhinovirus A* | 14958 |
|  | *Streptococcus pneumoniae* | 47825 |
|  | *Moraxella catarrhalis* | 29402 |
| 1032730 | *Coxsackievirus type A10* | 68369 |
|  | *Haemophilus influenzae* | 670 |
| 1032801 | *Human parainfluenza virus type 1* | 2142 |
|  | *Human respiratory syncytial virus type A* | 43 |
|  | *Staphylococcus aureus* | 145 |
|  | *Streptococcus constellatus* | 61948 |
| 1032810 | *Human bocavirus type 1* | 30837 |
|  | *Streptococcus intermedius* | 8351 |
| 1032812 | *Human Metapneumovirus (HMPV)* | 38433 |
|  | *Streptococcus pneumoniae* | 13024 |
| 1032813 | *Human Metapneumovirus (HMPV)* | 18640 |
|  | *Streptococcus pneumoniae* | 20383 |
|  | *Haemophilus influenzae* | 6087 |
|  | *Staphylococcus aureus* | 496 |
| 992734 | *Human parainfluenza virus type 3* | 37999 |
|  | *Streptococcus constellatus* | 14514 |
| 1032880 | *Human respiratory syncytial virus type A* | 57068 |
|  | *Moraxella catarrhalis* | 1426 |
|  | *Streptococcus intermedius* | 1079 |
| 1032817 | *Human parainfluenza virus type 3* | 26902 |
|  | *Rhinovirus A* | 13992 |
|  | *Human adenovirus type 5* | 148 |
|  | *Haemophilus influenzae* | 7728 |
|  | *Streptococcus pneumoniae* | 100 |
|  | *Streptococcus constellatus* | 8655 |
| 1020887 | *Human parainfluenza virus type 3* | 2101 |
|  | *Klebsiella pneumoniae* | 104 |
|  | *Streptococcus anginosus* | 1384 |
| 1034467 | *Human Metapneumovirus (HMPV)* | 30195 |
|  | *Streptococcus pneumoniae* | 879 |
|  | *Streptococcus constellatus* | 7985 |
| 1034477 | *Streptococcus intermedius* | 157 |
| 1034482 | *Human parainfluenza virus type 3* | 1370 |
| 1034475 | *Haemophilus influenzae* | 40 |
| 1034499 | *Rhinovirus A* | 27 |
|  | *Streptococcus pneumoniae* | 408 |
|  | *Streptococcus constellatus* | 228 |
| 1031893 | *Human parainfluenza virus type 3* | 33112 |
|  | *Human bocavirus type 1* | 23999 |
|  | *Haemophilus influenzae* | 22663 |
|  | *Staphylococcus aureus* | 81 |
|  | *Streptococcus intermedius* | 8421 |
| 1022877 | *Human parainfluenza virus type 3* | 670 |
|  | *Moraxella catarrhalis* | 560 |
|  | *Streptococcus pneumoniae* | 250 |
| 1031840 | *Mycoplasma pneumoniae* | 36521 |
|  | *Streptococcus constellatus* | 1136 |
| 1031814 | *Streptococcus anginosus* | 3405 |
| 1031896 | *Herpes simplex virus type 1* | 10540 |
|  | *Streptococcus pneumoniae* | 435 |
| 983245 | *Human parainfluenza virus type 3* | 2162 |
|  | *Haemophilus influenzae* | 77827 |
|  | *Streptococcus intermedius* | 1215 |
| 1034582 | *Rhinovirus A* | 6277 |
|  | *Streptococcus pneumoniae* | 7041 |
|  | *Haemophilus influenzae* | 516 |
|  | *Streptococcus anginosus* | 924 |
| 1034603 | *Human Metapneumovirus (HMPV)* | 4343 |
|  | *Rhinovirus type C* | 1027 |
|  | *Haemophilus influenzae* | 33614 |
| 1034559 | *Human parainfluenza virus type 3* | 34703 |
|  | *Human adenovirus type 1* | 103 |
|  | *Streptococcus constellatus* | 4684 |
| 1034680 | *Haemophilus influenzae* | 12200 |
|  | *Staphylococcus aureus* | 570 |
|  | *Acinetobacter baumannii* | 1881 |
| 1034691 | *Human Metapneumovirus (HMPV)* | 3171 |
|  | *Streptococcus intermedius* | 2336 |
| 1034694 | *Human bocavirus type 1* | 2848 |
|  | *Streptococcus intermedius* | 11158 |
| 1034771 | *Human Metapneumovirus (HMPV)* | 35282 |
|  | *Moraxella catarrhalis* | 23164 |
|  | *Streptococcus pneumoniae* | 14972 |
|  | *Haemophilus influenzae* | 869 |
| 1034703 | *Human Metapneumovirus (HMPV)* | 39390 |
|  | *Haemophilus influenzae* | 1074 |
|  | *Klebsiella pneumoniae* | 73 |
|  | *Streptococcus anginosus* | 2068 |
| 1034699 | *Human Metapneumovirus (HMPV)* | 5873 |
|  | *Streptococcus intermedius* | 1230 |
| 1034700 | *Human respiratory syncytial virus type A* | 36857 |
|  | *Coxsackievirus type A10* | 201 |
|  | *Streptococcus intermedius* | 1587 |
| 1033325 | *Human parainfluenza virus type 3* | 45899 |
|  | *Streptococcus anginosus* | 10893 |
| 1033329 | *Human parainfluenza virus type 3* | 7631 |
|  | *Haemophilus influenzae* | 578 |
|  | *Streptococcus intermedius* | 1191 |
| 1033337 | *Human bocavirus type 1* | 74997 |
|  | *Acinetobacter baumannii* | 317 |
|  | *Streptococcus anginosus* | 1013 |
| 1033348 | *Klebsiella pneumoniae* | 171 |
|  | *Streptococcus constellatus* | 2029 |
| 1033347 | *Human parainfluenza virus type 1* | 63492 |
|  | *Streptococcus constellatus* | 1963 |
| 1033338 | *Coxsackievirus type A5* | 72641 |
|  | *Haemophilus influenzae* | 90 |
| 984935 | *Human parainfluenza virus type 4* | 154 |
|  | *Coxsackievirus type A10* | 33 |
|  | *Streptococcus intermedius* | 183 |
| 1033517 | *Human respiratory syncytial virus type A* | 60130 |
| 1033505 | *Human bocavirus type 1* | 32054 |
|  | *Human parainfluenza virus type 3* | 31839 |
|  | *Streptococcus pneumoniae* | 26845 |
| 1033579 | *Human parainfluenza virus type 3* | 40568 |
| 1033513 | *Human Metapneumovirus (HMPV)* | 35862 |
|  | *Human parainfluenza virus type 3* | 344 |
|  | *Haemophilus influenzae* | 22437 |
|  | *Streptococcus pneumoniae* | 1841 |
| 1033509 | *Haemophilus influenzae* | 1065 |
|  | *Streptococcus anginosus* | 7797 |
| 1024515 | *Human Metapneumovirus (HMPV)* | 33556 |
|  | *Rhinovirus A* | 3997 |
|  | *Streptococcus pneumoniae* | 28185 |
| 1034198 | *Streptococcus pneumoniae* | 1882 |
| 1034269 | *Human parainfluenza virus type 4* | 380 |
|  | *Rhinovirus A* | 28 |
|  | *Streptococcus pneumoniae* | 7142 |
|  | *Streptococcus intermedius* | 2579 |
| 1034211 | *Human bocavirus type 1* | 70068 |
|  | *Streptococcus anginosus* | 627 |
| 1022201 | *Human adenovirus type 2* | 84738 |
|  | *Moraxella catarrhalis* | 765 |
|  | *Haemophilus influenzae* | 148 |
|  | *Streptococcus pneumoniae* | 133 |
| 1034206 | *Human Metapneumovirus (HMPV)* | 24211 |
|  | *Streptococcus pneumoniae* | 5863 |
|  | *Moraxella catarrhalis* | 356 |
| 1034200 | *Bordetella pertussis* | 66381 |
|  | *Rhinovirus A* | 525 |
|  | *Haemophilus influenzae* | 2702 |
|  | *Streptococcus pneumoniae* | 2183 |
|  | *Moraxella catarrhalis* | 565 |
|  | *Staphylococcus aureus* | 501 |
| 1019640 | *Coxsackievirus type A6* | 28735 |
| 1034302 | *Human adenovirus type 2* | 778 |
|  | *Streptococcus anginosus* | 319 |
| 1034252 | *Rhinovirus A* | 500 |
|  | *Haemophilus influenzae* | 107 |
|  | *Streptococcus intermedius* | 325 |
| 1034298 | *Streptococcus pyogenes* | 259 |
| 912993 | *Haemophilus influenzae* | 11418 |
|  | *Streptococcus constellatus* | 4730 |
| 1034289 | *Coxsackievirus type A6* | 21945 |
|  | *Streptococcus pneumoniae* | 9366 |
|  | *Haemophilus influenzae* | 3389 |
| 1033209 | *Human Metapneumovirus (HMPV)* | 17277 |
|  | *Streptococcus anginosus* | 17830 |
| 1033233 | *Human parainfluenza virus type 3* | 34787 |
|  | *Haemophilus influenzae* | 7008 |
|  | *Streptococcus pneumoniae* | 1203 |
| 1033217 | *Human Metapneumovirus (HMPV)* | 20158 |
|  | *Human coronavirus HKU1* | 461 |
|  | *Haemophilus influenzae* | 3165 |
|  | *Streptococcus pneumoniae* | 411 |
|  | *Streptococcus intermedius* | 2948 |
| 1033426 | *Human adenovirus group C* | 77038 |
| 1033265 | *Rhinovirus A* | 16334 |
|  | *Human adenovirus type 1* | 61 |
|  | *Haemophilus influenzae* | 36783 |
|  | *Streptococcus anginosus* | 18102 |
| 1033418 | *Human parainfluenza virus type 3* | 8119 |
|  | *Rhinovirus A* | 4251 |
|  | *Streptococcus pneumoniae* | 19395 |
|  | *Moraxella catarrhalis* | 148 |
|  | *Streptococcus anginosus* | 49142 |
| 1033484 | *Mycoplasma pneumoniae* | 43063 |
|  | *Haemophilus influenzae* | 129 |
| 1026668 | *Coxsackievirus type A6* | 39885 |
|  | *Klebsiella pneumoniae* | 118 |
|  | *Streptococcus anginosus* | 1532 |
| 1033434 | *Human bocavirus type 1* | 334 |
|  | *Human parainfluenza virus type 3* | 118 |
|  | *Klebsiella pneumoniae* | 850 |
|  | *Streptococcus pneumoniae* | 529 |
| 1033440 | *Human parainfluenza virus type 3* | 7828 |
|  | *Streptococcus pneumoniae* | 124 |
|  | *Klebsiella pneumoniae* | 96 |
|  | *Acinetobacter baumannii* | 2434 |
| 1033504 | *Streptococcus intermedius* | 7158 |
| 1033824 | *Coxsackievirus type A6* | 60268 |
|  | *Human parainfluenza virus type 4* | 34 |
|  | *Acinetobacter baumannii* | 1124 |
| 1033835 | *Human Metapneumovirus (HMPV)* | 608 |
| 1033825 | *Coxsackievirus type A10* | 38025 |
|  | *Human parainfluenza virus type 3* | 30 |
|  | *Haemophilus influenzae* | 4279 |
| 902408 | *Coxsackievirus type A6* | 38541 |
|  | *Rhinovirus A* | 1424 |
|  | *Haemophilus influenzae* | 3087 |
| 1033820 | *Human bocavirus type 1* | 41609 |
|  | *Streptococcus intermedius* | 2597 |
| 1033846 | *Rhinovirus type C* | 27379 |
|  | *Human Metapneumovirus (HMPV)* | 566 |
|  | *Streptococcus pneumoniae* | 35583 |
|  | *Moraxella catarrhalis* | 5265 |
|  | *Staphylococcus aureus* | 167 |
| 1033849 | *Mycoplasma pneumoniae* | 7892 |
|  | *Rhinovirus A* | 293 |
| 1033833 | *Rhinovirus A* | 13960 |
|  | *Human parainfluenza virus type 3* | 4842 |
|  | *Staphylococcus aureus* | 127 |
| 1033924 | *Human Metapneumovirus (HMPV)* | 41056 |
|  | *Streptococcus pneumoniae* | 1021 |
|  | *Haemophilus influenzae* | 420 |
| 1033840 | *Streptococcus intermedius* | 412 |
| 1033932 | *Human Metapneumovirus (HMPV)* | 24246 |
|  | *Streptococcus pneumoniae* | 60012 |
| 1033946 | *Human bocavirus type 1* | 3368 |
| 1020148 | *Coxsackievirus type A6* | 35023 |
|  | *Haemophilus influenzae* | 205 |
| 1034011 | *Human parainfluenza virus type 3* | 10604 |
|  | *Haemophilus influenzae* | 231 |
|  | *Streptococcus intermedius* | 4158 |
| 1033662 | *Coxsackievirus type A10* | 24 |
|  | *Streptococcus intermedius* | 242 |
| 1033664 | *Human parainfluenza virus type 1* | 23693 |
| 1033660 | *Human bocavirus type 1* | 32541 |
| 1033668 | *Human Metapneumovirus (HMPV)* | 78527 |
|  | *Moraxella catarrhalis* | 1560 |
| 1033667 | *Human Metapneumovirus (HMPV)* | 53650 |
|  | *Human parainfluenza virus type 1* | 15286 |
|  | *Streptococcus pneumoniae* | 13132 |
| 1033738 | *Human Metapneumovirus (HMPV)* | 55101 |
|  | *Human bocavirus type 1* | 1935 |
|  | *Coxsackievirus type A10* | 298 |
| 981329 | *Staphylococcus aureus* | 91 |
| 1033742 | *Human parainfluenza virus type 4* | 2254 |
|  | *Haemophilus influenzae* | 32486 |
|  | *Moraxella catarrhalis* | 550 |
|  | *Staphylococcus aureus* | 169 |
|  | *Streptococcus pneumoniae* | 83 |
|  | *Streptococcus intermedius* | 1966 |
| 1033744 | *Human Metapneumovirus (HMPV)* | 4855 |
|  | *Haemophilus influenzae* | 87 |
|  | *Streptococcus intermedius* | 5155 |
| 1033746 | *Human parainfluenza virus type 3* | 61 |
|  | *Haemophilus influenzae* | 11112 |
|  | *Streptococcus anginosus* | 846 |
| 1033760 | *Human Metapneumovirus (HMPV)* | 381 |
|  | *Streptococcus pneumoniae* | 803 |
|  | *Streptococcus intermedius* | 1602 |
| 1033759 | *Human parainfluenza virus type 3* | 47483 |
|  | *Haemophilus influenzae* | 17657 |
|  | *Streptococcus intermedius* | 1520 |
| 1032423 | *Human respiratory syncytial virus type A* | 85551 |
|  | *Moraxella catarrhalis* | 3882 |
| 1032418 | *Bordetella pertussis* | 76036 |
|  | *Rhinovirus A* | 3995 |
| 988128 | *Human bocavirus type 1* | 11089 |
|  | *Streptococcus pneumoniae* | 8090 |
| 1032422 | *Human Metapneumovirus (HMPV)* | 17182 |
|  | *Haemophilus influenzae* | 73 |
|  | *Streptococcus constellatus* | 4629 |
| 1031251 | *Human coronavirus OC43* | 77425 |
|  | *Staphylococcus aureus* | 165 |
| 989387 | *Rhinovirus A* | 391 |
|  | *Streptococcus pneumoniae* | 270 |
|  | *Staphylococcus aureus* | 52 |
|  | *Streptococcus intermedius* | 11128 |
| 1032586 | *Rhinovirus A* | 26 |
|  | *Streptococcus anginosus* | 10524 |
| 1032589 | *Mycoplasma pneumoniae* | 14090 |
|  | *Haemophilus influenzae* | 409 |
|  | *Streptococcus constellatus* | 3301 |
|  | *Streptococcus anginosus* | 2032 |
| 1032593 | *Human respiratory syncytial virus type A* | 13731 |
| 1032607 | *Human Metapneumovirus (HMPV)* | 6741 |
| 1032686 | *Haemophilus influenzae* | 266 |
|  | *Streptococcus intermedius* | 2877 |
| 1032583 | *Human adenovirus type 2* | 85578 |
| 1032689 | *Human parainfluenza virus type 3* | 37704 |
|  | *Rhinovirus A* | 25604 |
|  | *Moraxella catarrhalis* | 3606 |
|  | *Acinetobacter baumannii* | 1977 |
| 1032696 | *Staphylococcus aureus* | 3342 |
|  | *Haemophilus influenzae* | 2613 |
|  | *Streptococcus intermedius* | 1153 |
| 1032695 | *Rhinovirus A* | 394 |
|  | *Streptococcus pneumoniae* | 410 |
|  | *Streptococcus intermedius* | 2742 |
| 922029 | *Streptococcus pneumoniae* | 241 |
|  | *Streptococcus intermedius* | 3450 |
| 1033065 | *Human Metapneumovirus (HMPV)* | 4538 |
|  | *Human adenovirus type 1* | 81 |
|  | *Streptococcus intermedius* | 8907 |
| 1033067 | *Human Metapneumovirus (HMPV)* | 42929 |
|  | *Coxsackievirus type A10* | 4144 |
|  | *Human parainfluenza virus type 1* | 502 |
|  | *Streptococcus anginosus* | 21073 |
| 1033069 | *Human Metapneumovirus (HMPV)* | 8099 |
|  | *Human parainfluenza virus type 1* | 381 |
|  | *Haemophilus influenzae* | 138 |
|  | *Streptococcus intermedius* | 2044 |
| 1033076 | *Human respiratory syncytial virus type A* | 17186 |
|  | *Haemophilus influenzae* | 64752 |
|  | *Staphylococcus aureus* | 271 |
| 1032889 | *Human parainfluenza virus type 1* | 20633 |
|  | *Streptococcus intermedius* | 11715 |
| 1032859 | *Rhinovirus type B* | 16310 |
|  | *Streptococcus pyogenes* | 1054 |
|  | *Enterovirus group B* | 65 |
|  | *Haemophilus influenzae* | 17415 |
|  | *Streptococcus pneumoniae* | 7851 |
| 1032893 | *Human parainfluenza virus type 1* | 19575 |
|  | *Moraxella catarrhalis* | 257 |
|  | *Streptococcus intermedius* | 1011 |
| 956238 | *Human parainfluenza virus type 3* | 7490 |
|  | *Streptococcus intermedius* | 3261 |
| 1032956 | *Mycoplasma pneumoniae* | 49746 |
|  | *Human parainfluenza virus type 1* | 1601 |
|  | *Rhinovirus A* | 932 |
| 1032993 | *Moraxella catarrhalis* | 7454 |
|  | *Haemophilus influenzae* | 426 |
|  | *Streptococcus intermedius* | 6741 |
| 1032991 | *Bordetella pertussis* | 14066 |
|  | *Human Metapneumovirus (HMPV)* | 3970 |
|  | *Acinetobacter baumannii* | 945 |
| 1033001 | *Human Metapneumovirus (HMPV)* | 42731 |
|  | *Haemophilus influenzae* | 1378 |
|  | *Streptococcus constellatus* | 505 |
| 1032981 | *Human bocavirus type 1* | 43230 |
|  | *Haemophilus influenzae* | 214 |
|  | *Streptococcus anginosus* | 4686 |
| 948914 | *Coxsackievirus type A10* | 30503 |
|  | *Human parainfluenza virus type 3* | 5552 |
|  | *Human bocavirus type 1* | 133 |
|  | *Streptococcus agalactiae* | 40 |
| 1033589 | *Streptococcus pneumoniae* | 632 |
|  | *Streptococcus intermedius* | 7344 |
| 1033594 | *Rhinovirus A* | 18866 |
|  | *Streptococcus pneumoniae* | 2380 |
| 1033651 | *Rhinovirus A* | 35716 |
|  | *Coxsackievirus type A10* | 330 |
| 1033645 | *Human parainfluenza virus type 1* | 3087 |
|  | *Rhinovirus A* | 2292 |
|  | *Streptococcus pneumoniae* | 3645 |
|  | *Moraxella catarrhalis* | 141 |
|  | *Streptococcus constellatus* | 8100 |
| 1011611 | *Coxsackievirus type A6* | 21036 |
|  | *Human respiratory syncytial virus type A* | 492 |
|  | *Streptococcus pneumoniae* | 41942 |
| 1033574 | *Mycoplasma pneumoniae* | 5445 |
|  | *Streptococcus anginosus* | 721 |
| 986464 | *Human Metapneumovirus (HMPV)* | 5755 |
| 1032130 | *Human parainfluenza virus type 1* | 38673 |
|  | *Streptococcus constellatus* | 14454 |
| 1032145 | *Human bocavirus type 1* | 25297 |
|  | *Human parainfluenza virus type 4* | 159 |
|  | *Streptococcus pneumoniae* | 6849 |
|  | *Staphylococcus aureus* | 30 |
|  | *Streptococcus intermedius* | 9157 |
| 1032189 | *Human bocavirus type 1* | 26843 |
|  | *Streptococcus intermedius* | 1094 |
| 1032162 | *Human adenovirus type 2* | 103 |
|  | *Haemophilus influenzae* | 24 |
| 1018783 | *Coxsackievirus type A10* | 44083 |
| 1032250 | *Human respiratory syncytial virus type B* | 18716 |
|  | *Human bocavirus type 1* | 5616 |
|  | *Streptococcus pneumoniae* | 1305 |
|  | *Streptococcus intermedius* | 7183 |
| 1032128 | *Coxsackievirus type A10* | 930 |
|  | *Human respiratory syncytial virus type A* | 323 |
|  | *Moraxella catarrhalis* | 73 |
|  | *Staphylococcus aureus* | 69 |
| 1032244 | *Human parainfluenza virus type 3* | 1284 |
|  | *Haemophilus influenzae* | 238 |
|  | *Streptococcus intermedius* | 13208 |
| 972926 | *Human Metapneumovirus (HMPV)* | 2393 |
|  | *Streptococcus agalactiae* | 55 |
|  | *Streptococcus pneumoniae* | 150 |
|  | *Streptococcus intermedius* | 13988 |
| 1032050 | *Moraxella catarrhalis* | 238 |
|  | *Streptococcus anginosus* | 13719 |
| 1032047 | *Rhinovirus A* | 6644 |
|  | *Streptococcus pneumoniae* | 41056 |
|  | *Moraxella catarrhalis* | 4909 |
|  | *Haemophilus influenzae* | 2246 |
| 1032076 | *Human bocavirus type 1* | 21912 |
|  | *Streptococcus pneumoniae* | 3216 |
| 939842 | *Coxsackievirus type A6* | 36936 |
|  | *Human Metapneumovirus (HMPV)* | 12982 |
| 1032259 | *Human adenovirus group C* | 39209 |
|  | *Human parainfluenza virus type 4* | 5542 |
|  | *Haemophilus influenzae* | 12775 |
|  | *Streptococcus pneumoniae* | 9231 |
| 1032264 | *Human Metapneumovirus (HMPV)* | 1312 |
|  | *Haemophilus influenzae* | 2604 |
|  | *Streptococcus anginosus* | 5728 |
| 1032327 | *Human adenovirus group C* | 71557 |
|  | *Haemophilus influenzae* | 1810 |
| 885247 | *Streptococcus anginosus* | 16547 |
| 1032339 | *Streptococcus pneumoniae* | 383 |
|  | *Streptococcus intermedius* | 2482 |
| 1032504 | *Human bocavirus type 1* | 18355 |
| 1025778 | *Staphylococcus aureus* | 90 |
|  | *Streptococcus intermedius* | 2912 |
| 987085 | *Human Metapneumovirus (HMPV)* | 1085 |
| 980802 | *Human Metapneumovirus (HMPV)* | 9254 |
|  | *Human parainfluenza virus type 4* | 76 |
|  | *Streptococcus pneumoniae* | 208 |
| 953019 | *Coxsackievirus type A10* | 46820 |
|  | *Haemophilus influenzae* | 902 |
|  | *Klebsiella pneumoniae* | 373 |
| 1033173 | *Human parainfluenza virus type 3* | 42750 |
|  | *Bordetella pertussis* | 39640 |
|  | *Human coronavirus OC43* | 7037 |
| 1033208 | *Streptococcus pneumoniae* | 387 |
|  | *Streptococcus intermedius* | 14339 |
| 1024512 | *Human parainfluenza virus type 3* | 84149 |
| 1033139 | *Human parainfluenza virus type 3* | 4720 |
|  | *Rhinovirus A* | 88 |
|  | *Streptococcus pneumoniae* | 4634 |
|  | *Streptococcus anginosus* | 3092 |
| 1033117 | *Streptococcus intermedius* | 4175 |
| 1030557 | *Rhinovirus A* | 9253 |
|  | *Haemophilus influenzae* | 42 |
|  | *Streptococcus intermedius* | 654 |
| 1030561 | *Human bocavirus type 1* | 36082 |
|  | *Haemophilus influenzae* | 81 |
|  | *Streptococcus anginosus* | 2737 |
| 1039341 | *Cytomegalovirus* | 14228 |
| 1037254 | *Epstein-Barr virus* | 91 |
| 1034885 | *Human parainfluenza virus type 3* | 90 |
| 1035167 | *Human Metapneumovirus (HMPV)* | 13831 |
|  | *Streptococcus pneumoniae* | 47681 |
|  | *Moraxella catarrhalis* | 92 |
|  | *Haemophilus influenzae* | 34 |
|  | *Acinetobacter baumannii* | 218 |
| 1034872 | *Mycoplasma pneumoniae* | 26272 |
| 1035087 | *Human Metapneumovirus (HMPV)* | 89 |
|  | *Streptococcus pneumoniae* | 17775 |
|  | *Moraxella catarrhalis* | 30 |
| 1035548 | *Human Metapneumovirus (HMPV)* | 37 |
|  | *Haemophilus influenzae* | 3408 |
|  | *Streptococcus anginosus* | 2127 |
| 897777 | *Human Metapneumovirus (HMPV)* | 20431 |
|  | *Streptococcus intermedius* | 300 |
| 1036450 | *Staphylococcus aureus* | 35 |
|  | *Streptococcus anginosus* | 146 |
| 1006177 | *Human parainfluenza virus type 4* | 9854 |
|  | *Haemophilus influenzae* | 24900 |
| 1036547 | *SARS-CoV-2 omicron XBB* | 20313 |
|  | *Mycoplasma pneumoniae* | 4963 |
| 1035076 | *Mycoplasma pneumoniae* | 3670 |
|  | *Streptococcus constellatus* | 1623 |
| 1024919 | *Human Metapneumovirus (HMPV)* | 37656 |
|  | *Streptococcus intermedius* | 43 |
| 1034957 | *SARS-CoV-2 omicron XBB* | 4822 |
|  | *Bordetella pertussis* | 29 |
|  | *Haemophilus influenzae* | 143 |
| 1035062 | *Klebsiella pneumoniae* | 54 |
|  | *Streptococcus intermedius* | 136 |
| 856337 | *Mycoplasma pneumoniae* | 12769 |
|  | *Streptococcus pneumoniae* | 30 |
|  | *Streptococcus intermedius* | 398 |
| 1035495 | *Human Metapneumovirus (HMPV)* | 8975 |
|  | *Streptococcus pneumoniae* | 13142 |
|  | *Streptococcus intermedius* | 15141 |
| 1035172 | *Human respiratory syncytial virus type A* | 38307 |
|  | *Human Metapneumovirus (HMPV)* | 2667 |
|  | *Haemophilus influenzae* | 1342 |
|  | *Staphylococcus aureus* | 102 |
|  | *Streptococcus anginosus* | 6195 |
| 1021413 | *Human respiratory syncytial virus type A* | 32114 |
|  | *Klebsiella pneumoniae* | 638 |
|  | *Moraxella catarrhalis* | 33 |
|  | *Streptococcus intermedius* | 385 |
|  | *Acinetobacter baumannii* | 294 |
| 1035662 | *Human parainfluenza virus type 3* | 98 |
|  | *Streptococcus pneumoniae* | 34582 |
|  | *Staphylococcus aureus* | 63 |
| 1028456 | *Streptococcus pyogenes* | 12613 |
|  | *Streptococcus intermedius* | 5848 |
| 1035574 | *Human Metapneumovirus (HMPV)* | 19766 |
|  | *Staphylococcus aureus* | 111 |
| 1034828 | *Bordetella pertussis* | 42370 |
|  | *Rhinovirus A* | 5524 |
|  | *Haemophilus influenzae* | 2639 |
|  | *Streptococcus constellatus* | 3395 |
| 1024043 | *Human parainfluenza virus type 3* | 60261 |
|  | *Human respiratory syncytial virus type B* | 21 |
|  | *Acinetobacter baumannii* | 76 |
|  | *Streptococcus constellatus* | 773 |
| 1035482 | *Haemophilus influenzae* | 1192 |
|  | *Staphylococcus aureus* | 183 |
|  | *Streptococcus constellatus* | 8627 |
| 1035061 | *Herpes simplex virus type 1* | 1285 |
|  | *Klebsiella pneumoniae* | 92 |
|  | *Acinetobacter baumannii* | 1145 |
| 1035405 | *Human Metapneumovirus (HMPV)* | 6463 |
|  | *Streptococcus anginosus* | 96 |
| 1036108 | *Haemophilus influenzae* | 190 |
|  | *Streptococcus anginosus* | 913 |
| 1035047 | *Enterovirus group A* | 28279 |
|  | *Human bocavirus type 1* | 61 |
|  | *Human parainfluenza virus type 1* | 22 |
|  | *Klebsiella pneumoniae* | 924 |
|  | *Pseudomonas aeruginosa* | 50 |
|  | *Acinetobacter baumannii* | 1097 |
| 1036469 | *Human Metapneumovirus (HMPV)* | 2173 |
|  | *Haemophilus influenzae* | 32432 |
|  | *Streptococcus pneumoniae* | 18315 |
| 1036239 | *Rhinovirus A* | 123 |
|  | *Haemophilus influenzae* | 29680 |
|  | *Streptococcus constellatus* | 17858 |
| 1035063 | *Streptococcus pneumoniae* | 1930 |
|  | *Haemophilus influenzae* | 950 |
| 1035192 | *Human parainfluenza virus type 3* | 4713 |
|  | *Streptococcus intermedius* | 1903 |
| 1034789 | *Human parainfluenza virus type 1* | 1500 |
|  | *Human parainfluenza virus type 4* | 87 |
|  | *Klebsiella pneumoniae* | 82 |
|  | *Streptococcus intermedius* | 603 |
| 1036226 | *Human parainfluenza virus type 3* | 33862 |
|  | *Haemophilus influenzae* | 151 |
| 1035159 | *Human parainfluenza virus type 3* | 33745 |
|  | *Human Metapneumovirus (HMPV)* | 727 |
|  | *Streptococcus pneumoniae* | 22 |
|  | *Streptococcus anginosus* | 1328 |
| 901984 | *Mycoplasma pneumoniae* | 37943 |
|  | *Streptococcus pneumoniae* | 14424 |
|  | *Klebsiella pneumoniae* | 72 |
| 1035375 | *Rhinovirus A* | 32384 |
|  | *Herpes simplex virus type 1* | 5849 |
|  | *Moraxella catarrhalis* | 14124 |
|  | *Streptococcus pneumoniae* | 123 |
|  | *Acinetobacter baumannii* | 64 |
| 1035460 | *SARS-CoV-2 omicron XBB* | 15091 |
|  | *Moraxella catarrhalis* | 1875 |
|  | *Staphylococcus aureus* | 22 |
|  | *Streptococcus intermedius* | 45 |
| 1036598 | *Mycoplasma pneumoniae* | 226 |
| 1035497 | *Influenza A virus H3N2* | 321 |
|  | *Streptococcus pneumoniae* | 11119 |
|  | *Streptococcus intermedius* | 2260 |
| 1034785 | *Streptococcus constellatus* | 29 |
| 1035371 | *Human respiratory syncytial virus type B* | 33303 |
|  | *Human bocavirus type 1* | 29306 |
|  | *Human parainfluenza virus type 3* | 25512 |
|  | *Herpes simplex virus type 1* | 24 |
|  | *Acinetobacter baumannii* | 105 |
|  | *Streptococcus anginosus* | 38 |
| 1034979 | *Human Metapneumovirus (HMPV)* | 53012 |
|  | *Rhinovirus A* | 862 |
|  | *Human respiratory syncytial virus type A* | 33 |
|  | *Human parainfluenza virus type 3* | 22 |
|  | *Moraxella catarrhalis* | 2150 |
| 1036325 | *Human parainfluenza virus type 1* | 201 |
|  | *Streptococcus anginosus* | 50 |
| 1035403 | *Human bocavirus type 1* | 37 |
|  | *Haemophilus influenzae* | 57 |
|  | *Streptococcus anginosus* | 2628 |
| 1035479 | *Rhinovirus A* | 167 |
|  | *Streptococcus anginosus* | 2111 |
| 1026045 | *Streptococcus intermedius* | 66 |
| 1034961 | *Human Metapneumovirus (HMPV)* | 14235 |
|  | *Streptococcus intermedius* | 224 |
| 1035151 | *Mycoplasma pneumoniae* | 3419 |
|  | *Haemophilus influenzae* | 36 |
|  | *Staphylococcus aureus* | 26 |
|  | *Streptococcus anginosus* | 456 |
|  | *Acinetobacter baumannii* | 134 |
| 1035387 | *Human parainfluenza virus type 3* | 3987 |
|  | *Staphylococcus aureus* | 82 |
| 1027508 | *Human parainfluenza virus type 3* | 6069 |
|  | *Haemophilus influenzae* | 1137 |
|  | *Streptococcus anginosus* | 412 |
| 1005156 | *Mycoplasma pneumoniae* | 32165 |
|  | *Rhinovirus A* | 8411 |
|  | *Moraxella catarrhalis* | 15641 |
|  | *Haemophilus influenzae* | 590 |
|  | *Streptococcus pneumoniae* | 501 |
|  | *Streptococcus constellatus* | 1481 |
| 884098 | *Human Metapneumovirus (HMPV)* | 5600 |
|  | *Staphylococcus aureus* | 16 |
|  | *Streptococcus intermedius* | 801 |
| 1036205 | *Mycoplasma pneumoniae* | 34037 |
|  | *Streptococcus constellatus* | 69 |
| 1035165 | *Mycoplasma pneumoniae* | 30325 |
|  | *Haemophilus influenzae* | 423 |
| 1035904 | *Coxsackievirus type A10* | 24364 |
|  | *Streptococcus intermedius* | 191 |
| 1035292 | *Bordetella pertussis* | 8159 |
|  | *Streptococcus constellatus* | 169 |
| 1035378 | *Human adenovirus type 3* | 83914 |
|  | *Klebsiella pneumoniae* | 160 |
|  | *Haemophilus influenzae* | 18 |
|  | *Streptococcus anginosus* | 1013 |
|  | *Acinetobacter baumannii* | 57 |
| 1036491 | *Human bocavirus type 1* | 27533 |
|  | *Staphylococcus aureus* | 480 |
|  | *Streptococcus intermedius* | 10129 |
| 1036617 | *Human parainfluenza virus type 3* | 2710 |
|  | *Streptococcus pyogenes* | 435 |
|  | *Haemophilus influenzae* | 6352 |
| 1035468 | *Human Metapneumovirus (HMPV)* | 295 |
| 982003 | *Mycoplasma pneumoniae* | 31195 |
|  | *Haemophilus influenzae* | 149 |
| 1035567 | *Rhinovirus A* | 30504 |
|  | *Human parainfluenza virus type 3* | 34 |
|  | *Haemophilus influenzae* | 13400 |
|  | *Streptococcus intermedius* | 165 |
| 1035804 | *Streptococcus intermedius* | 63 |
| 953013 | *Mycoplasma pneumoniae* | 5791 |
| 1007704 | *Bordetella pertussis* | 62916 |
|  | *Rhinovirus A* | 21999 |
|  | *Streptococcus intermedius* | 138 |
| 1036632 | *Human parainfluenza virus type 1* | 36311 |
|  | *Streptococcus pneumoniae* | 28414 |
|  | *Streptococcus intermedius* | 18 |
| 1036118 | *Mycoplasma pneumoniae* | 15531 |
|  | *Staphylococcus aureus* | 12 |
| 1038152 | *Influenza A virus H3N2* | 1654 |
|  | *Herpes simplex virus type 1* | 74 |
|  | *Haemophilus influenzae* | 73271 |
|  | *Streptococcus intermedius* | 6198 |
| 1036889 | *Human adenovirus type 3* | 56176 |
|  | *Herpes simplex virus type 1* | 6 |
|  | *Haemophilus influenzae* | 188 |
| 1037839 | *Human respiratory syncytial virus type A* | 20903 |
| 1038890 | *Staphylococcus aureus* | 12585 |
|  | *Moraxella catarrhalis* | 468 |
|  | *Streptococcus intermedius* | 11775 |
| 1038929 | *Mycoplasma pneumoniae* | 55043 |
|  | *Haemophilus influenzae* | 233 |
|  | *Streptococcus anginosus* | 133 |
| 1035769 | *Human Metapneumovirus (HMPV)* | 3481 |
|  | *Coxsackievirus type A6* | 28 |
|  | *Haemophilus influenzae* | 31 |
| 1038931 | *Human respiratory syncytial virus type B* | 48649 |
|  | *Moraxella catarrhalis* | 707 |
|  | *Streptococcus anginosus* | 673 |
|  | *Acinetobacter baumannii* | 224 |
| 1037185 | *Human parainfluenza virus type 4* | 439 |
|  | *Herpes simplex virus type 1* | 238 |
|  | *Haemophilus influenzae* | 290 |
|  | *Streptococcus pneumoniae* | 110 |
|  | *Streptococcus intermedius* | 276 |
|  | *Acinetobacter baumannii* | 55 |
| 1038384 | *Mycoplasma pneumoniae* | 1230 |
|  | *Human parainfluenza virus type 3* | 183 |
|  | *Rhinovirus type C* | 100 |
| 1037508 | *Human coronavirus OC43* | 32693 |
|  | *Rhinovirus A* | 14268 |
|  | *Haemophilus influenzae* | 39324 |
|  | *Staphylococcus aureus* | 1739 |
|  | *Streptococcus intermedius* | 2625 |
| 1037175 | *Mycoplasma pneumoniae* | 14246 |
|  | *Haemophilus influenzae* | 177 |
| 1038527 | *Rhinovirus type B* | 3817 |
|  | *Moraxella catarrhalis* | 50 |
|  | *Streptococcus anginosus* | 86 |
| 1003043 | *Human parainfluenza virus type 3* | 28865 |
|  | *Rhinovirus A* | 13877 |
|  | *Haemophilus influenzae* | 30460 |
|  | *Streptococcus intermedius* | 4560 |
| 1037883 | *Human parainfluenza virus type 1* | 7658 |
|  | *Human bocavirus type 1* | 1569 |
|  | *Streptococcus pneumoniae* | 48432 |
|  | *Moraxella catarrhalis* | 55 |
| 1037657 | *Klebsiella pneumoniae* | 284 |
| 1037740 | *SARS-CoV-2 omicron XBB* | 1006 |
|  | *Mycoplasma pneumoniae* | 1007 |
|  | *Streptococcus anginosus* | 2987 |
| 1027661 | *Acinetobacter baumannii* | 52 |
| 0997979 | *Haemophilus influenzae* | 17 |
|  | *Streptococcus anginosus* | 156 |
|  | *Streptococcus intermedius* | 149 |
| 1038805 | *Human Metapneumovirus (HMPV)* | 54352 |
|  | *Streptococcus intermedius* | 838 |
| 1037424 | *Herpes simplex virus type 1* | 15684 |
|  | *Streptococcus constellatus* | 10765 |
| 1038028 | *Mycoplasma pneumoniae* | 61 |
|  | *Haemophilus influenzae* | 1917 |
|  | *Staphylococcus aureus* | 61 |
|  | *Streptococcus intermedius* | 231 |
| 1037082 | *Mycoplasma pneumoniae* | 4346 |
|  | *Streptococcus pneumoniae* | 43 |
|  | *Streptococcus constellatus* | 1466 |
| 1036995 | *Rhinovirus A* | 5393 |
|  | *Streptococcus intermedius* | 1063 |
| 1038130 | *Mycoplasma pneumoniae* | 18417 |
|  | *Streptococcus anginosus* | 66 |
| 1038598 | *Mycoplasma pneumoniae* | 5219 |
|  | *Haemophilus influenzae* | 153 |
|  | *Acinetobacter baumannii* | 1273 |
|  | *Streptococcus anginosus* | 2179 |
| 1037818 | *Rhinovirus A* | 630 |
|  | *Streptococcus pneumoniae* | 3586 |
|  | *Streptococcus anginosus* | 15416 |
| 1036917 | *Bordetella pertussis* | 4384 |
|  | *Streptococcus anginosus* | 276 |
| 1038274 | *Human parainfluenza virus type 1* | 8465 |
|  | *Moraxella catarrhalis* | 1317 |
|  | *Streptococcus intermedius* | 4799 |
| 1038387 | *Streptococcus pneumoniae* | 71 |
|  | *Streptococcus intermedius* | 206 |
| 1037498 | *Influenza A virus H3N2* | 29861 |
| 1037293 | *Human Metapneumovirus (HMPV)* | 37415 |
|  | *Moraxella catarrhalis* | 6964 |
|  | *Klebsiella pneumoniae* | 1066 |
|  | *Staphylococcus aureus* | 194 |
|  | *Acinetobacter baumannii* | 1908 |
|  | *Streptococcus intermedius* | 16678 |
| 1037830 | *Influenza A virus H3N2* | 2805 |
|  | *Streptococcus pneumoniae* | 94 |
|  | *Streptococcus constellatus* | 1448 |
| 959951 | *Mycoplasma pneumoniae* | 403 |
|  | *Human parainfluenza virus type 1* | 40 |
| 1038295 | *Haemophilus influenzae* | 72872 |
|  | *Moraxella catarrhalis* | 351 |
|  | *Streptococcus intermedius* | 12901 |
| 1037736 | *Coxsackievirus type A6* | 36850 |
|  | *Streptococcus anginosus* | 361 |
| 1036680 | *Mycoplasma pneumoniae* | 2013 |
| 1037092 | *Mycoplasma pneumoniae* | 60 |
|  | *Streptococcus intermedius* | 118 |
| 1037841 | *Human bocavirus type 1* | 49546 |
| 1036854 | *Streptococcus intermedius* | 61 |
| 1037937 | *Haemophilus influenzae* | 22 |
|  | *Streptococcus intermedius* | 41 |
| 995399 | *Human parainfluenza virus type 1* | 46728 |
|  | *Coxsackievirus type A6* | 27465 |
|  | *Rhinovirus A* | 112 |
|  | *Haemophilus influenzae* | 395 |
|  | *Moraxella catarrhalis* | 126 |
|  | *Streptococcus constellatus* | 274 |
| 1038547 | *Rhinovirus A* | 8777 |
|  | *Streptococcus anginosus* | 396 |
| 1038147 | *Coxsackievirus type A6* | 41097 |
|  | *Human parainfluenza virus type 3* | 90 |
|  | *Staphylococcus aureus* | 54 |
|  | *Acinetobacter baumannii* | 91 |
| 1038894 | *Human respiratory syncytial virus type B* | 60265 |
|  | *Human parainfluenza virus type 1* | 10136 |
|  | *Coxsackievirus type A6* | 101 |
|  | *Staphylococcus aureus* | 210 |
|  | *Pseudomonas aeruginosa* | 89 |
|  | *Streptococcus constellatus* | 1587 |
| 1038298 | *Streptococcus intermedius* | 2017 |
| 1038756 | *Streptococcus constellatus* | 470 |
| 1037578 | *Human respiratory syncytial virus type A* | 21244 |
|  | *Mycoplasma pneumoniae* | 1767 |
|  | *Haemophilus influenzae* | 262 |
|  | *Streptococcus constellatus* | 697 |
| 1038071 | *Influenza A virus H3N2* | 25098 |
|  | *Acinetobacter baumannii* | 128 |
| 1038301 | *Streptococcus anginosus* | 210 |
| 1036753 | *Human Metapneumovirus (HMPV)* | 57850 |
|  | *Streptococcus intermedius* | 68 |
| 1022505 | *Human parainfluenza virus type 3* | 20286 |
|  | *Haemophilus influenzae* | 23 |
|  | *Acinetobacter baumannii* | 58 |
| 1038344 | *Mycoplasma pneumoniae* | 434 |
|  | *Streptococcus anginosus* | 10122 |
| 1037848 | *Human bocavirus type 1* | 68502 |
|  | *Staphylococcus aureus* | 265 |
| 1037174 | *Bordetella pertussis* | 524 |
|  | *Haemophilus influenzae* | 4337 |
|  | *Streptococcus intermedius* | 153 |
| 981828 | *Mycoplasma pneumoniae* | 1393 |
|  | *Haemophilus influenzae* | 8606 |
|  | *Streptococcus intermedius* | 1251 |
| 1038824 | *Human bocavirus type 1* | 80754 |
|  | *Human parainfluenza virus type 3* | 42 |
|  | *Influenza A virus* | 30 |
|  | *Streptococcus pneumoniae* | 90 |
| 964386 | *Human parainfluenza virus type 3* | 31201 |
|  | *Coxsackievirus type A10* | 394 |
| 1038292 | *Rhinovirus A* | 17951 |
|  | *Bordetella pertussis* | 6317 |
|  | *Streptococcus pneumoniae* | 53760 |
|  | *Streptococcus anginosus* | 939 |
| 980125 | *Human parainfluenza virus type 1* | 43317 |
|  | *Rhinovirus A* | 8504 |
|  | *Streptococcus intermedius* | 2145 |
| 1022594 | *Rhinovirus A* | 4864 |
|  | *Streptococcus intermedius* | 305 |
| 1036792 | *Human adenovirus type 3* | 83277 |
|  | *Haemophilus influenzae* | 201 |
| 1038753 | *Mycoplasma pneumoniae* | 24225 |
| 1036765 | *Mycoplasma pneumoniae* | 35412 |
|  | *Streptococcus anginosus* | 2186 |
| 1038695 | *Mycoplasma pneumoniae* | 13296 |
| 1040411 | *Mycoplasma pneumoniae* | 5526 |
|  | *Haemophilus influenzae* | 311 |
|  | *Streptococcus intermedius* | 1376 |
| 1039767 | *Mycoplasma pneumoniae* | 519 |
|  | *Haemophilus influenzae* | 186 |
|  | *Streptococcus intermedius* | 81 |
| 1039945 | *Mycoplasma pneumoniae* | 6912 |
|  | *Streptococcus pneumoniae* | 290 |
|  | *Streptococcus constellatus* | 7328 |
| 1041340 | *Rhinovirus type B* | 14663 |
|  | *Mycoplasma pneumoniae* | 11926 |
| 1040546 | *Mycoplasma pneumoniae* | 5861 |
|  | *Rhinovirus A* | 222 |
|  | *Herpes simplex virus type 1* | 137 |
|  | *Streptococcus intermedius* | 139 |
| 1039358 | *Mycoplasma pneumoniae* | 30848 |
|  | *Staphylococcus aureus* | 16 |
| 1039428 | *Human bocavirus type 1* | 249 |
|  | *Streptococcus intermedius* | 55 |
| 1041326 | *Influenza A virus H3N2* | 36029 |
|  | *Haemophilus influenzae* | 1230 |
|  | *Staphylococcus aureus* | 55 |
|  | *Streptococcus constellatus* | 3573 |
| 1040155 | *Mycoplasma pneumoniae* | 4750 |
| 1040407 | *Mycoplasma pneumoniae* | 647 |
|  | *Rhinovirus type B* | 123 |
|  | *Streptococcus constellatus* | 1916 |
| 1041533 | *Influenza A virus* | 62 |
|  | *Haemophilus influenzae* | 6239 |
|  | *Streptococcus intermedius* | 2942 |
| 1039940 | *Coxsackievirus type A6* | 48374 |
| 1039942 | *Mycoplasma pneumoniae* | 15631 |
| 1039120 | *Mycoplasma pneumoniae* | 372 |
|  | *Streptococcus constellatus* | 141 |
| 1041129 | *Mycoplasma pneumoniae* | 10970 |
|  | *Acinetobacter baumannii* | 320 |
| 1040773 | *Bordetella pertussis* | 40179 |
|  | *Influenza A virus H3N2* | 33120 |
|  | *Haemophilus influenzae* | 47 |
|  | *Streptococcus anginosus* | 1403 |
|  | *Acinetobacter baumannii* | 759 |
| 1039198 | *Mycoplasma pneumoniae* | 1282 |
|  | *SARS-CoV-2 omicron XBB* | 252 |
|  | *Streptococcus anginosus* | 456 |
| 947153 | *Streptococcus pyogenes* | 3540 |
|  | *Human parainfluenza virus type 1* | 2027 |
|  | *Staphylococcus aureus* | 20 |
|  | *Streptococcus constellatus* | 3246 |
| 990617 | *Influenza A virus H3N2* | 28776 |
|  | *Haemophilus influenzae* | 38 |
|  | *Streptococcus constellatus* | 978 |
| 1040582 | *Mycoplasma pneumoniae* | 1617 |
| 1041428 | *Rhinovirus type B* | 27091 |
|  | *Human coronavirus OC43* | 13438 |
|  | *Haemophilus influenzae* | 149 |
|  | *Moraxella catarrhalis* | 55 |
| 1041330 | *Mycoplasma pneumoniae* | 32934 |
| 1039333 | *Human Metapneumovirus (HMPV)* | 14741 |
|  | *Influenza A virus H3N2* | 7333 |
|  | *Streptococcus anginosus* | 303 |
| 1039049 | *Human respiratory syncytial virus type B* | 48528 |
|  | *Haemophilus influenzae* | 1959 |
|  | *Staphylococcus aureus* | 648 |
|  | *Streptococcus intermedius* | 1749 |
| 1038940 | *Mycoplasma pneumoniae* | 1100 |
| 1039505 | *Mycoplasma pneumoniae* | 43871 |
|  | *Haemophilus influenzae* | 11 |
| 985612 | *Human adenovirus type 3* | 79124 |
|  | *Streptococcus pneumoniae* | 452 |
| 1021870 | *Rhinovirus* | 202 |
|  | *Mycoplasma pneumoniae* | 196 |
| 1041003 | *Human bocavirus type 1* | 61 |
|  | *Moraxella catarrhalis* | 148 |
| 1041531 | *Influenza A virus H3N2* | 16252 |
| 1039924 | *Rhinovirus type C* | 40915 |
|  | *Streptococcus pneumoniae* | 108 |
| 1039295 | *Human parainfluenza virus type 3* | 4021 |
|  | *Haemophilus influenzae* | 26 |
|  | *Acinetobacter baumannii* | 80 |
| 1028978 | *Human bocavirus type 1* | 68732 |
| 975356 | *Mycoplasma pneumoniae* | 895 |
| 1040916 | *Rhinovirus type C* | 7027 |
|  | *Mycoplasma pneumoniae* | 5924 |
|  | *Haemophilus influenzae* | 1733 |
|  | *Streptococcus intermedius* | 427 |
| 1019050 | *Rhinovirus type C* | 6034 |
|  | *Streptococcus pneumoniae* | 5555 |
| 1040152 | *Mycoplasma pneumoniae* | 334 |
|  | *Streptococcus constellatus* | 828 |
| 1039274 | *Mycoplasma pneumoniae* | 5660 |
| 1041507 | *Rhinovirus type C* | 199 |
| 1040793 | *Mycoplasma pneumoniae* | 7666 |
|  | *Haemophilus influenzae* | 7532 |
| 1040133 | *Mycoplasma pneumoniae* | 27090 |
|  | *Streptococcus pneumoniae* | 2705 |
|  | *Haemophilus influenzae* | 276 |
|  | *Staphylococcus aureus* | 28 |
|  | *Streptococcus constellatus* | 6407 |
|  | *Acinetobacter baumannii* | 974 |
| 1040308 | *Human respiratory syncytial virus type B* | 64588 |
|  | *Moraxella catarrhalis* | 688 |
|  | *Streptococcus anginosus* | 3430 |
| 1040073 | *Mycoplasma pneumoniae* | 676 |
| 991823 | *Streptococcus constellatus* | 311 |
| 992214 | *Coxsackievirus type A6* | 34483 |
|  | *Human Metapneumovirus (HMPV)* | 55 |
| 1040040 | *Human adenovirus type 3* | 83105 |
|  | *Herpes simplex virus type 1* | 139 |
|  | *Streptococcus pneumoniae* | 23 |
| 1039359 | *Haemophilus influenzae* | 346 |
|  | *Streptococcus constellatus* | 1608 |
| 1040056 | *Mycoplasma pneumoniae* | 43429 |
|  | *Staphylococcus aureus* | 264 |
|  | *Haemophilus influenzae* | 12 |
|  | *Streptococcus anginosus* | 1693 |
| 1041327 | *Mycoplasma pneumoniae* | 87 |
|  | *Streptococcus intermedius* | 53 |
| 1039882 | *Mycoplasma pneumoniae* | 4672 |
|  | *Streptococcus intermedius* | 153 |
| 909410 | *Rhinovirus* | 36923 |
|  | *Bordetella pertussis* | 4964 |
|  | *SARS-CoV-2 omicron XBB* | 446 |
|  | *Haemophilus influenzae* | 748 |
|  | *Staphylococcus aureus* | 184 |
|  | *Streptococcus anginosus* | 1174 |
| 1041287 | *Human Metapneumovirus (HMPV)* | 31845 |
| 1040164 | *Mycoplasma pneumoniae* | 9327 |
|  | *Influenza C virus* | 131 |
|  | *Staphylococcus aureus* | 80 |
| 1039216 | *Rhinovirus type C* | 410 |
|  | *Human coronavirus OC43* | 31 |
|  | *Moraxella catarrhalis* | 850 |
|  | *Streptococcus anginosus* | 402 |
| 1039369 | *Bordetella pertussis* | 384 |
|  | *Streptococcus intermedius* | 2355 |
| 1039936 | *Rhinovirus type C* | 54 |
|  | *Haemophilus influenzae* | 117 |
|  | *Klebsiella pneumoniae* | 83 |
|  | *Streptococcus constellatus* | 3374 |
|  | *Acinetobacter baumannii* | 2521 |
| 1041679 | *Influenza A virus H3N2* | 28997 |
|  | *Mycoplasma pneumoniae* | 20654 |
|  | *Rhinovirus type B* | 129 |
|  | *Staphylococcus aureus* | 248 |
|  | *Streptococcus intermedius* | 2765 |
| 989701 | *Influenza A virus* | 45 |
|  | *Streptococcus pneumoniae* | 714 |
|  | *Haemophilus influenzae* | 37 |
|  | *Streptococcus intermedius* | 966 |
| 1015129 | *Rhinovirus type B* | 47234 |
|  | *Bordetella pertussis* | 48 |
| 1039011 | *Mycoplasma pneumoniae* | 15400 |
| 1040505 | *Mycoplasma pneumoniae* | 1342 |
| 1039045 | *Human respiratory syncytial virus type A* | 48597 |
|  | *Coxsackievirus type A6* | 738 |
|  | *Haemophilus influenzae* | 21817 |
| 921079 | *Mycoplasma pneumoniae* | 20689 |
|  | *Human adenovirus type 3* | 1537 |
|  | *Streptococcus pneumoniae* | 25172 |
|  | *Klebsiella pneumoniae* | 46 |
| 1041106 | *Rhinovirus type B* | 2070 |
|  | *Haemophilus influenzae* | 3783 |
|  | *Staphylococcus aureus* | 11 |
|  | *Streptococcus intermedius* | 152 |
| 1041332 | *Mycoplasma pneumoniae* | 10363 |
| 1039519 | *Human parainfluenza virus type 3* | 2134 |
|  | *Staphylococcus aureus* | 303 |
|  | *Streptococcus constellatus* | 3456 |
|  | *Acinetobacter baumannii* | 192 |
| 1040509 | *Influenza A virus H3N2* | 58774 |
|  | *Mycoplasma pneumoniae* | 790 |
| 1040294 | *Mycoplasma pneumoniae* | 1517 |
| 1040420 | *Streptococcus pneumoniae* | 27970 |
|  | *Staphylococcus aureus* | 60 |
| 1041320 | *Mycoplasma pneumoniae* | 9937 |
|  | *Haemophilus influenzae* | 30 |
|  | *Streptococcus intermedius* | 251 |
| 1040687 | *Human Metapneumovirus (HMPV)* | 4536 |
|  | *Haemophilus influenzae* | 27 |
|  | *Streptococcus constellatus* | 1083 |
| 1038540 | *Streptococcus pneumoniae* | 747 |
| 1040396 | *Rhinovirus A* | 31015 |
|  | *Haemophilus influenzae* | 3177 |
|  | *Streptococcus anginosus* | 418 |
| 1041303 | *Human Metapneumovirus (HMPV)* | 17865 |
|  | *Human adenovirus type 3* | 6008 |
|  | *Streptococcus pneumoniae* | 29106 |
|  | *Moraxella catarrhalis* | 6690 |
|  | *Staphylococcus aureus* | 197 |
| 989615 | *Influenza A virus H3N2* | 448 |
|  | *Streptococcus anginosus* | 9534 |
| 1023009 | *Mycoplasma pneumoniae* | 25274 |
|  | *Streptococcus pneumoniae* | 194 |
|  | *Staphylococcus aureus* | 64 |
|  | *Streptococcus constellatus* | 2159 |
| 1038950 | *Mycoplasma pneumoniae* | 46747 |
|  | *Human coronavirus 229E* | 792 |
| 1040234 | *Mycoplasma pneumoniae* | 174 |
| 1041438 | *Influenza A virus H3N2* | 3197 |
|  | *Streptococcus pneumoniae* | 44092 |
|  | *Staphylococcus aureus* | 40 |
|  | *Haemophilus influenzae* | 30 |
|  | *Streptococcus constellatus* | 1163 |
| 955886 | *Rhinovirus type C* | 5238 |
|  | *Staphylococcus aureus* | 59 |
| 1040808 | *Mycoplasma pneumoniae* | 32697 |
|  | *Staphylococcus aureus* | 16 |
|  | *Streptococcus intermedius* | 392 |
| 1039977 | *Mycoplasma pneumoniae* | 4042 |
|  | *Streptococcus anginosus* | 804 |
| 1041623 | *Mycoplasma pneumoniae* | 27974 |
|  | *Rhinovirus type B* | 5665 |
|  | *Haemophilus influenzae* | 449 |
| 1038947 | *Human parainfluenza virus type 1* | 34 |
|  | *Haemophilus influenzae* | 12223 |
|  | *Streptococcus constellatus* | 2827 |
| 1041408 | *Influenza A virus H3N2* | 5648 |
|  | *Staphylococcus aureus* | 530 |
|  | *Streptococcus intermedius* | 157 |
| 1039706 | *Human bocavirus type 1* | 40104 |
|  | *Haemophilus influenzae* | 4713 |
|  | *Streptococcus pneumoniae* | 891 |
| 1040762 | *Mycoplasma pneumoniae* | 10627 |
|  | *Rhinovirus type C* | 9942 |
|  | *Streptococcus pneumoniae* | 2261 |
|  | *Haemophilus influenzae* | 57 |
| 1007122 | *Human coronavirus HKU1* | 21758 |
|  | *Rhinovirus type B* | 6492 |
|  | *Human coronavirus 229E* | 130 |
| 1013862 | *Mycoplasma pneumoniae* | 230 |
|  | *Coxsackievirus type A10* | 11 |
| 1040988 | *Human adenovirus type 3* | 68336 |
|  | *Herpes simplex virus type 1* | 154 |
|  | *Haemophilus influenzae* | 11067 |
|  | *Moraxella catarrhalis* | 2132 |
|  | *Streptococcus pneumoniae* | 893 |
| 1041325 | *Influenza A virus H3N2* | 78167 |
|  | *Human Metapneumovirus (HMPV)* | 420 |
| 1040925 | *Mycoplasma pneumoniae* | 48844 |
| 1040625 | *Mycoplasma pneumoniae* | 3249 |
|  | *Klebsiella pneumoniae* | 81 |
| 1040366 | *Rhinovirus type C* | 34012 |
|  | *Bordetella pertussis* | 671 |
|  | *Haemophilus influenzae* | 347 |
|  | *Streptococcus constellatus* | 2466 |
| 1040153 | *Mycoplasma pneumoniae* | 6324 |
|  | *Human Metapneumovirus (HMPV)* | 446 |
|  | *Bordetella pertussis* | 27 |
|  | *Streptococcus constellatus* | 582 |
|  | *Acinetobacter baumannii* | 165 |
| 898825 | *Human adenovirus type 3* | 9905 |
|  | *Streptococcus intermedius* | 49 |
| 1041505 | *Influenza A virus H3N2* | 77937 |
|  | *Haemophilus influenzae* | 858 |
| 1039225 | *Streptococcus anginosus* | 416 |
| 1039682 | *Bordetella pertussis* | 351 |
|  | *Human parainfluenza virus type 4* | 248 |
|  | *Rhinovirus A* | 211 |
|  | *Streptococcus anginosus* | 129 |
| 1040212 | *Mycoplasma pneumoniae* | 3429 |
| 1041615 | *Mycoplasma pneumoniae* | 52667 |
|  | *Rhinovirus A* | 70 |
|  | *Haemophilus influenzae* | 27 |
| 1039982 | *Mycoplasma pneumoniae* | 11529 |
|  | *Rhinovirus type B* | 97 |
|  | *Moraxella catarrhalis* | 197 |
|  | *Streptococcus intermedius* | 750 |
| 916484 | *Bordetella pertussis* | 22517 |
|  | *Mycoplasma pneumoniae* | 10952 |
|  | *Human respiratory syncytial virus type B* | 1491 |
| 1041059 | *Influenza A virus H3N2* | 44517 |
|  | *Streptococcus intermedius* | 101 |
| 908645 | *Human parainfluenza virus type 4* | 1878 |
|  | *Herpes simplex virus type 1* | 129 |
|  | *Haemophilus influenzae* | 67993 |
|  | *Streptococcus constellatus* | 629 |
| 1041652 | *Mycoplasma pneumoniae* | 3424 |
| 1040296 | *Mycoplasma pneumoniae* | 15972 |
|  | *Human coronavirus OC43* | 1102 |
|  | *Streptococcus anginosus* | 43 |
| 1039034 | *Mycoplasma pneumoniae* | 289 |
|  | *Acinetobacter baumannii* | 56 |
| 1039024 | *Rhinovirus type C* | 249 |
|  | *Streptococcus pneumoniae* | 32164 |
|  | *Streptococcus anginosus* | 2570 |
| 1040765 | *Mycoplasma pneumoniae* | 44175 |
|  | *Rhinovirus type B* | 4673 |
| 1041009 | *Mycoplasma pneumoniae* | 747 |
|  | *Influenza A virus* | 117 |
|  | *Haemophilus influenzae* | 444 |
|  | *Streptococcus anginosus* | 101 |
| 1040848 | *Mycoplasma pneumoniae* | 37879 |
|  | *Rhinovirus type B* | 14065 |
|  | *Haemophilus influenzae* | 59 |
|  | *Streptococcus constellatus* | 584 |
| 1041221 | *Influenza A virus H3N2* | 2495 |
|  | *Streptococcus anginosus* | 4372 |
| 1040832 | *Mycoplasma pneumoniae* | 10911 |
|  | *Streptococcus intermedius* | 1783 |
| 1040688 | *Human adenovirus type 3* | 76526 |
|  | *Streptococcus anginosus* | 109 |
| 866088 | *Mycoplasma pneumoniae* | 28583 |
|  | *Human parainfluenza virus type 3* | 55 |
| 1041624 | *Mycoplasma pneumoniae* | 46792 |
|  | *Herpes simplex virus type 1* | 21 |
|  | *Streptococcus pneumoniae* | 4593 |
|  | *Staphylococcus aureus* | 1561 |
| 1041135 | *Mycoplasma pneumoniae* | 397 |
|  | *Streptococcus anginosus* | 1588 |
| 1040520 | *Mycoplasma pneumoniae* | 2989 |
|  | *Staphylococcus aureus* | 130 |
| 1040444 | *Mycoplasma pneumoniae* | 28048 |
|  | *Haemophilus influenzae* | 262 |
| 1039591 | *Mycoplasma pneumoniae* | 315 |
|  | *Streptococcus intermedius* | 2158 |
| 1041695 | *Mycoplasma pneumoniae* | 59007 |
|  | *Staphylococcus aureus* | 26 |
|  | *Streptococcus intermedius* | 108 |
| 1041100 | *Human adenovirus type 3* | 76970 |
|  | *Mycoplasma pneumoniae* | 1516 |
|  | *Staphylococcus aureus* | 51 |
| 917695 | *Mycoplasma pneumoniae* | 26119 |
|  | *Human parainfluenza virus type 1* | 22307 |
| 1040724 | *Mycoplasma pneumoniae* | 4604 |
| 1041000 | *Influenza A virus H3N2* | 1841 |
|  | *Streptococcus intermedius* | 4628 |
|  | *Acinetobacter baumannii* | 435 |
| 1040314 | *Mycoplasma pneumoniae* | 2338 |
| 1038296 | *Bordetella pertussis* | 52 |
|  | *Haemophilus influenzae* | 22632 |
|  | *Streptococcus intermedius* | 999 |
| 1039960 | *Human adenovirus type 3* | 53879 |
|  | *Mycoplasma pneumoniae* | 2935 |
|  | *Haemophilus influenzae* | 3466 |
|  | *Streptococcus anginosus* | 3497 |
| 1041424 | *Mycoplasma pneumoniae* | 51822 |
|  | *Streptococcus pneumoniae* | 953 |
|  | *Haemophilus influenzae* | 133 |
| 1009681 | *Influenza A virus H3N2* | 50114 |
|  | *Moraxella catarrhalis* | 186 |
|  | *Streptococcus intermedius* | 181 |
| 1040327 | *Influenza A virus H3N2* | 15350 |
|  | *Streptococcus anginosus* | 1991 |
| 1039605 | *Haemophilus influenzae* | 2225 |
|  | *Streptococcus intermedius* | 3660 |
| 1041525 | *Influenza A virus* | 3939 |
|  | *Rhinovirus type C* | 455 |
|  | *Streptococcus pneumoniae* | 6871 |
| 1044516 | *Mycoplasma pneumoniae* | 4224 |
|  | *Rhinovirus type B* | 97 |
| 1042686 | *Mycoplasma pneumoniae* | 60757 |
| 1042802 | *Mycoplasma pneumoniae* | 6514 |
| 1042796 | *Human adenovirus type 3* | 62898 |
|  | *Bordetella pertussis* | 545 |
|  | *Mycoplasma pneumoniae* | 14 |
| 1043687 | *Human adenovirus type 3* | 74151 |
|  | *Streptococcus pyogenes* | 635 |
|  | *Moraxella catarrhalis* | 182 |
|  | *Influenza A virus* | 159 |
| 1044728 | *Moraxella catarrhalis* | 34213 |
|  | *Haemophilus influenzae* | 5758 |
|  | *Influenza A virus* | 1930 |
| 1044532 | *Mycoplasma pneumoniae* | 5330 |
|  | *Streptococcus intermedius* | 94 |
|  | *Streptococcus pneumoniae* | 69 |
|  | *Haemophilus influenzae* | 67 |
| 1041944 | *Mycoplasma pneumoniae* | 50395 |
|  | *Haemophilus influenzae* | 39 |
| 1044121 | *Mycoplasma pneumoniae* | 44581 |
|  | *Streptococcus intermedius* | 165 |
| 1043778 | *Mycoplasma pneumoniae* | 53442 |
|  | *Haemophilus influenzae* | 99 |
|  | *Staphylococcus aureus* | 34 |
| 1043516 | *Mycoplasma pneumoniae* | 7878 |
| 1044630 | *Mycoplasma pneumoniae* | 26221 |
|  | *Staphylococcus aureus* | 30 |
| 1044346 | *Influenza A virus H3N2* | 60999 |
|  | *Moraxella catarrhalis* | 18355 |
|  | *Streptococcus pneumoniae* | 16 |
| 1044465 | *Human respiratory syncytial virus type B* | 40565 |
|  | *Haemophilus influenzae* | 137 |
| 1043009 | *Mycoplasma pneumoniae* | 10117 |
|  | *Staphylococcus aureus* | 152 |
| 961500 | *Influenza A virus* | 3733 |
|  | *Streptococcus pneumoniae* | 968 |
| 1043010 | *Mycoplasma pneumoniae* | 43831 |
| 1024185 | *Human adenovirus type 3* | 76780 |
|  | *Haemophilus influenzae* | 1756 |
|  | *Human coronavirus 229E* | 379 |
| 993513 | *Mycoplasma pneumoniae* | 44105 |
|  | *Acinetobacter baumannii* | 61 |
|  | *Herpes simplex virus type 1* | 18 |
| 1042143 | *Human adenovirus type 3* | 48657 |
|  | *Mycoplasma pneumoniae* | 18552 |
|  | *Rhinovirus type C* | 5684 |
|  | *Haemophilus influenzae* | 989 |
|  | *Streptococcus pneumoniae* | 419 |
|  | *Moraxella catarrhalis* | 136 |
| 1043541 | *Influenza A virus H3N2* | 4110 |
|  | *Bordetella pertussis* | 2396 |
|  | *Haemophilus influenzae* | 817 |
| 1044865 | *Mycoplasma pneumoniae* | 16807 |
|  | *Rhinovirus A* | 1309 |
| 1044616 | *Human adenovirus type 3* | 32585 |
|  | *Streptococcus pneumoniae* | 1405 |
|  | *Mycoplasma pneumoniae* | 120 |
| 1043536 | *Haemophilus influenzae* | 1700 |
| 1043582 | *Mycoplasma pneumoniae* | 9163 |
|  | *Streptococcus pneumoniae* | 3737 |
|  | *Bordetella pertussis* | 1221 |
|  | *Influenza A virus H3N2* | 106 |
| 1042934 | *Mycoplasma pneumoniae* | 7773 |
| 987826 | *Mycoplasma pneumoniae* | 16045 |
|  | *Haemophilus influenzae* | 191 |
|  | *Streptococcus intermedius* | 41 |
|  | *Staphylococcus aureus* | 34 |
| 1044742 | *Mycoplasma pneumoniae* | 23725 |
|  | *Haemophilus influenzae* | 10925 |
|  | *Staphylococcus aureus* | 33 |
|  | *Influenza C virus* | 11 |
| 1042589 | *Rhinovirus A* | 49010 |
|  | *Haemophilus influenzae* | 982 |
|  | *Human parainfluenza virus type 1* | 285 |
| 1042279 | *Mycoplasma pneumoniae* | 43615 |
|  | *Streptococcus pneumoniae* | 1180 |
| 1043653 | *Haemophilus influenzae* | 13 |
| 1043774 | *Influenza A virus H3N2* | 5739 |
|  | *Staphylococcus aureus* | 222 |
| 1041921 | *Staphylococcus aureus* | 352 |
|  | *Moraxella catarrhalis* | 129 |
| 1044307 | *Human Metapneumovirus (HMPV)* | 51787 |
|  | *Haemophilus influenzae* | 32668 |
| 1042609 | *Influenza A virus* | 163 |
|  | *Streptococcus pneumoniae* | 160 |
| 1021820 | *Influenza A virus H3N2* | 38164 |
|  | *Staphylococcus aureus* | 75 |
| 976447 | *Human adenovirus type 3* | 68520 |
|  | *Human parainfluenza virus type 1* | 21 |
| 1044621 | *Acinetobacter baumannii* | 1636 |
|  | *Mycoplasma pneumoniae* | 1178 |
| 1042825 | *Mycoplasma pneumoniae* | 8169 |
|  | *Rhinovirus A* | 130 |
|  | *Bordetella pertussis* | 36 |
| 1042915 | *Streptococcus pneumoniae* | 22811 |
|  | *Influenza A virus H3N2* | 20745 |
|  | *Haemophilus influenzae* | 9737 |
|  | *Rhinovirus type C* | 108 |
|  | *Staphylococcus aureus* | 20 |
| 1043041 | *Streptococcus intermedius* | 104 |
|  | *Streptococcus dysgalactiae* | 8 |
| 1043112 | *Influenza A virus H3N2* | 45210 |
|  | *Streptococcus pneumoniae* | 1706 |
| 1042697 | *Human adenovirus type 3* | 80769 |
|  | *Haemophilus influenzae* | 422 |
|  | *Influenza A virus* | 370 |
| 1042180 | *Streptococcus pneumoniae* | 24906 |
|  | *Moraxella catarrhalis* | 245 |
|  | *Klebsiella pneumoniae* | 244 |
|  | *Acinetobacter baumannii* | 54 |
| 1043524 | *Influenza A virus* | 516 |
|  | *Haemophilus influenzae* | 105 |
| 1043802 | *Mycoplasma pneumoniae* | 9369 |
| 1030761 | *Influenza A virus* | 108 |
| 1044528 | *Rhinovirus A* | 7741 |
|  | *Bordetella pertussis* | 405 |
| 1042156 | *Streptococcus intermedius* | 92 |
|  | *Haemophilus influenzae* | 67 |
| 1044651 | *Influenza A virus H3N2* | 74599 |
|  | *Klebsiella pneumoniae* | 639 |
|  | *Acinetobacter baumannii* | 127 |
| 987246 | *Moraxella catarrhalis* | 51757 |
|  | *Pseudomonas aeruginosa* | 800 |
|  | *Influenza C virus* | 281 |
|  | *Streptococcus intermedius* | 71 |
|  | *Coxsackievirus type A6* | 34 |
|  | *Staphylococcus aureus* | 10 |
| 1043878 | *Mycoplasma pneumoniae* | 47592 |
|  | *Influenza A virus H3N2* | 431 |
|  | *Herpes simplex virus type 1* | 428 |
|  | *Human respiratory syncytial virus type B* | 36 |
| 1043583 | *Haemophilus influenzae* | 3244 |
|  | *Influenza A virus* | 21 |
| 1043727 | *Mycoplasma pneumoniae* | 21448 |
|  | *Moraxella catarrhalis* | 30 |
|  | *Rhinovirus type C* | 16 |
| 1042254 | *Human adenovirus type 3* | 80528 |
|  | *Haemophilus influenzae* | 51 |
| 1043157 | *Human respiratory syncytial virus type B* | 77445 |
| 1043140 | *Mycoplasma pneumoniae* | 10133 |
| 1042142 | *Streptococcus pneumoniae* | 27793 |
|  | *Acinetobacter baumannii* | 171 |
|  | *Staphylococcus aureus* | 75 |
|  | *Pseudomonas aeruginosa* | 62 |
| 1043313 | *Haemophilus influenzae* | 4950 |
|  | *Influenza A virus H3N2* | 583 |
|  | *Streptococcus intermedius* | 57 |
| 1042805 | *Mycoplasma pneumoniae* | 42255 |
|  | *Staphylococcus aureus* | 39 |
| 1042389 | *Human adenovirus type 3* | 60678 |
|  | *Mycoplasma pneumoniae* | 1675 |
|  | *Streptococcus pneumoniae* | 125 |
|  | *Herpes simplex virus type 1* | 18 |
| 1043877 | *Mycoplasma pneumoniae* | 660 |
|  | *Rhinovirus* | 406 |
|  | *Human Metapneumovirus (HMPV)* | 66 |
|  | *Streptococcus intermedius* | 49 |
| 1042888 | *Human Metapneumovirus (HMPV)* | 35382 |
|  | *Moraxella catarrhalis* | 7381 |
|  | *Haemophilus influenzae* | 7369 |
|  | *Mycoplasma pneumoniae* | 3140 |
|  | *Acinetobacter baumannii* | 82 |
| 1027851 | *Human respiratory syncytial virus type B* | 22864 |
|  | *Streptococcus pneumoniae* | 683 |
|  | *Acinetobacter baumannii* | 398 |
| 1043243 | *Mycoplasma pneumoniae* | 23632 |
|  | *Influenza A virus H3N2* | 359 |
| 1043514 | *Mycoplasma pneumoniae* | 1981 |
|  | *Acinetobacter baumannii* | 97 |
| 1042263 | *Haemophilus influenzae* | 1563 |
|  | *Streptococcus intermedius* | 179 |
| 1044035 | *Mycoplasma pneumoniae* | 2762 |
|  | *Rhinovirus type B* | 181 |
| 813339 | *Influenza A virus* | 233 |
|  | *Acinetobacter baumannii* | 98 |
|  | *Streptococcus intermedius* | 84 |
| 1044452 | *Streptococcus pneumoniae* | 67692 |
|  | *Human Metapneumovirus (HMPV)* | 15782 |
|  | *Moraxella catarrhalis* | 3842 |
|  | *Acinetobacter baumannii* | 363 |
| 1038346 | *Streptococcus intermedius* | 295 |
|  | *Staphylococcus aureus* | 19 |
| 1043909 | *Herpes simplex virus type 1* | 6736 |
|  | *Influenza A virus H3N2* | 1843 |
| 1043192 | *Mycoplasma pneumoniae* | 56270 |
| 1043035 | *Influenza A virus H3N2* | 37148 |
|  | *Mycoplasma pneumoniae* | 26015 |
|  | *Rhinovirus type C* | 600 |
| 1043217 | *Mycoplasma pneumoniae* | 23824 |
| 1043006 | *Human adenovirus type 3* | 79431 |
|  | *Mycoplasma pneumoniae* | 15 |
| 1044824 | *Mycoplasma pneumoniae* | 3003 |
| 1038513 | *Human adenovirus type 3* | 81199 |
|  | *Streptococcus intermedius* | 276 |
|  | *Haemophilus influenzae* | 25 |
| 1044525 | *Human respiratory syncytial virus type A* | 21323 |
|  | *Influenza A virus* | 156 |
| 1043111 | *Rhinovirus A* | 5779 |
|  | *Bordetella pertussis* | 2750 |
|  | *Influenza A virus* | 601 |
|  | *Acinetobacter baumannii* | 70 |
| 1042713 | *Mycoplasma pneumoniae* | 49059 |
| 1042799 | *Mycoplasma pneumoniae* | 39439 |
|  | *Streptococcus pneumoniae* | 120 |
|  | *Streptococcus intermedius* | 84 |
| 872669 | *Human adenovirus type 3* | 63663 |
|  | *Staphylococcus aureus* | 13 |
| 1042352 | *Mycoplasma pneumoniae* | 11488 |
| 1044294 | *Influenza A virus H3N2* | 2564 |
| 1043047 | *Mycoplasma pneumoniae* | 25797 |
| 1044120 | *Mycoplasma pneumoniae* | 43391 |
|  | *Haemophilus influenzae* | 658 |
| 879257 | *Mycoplasma pneumoniae* | 10223 |
| 1044338 | *Streptococcus pyogenes* | 55755 |
|  | *Human Metapneumovirus (HMPV)* | 716 |
| 1043105 | *Mycoplasma pneumoniae* | 2815 |
|  | *Streptococcus intermedius* | 195 |
|  | *Staphylococcus aureus* | 60 |
| 1043517 | *Human adenovirus type 5* | 22885 |
|  | *Moraxella catarrhalis* | 158 |
| 1042562 | *Human respiratory syncytial virus type B* | 6671 |
|  | *Acinetobacter baumannii* | 90 |
| 1043989 | *Mycoplasma pneumoniae* | 288 |
|  | *Moraxella catarrhalis* | 87 |
| 1044445 | *Mycoplasma pneumoniae* | 18495 |
|  | *Haemophilus influenzae* | 89 |
| 1044813 | *Haemophilus influenzae* | 1006 |
|  | *Streptococcus pneumoniae* | 352 |
| 1044519 | *Influenza A virus H3N2* | 33821 |
|  | *Mycoplasma pneumoniae* | 4175 |
| 1044534 | *Influenza A virus H3N2* | 11593 |
|  | *Mycoplasma pneumoniae* | 425 |
| 1043506 | *Human adenovirus type 3* | 70322 |
| 1044245 | *Mycoplasma pneumoniae* | 17178 |
| 1044457 | *Human respiratory syncytial virus type B* | 63469 |
|  | *Streptococcus pneumoniae* | 3364 |
|  | *Staphylococcus aureus* | 289 |
| 1042027 | *Mycoplasma pneumoniae* | 12923 |
| 1041916 | *Influenza A virus H3N2* | 8833 |
|  | *Bordetella pertussis* | 8041 |
|  | *Streptococcus pneumoniae* | 251 |
|  | *Klebsiella pneumoniae* | 205 |
|  | *Streptococcus intermedius* | 181 |
| 1043518 | *Rhinovirus type B* | 10937 |
|  | *Influenza A virus H3N2* | 5356 |
|  | *Haemophilus influenzae* | 576 |
| 1042187 | *Haemophilus influenzae* | 13248 |
|  | *Streptococcus intermedius* | 183 |
|  | *Rhinovirus type C* | 71 |
| 1042685 | *Haemophilus influenzae* | 1372 |
| 1042259 | *Influenza A virus H3N2* | 15321 |
|  | *Streptococcus intermedius* | 2573 |
| 824505 | *Streptococcus pneumoniae* | 47722 |
|  | *Influenza A virus H3N2* | 7149 |
|  | *Haemophilus influenzae* | 120 |
| 1042835 | *Streptococcus pneumoniae* | 42995 |
|  | *Influenza A virus* | 82 |
| 1042818 | *Mycoplasma pneumoniae* | 57723 |
| 1042449 | *Haemophilus influenzae* | 1026 |
|  | *Streptococcus pneumoniae* | 34 |
|  | *Staphylococcus aureus* | 24 |
| 1042176 | *Influenza A virus H3N2* | 11201 |
|  | *Streptococcus intermedius* | 127 |
| 835529 | *Mycoplasma pneumoniae* | 3749 |
|  | *Streptococcus constellatus* | 1172 |
|  | *Acinetobacter baumannii* | 297 |
|  | *Streptococcus pneumoniae* | 38 |
| 1043193 | *Influenza A virus H3N2* | 51153 |
|  | *Mycoplasma pneumoniae* | 6217 |
| 1043113 | *Mycoplasma pneumoniae* | 54228 |
|  | *Influenza C virus* | 162 |
|  | *Rhinovirus type C* | 113 |
|  | *Staphylococcus aureus* | 57 |
| 1042157 | *Human adenovirus type 3* | 59713 |
|  | *Streptococcus intermedius* | 282 |
| 1044828 | *Rhinovirus type C* | 4543 |
|  | *Mycoplasma pneumoniae* | 2527 |
| 1043530 | *Mycoplasma pneumoniae* | 507 |
|  | *Acinetobacter baumannii* | 189 |
| 1043528 | *Mycoplasma pneumoniae* | 1841 |
| 1024755 | *Mycoplasma pneumoniae* | 35374 |
| 1042154 | *Mycoplasma pneumoniae* | 8207 |
|  | *Bordetella pertussis* | 1396 |
|  | *Haemophilus influenzae* | 712 |
|  | *Rhinovirus A* | 253 |
| 1043212 | *Human adenovirus type 3* | 54318 |
|  | *Influenza A virus H3N2* | 27955 |
|  | *Mycoplasma pneumoniae* | 878 |
| 1043123 | *Haemophilus influenzae* | 3642 |
| 1041912 | *Streptococcus pyogenes* | 77554 |
|  | *Streptococcus intermedius* | 288 |
|  | *Rhinovirus type C* | 113 |
|  | *Coxsackievirus type A6* | 30 |
| 1044257 | *Human adenovirus type 3* | 81612 |
| 1043396 | *Mycoplasma pneumoniae* | 16467 |
| 1044123 | *Mycoplasma pneumoniae* | 28639 |
|  | *Streptococcus pneumoniae* | 12637 |
|  | *Rhinovirus type C* | 10034 |
|  | *Haemophilus influenzae* | 193 |
| 1044737 | *Mycoplasma pneumoniae* | 865 |
|  | *Human adenovirus type 3* | 363 |
| 1043711 | *Mycoplasma pneumoniae* | 3540 |
|  | *Rhinovirus type C* | 843 |
| 1043433 | *Rhinovirus type B* | 10593 |
|  | *Mycoplasma pneumoniae* | 9627 |
| 1042806 | *Haemophilus influenzae* | 81424 |
|  | *Human bocavirus type 1* | 235 |
|  | *Staphylococcus aureus* | 62 |
| 1042811 | *Human adenovirus type 3* | 39617 |
|  | *Mycoplasma pneumoniae* | 23983 |
|  | *Moraxella catarrhalis* | 1758 |
| 1023441 | *Influenza A virus H3N2* | 39501 |
|  | *Streptococcus pneumoniae* | 19954 |
|  | *Mycoplasma pneumoniae* | 69 |
| 1043588 | *Rhinovirus type B* | 2246 |
|  | *Staphylococcus aureus* | 139 |
|  | *Streptococcus intermedius* | 26 |
| 1042597 | *Mycoplasma pneumoniae* | 7858 |
| 1043804 | *Human Metapneumovirus (HMPV)* | 19560 |
|  | *Influenza A virus H3N2* | 5571 |
|  | *Enterovirus group B* | 178 |
|  | *Streptococcus intermedius* | 97 |
| 1044432 | *Human adenovirus type 3* | 29046 |
|  | *Mycoplasma pneumoniae* | 69 |
|  | *Influenza A virus* | 32 |
| 990144 | *Influenza A virus H3N2* | 32495 |
| 1043132 | *Influenza A virus H3N2* | 45993 |
|  | *Moraxella catarrhalis* | 78 |
|  | *Acinetobacter baumannii* | 45 |
| 1044248 | *Human respiratory syncytial virus type B* | 6544 |
| 1042358 | *Mycoplasma pneumoniae* | 39095 |
| 1042893 | *Mycoplasma pneumoniae* | 36803 |
| 1043188 | *Influenza A virus H3N2* | 41600 |
|  | *Rhinovirus type B* | 11379 |
|  | *Mycoplasma pneumoniae* | 8800 |
| 1043312 | *Mycoplasma pneumoniae* | 19890 |
|  | *Staphylococcus aureus* | 567 |
|  | *Streptococcus pneumoniae* | 20 |
| 992215 | *Human adenovirus type 3* | 80300 |
| 1044721 | *Human adenovirus type 3* | 30520 |
|  | *Influenza A virus* | 81 |
|  | *Streptococcus pneumoniae* | 71 |
| 1043211 | *Haemophilus influenzae* | 328 |
| 1042834 | *Streptococcus pneumoniae* | 23296 |
|  | *Influenza A virus H3N2* | 1334 |
|  | *Mycoplasma pneumoniae* | 1165 |
| 1041313 | *Influenza A virus H3N2* | 2368 |
| 1042172 | *Bordetella pertussis* | 85297 |
|  | *Influenza A virus H3N2* | 6695 |
|  | *Mycoplasma pneumoniae* | 125 |
|  | *Rhinovirus* | 118 |
|  | *Streptococcus intermedius* | 32 |
| 1043116 | *Mycoplasma pneumoniae* | 42013 |
|  | *Bordetella pertussis* | 2607 |
| 1043656 | *Haemophilus influenzae* | 170 |
|  | *Influenza A virus* | 31 |
| 1043496 | *Influenza A virus H3N2* | 72921 |
|  | *Human bocavirus type 1* | 193 |
|  | *Streptococcus intermedius* | 85 |
| 1041812 | *Influenza A virus H3N2* | 47613 |
|  | *Rhinovirus A* | 1531 |
|  | *Human adenovirus type 1* | 725 |
|  | *Haemophilus influenzae* | 20010 |
|  | *Streptococcus intermedius* | 161 |
| 1043289 | *Mycoplasma pneumoniae* | 2791 |
|  | *Streptococcus pneumoniae* | 279 |
|  | *Haemophilus influenzae* | 162 |
| 1043691 | *Human adenovirus type 3* | 69945 |
|  | *Mycoplasma pneumoniae* | 826 |
|  | *Acinetobacter baumannii* | 125 |
|  | *Moraxella catarrhalis* | 48 |
| 1043511 | *Human adenovirus type 3* | 54815 |
|  | *Mycoplasma pneumoniae* | 9272 |
|  | *Moraxella catarrhalis* | 81 |
|  | *Streptococcus pneumoniae* | 60 |
|  | *Staphylococcus aureus* | 15 |
| 1043894 | *Mycoplasma pneumoniae* | 10477 |
|  | *Acinetobacter baumannii* | 54 |
| 831862 | *Mycoplasma pneumoniae* | 19106 |
| 1043885 | *Mycoplasma pneumoniae* | 57921 |
|  | *Moraxella catarrhalis* | 209 |
| 1041820 | *Human bocavirus type 1* | 61503 |
|  | *Staphylococcus aureus* | 1088 |
|  | *Moraxella catarrhalis* | 615 |
|  | *Streptococcus anginosus* | 113 |
| 1044236 | *Mycoplasma pneumoniae* | 4454 |
|  | *Human adenovirus type 3* | 3526 |
| 1043992 | *Streptococcus pneumoniae* | 44325 |
|  | *Influenza A virus H3N2* | 25102 |
|  | *Moraxella catarrhalis* | 143 |
| 914412 | *Mycoplasma pneumoniae* | 17309 |
| 1042836 | *Mycoplasma pneumoniae* | 47529 |
| 1042812 | *Influenza A virus H3N2* | 21312 |
|  | *Mycoplasma pneumoniae* | 7089 |
| 1044039 | *Human respiratory syncytial virus type A* | 12229 |
|  | *Streptococcus pneumoniae* | 2535 |
|  | *Staphylococcus aureus* | 21 |
| 1041964 | *Influenza A virus H3N2* | 49312 |
|  | *Moraxella catarrhalis* | 238 |
|  | *Haemophilus influenzae* | 105 |
| 1044817 | *Streptococcus pneumoniae* | 922 |
| 1044468 | *Mycoplasma pneumoniae* | 29647 |
|  | *Streptococcus pneumoniae* | 6868 |
|  | *Haemophilus influenzae* | 122 |
|  | *Staphylococcus aureus* | 16 |
| 1042990 | *Staphylococcus aureus* | 266 |
|  | *Haemophilus influenzae* | 80 |
|  | *Influenza A virus* | 41 |
| 1043415 | *Mycoplasma pneumoniae* | 52124 |
|  | *Streptococcus pneumoniae* | 359 |
| 1042428 | *Influenza A virus H3N2* | 36255 |
|  | *Streptococcus pneumoniae* | 35568 |
|  | *Moraxella catarrhalis* | 2508 |
| 1042830 | *Human bocavirus type 1* | 16774 |
|  | *Human respiratory syncytial virus type B* | 1159 |
| 1043330 | *Streptococcus pneumoniae* | 171 |
| 1044286 | *Mycoplasma pneumoniae* | 34538 |
|  | *Moraxella catarrhalis* | 386 |
|  | *Human adenovirus type 3* | 74 |
| 1042474 | *Mycoplasma pneumoniae* | 52702 |
| 1042552 | *Human adenovirus type 3* | 70358 |
|  | *Acinetobacter baumannii* | 56 |
| 1043801 | *Haemophilus influenzae* | 10630 |
|  | *Influenza A virus H3N2* | 1412 |
| 1042599 | *Mycoplasma pneumoniae* | 12607 |
|  | *Rhinovirus type C* | 100 |
| 1043702 | *Mycoplasma pneumoniae* | 23200 |
|  | *Moraxella catarrhalis* | 197 |
| 1044435 | *Influenza A virus H3N2* | 35502 |
|  | *Streptococcus pneumoniae* | 11683 |
| 1044438 | *Human adenovirus type 3* | 51720 |
|  | *Mycoplasma pneumoniae* | 9986 |
| 1042111 | *Streptococcus intermedius* | 87 |
|  | *Streptococcus pneumoniae* | 41 |
| 1042446 | *Mycoplasma pneumoniae* | 43655 |
| 1043198 | *Mycoplasma pneumoniae* | 57371 |
| 1043914 | *Mycoplasma pneumoniae* | 4850 |
| 1042667 | *Human Metapneumovirus (HMPV)* | 391 |
| 1043544 | *Human adenovirus type 3* | 62924 |
|  | *Haemophilus influenzae* | 2746 |
|  | *Rhinovirus type C* | 201 |
| 1044136 | *Bordetella pertussis* | 35564 |
|  | *Haemophilus influenzae* | 944 |
|  | *Staphylococcus aureus* | 374 |
| 1042173 | *Haemophilus influenzae* | 1203 |
|  | *Streptococcus intermedius* | 175 |
| 1042930 | *Influenza A virus H3N2* | 5731 |
|  | *Rhinovirus type C* | 362 |
|  | *Bordetella pertussis* | 121 |
| 1044711 | *Human Metapneumovirus (HMPV)* | 460 |
|  | *Streptococcus intermedius* | 429 |
|  | *Haemophilus influenzae* | 188 |
| 1042957 | *Human adenovirus type 3* | 63929 |
|  | *Rhinovirus type C* | 12690 |
|  | *Haemophilus influenzae* | 3617 |
|  | *Streptococcus pneumoniae* | 773 |
| 1042184 | *Influenza A virus H3N2* | 16151 |
|  | *Haemophilus influenzae* | 1342 |
|  | *Streptococcus pneumoniae* | 435 |
| 1043394 | *Streptococcus pneumoniae* | 18397 |
|  | *Influenza A virus H3N2* | 11854 |
|  | *Bordetella pertussis* | 18 |
| 1043031 | *Influenza A virus H3N2* | 75557 |
|  | *Haemophilus influenzae* | 1092 |
| 1041954 | *Influenza A virus H3N2* | 5196 |
|  | *Haemophilus influenzae* | 241 |
| 1042919 | *Mycoplasma pneumoniae* | 27173 |
|  | *Streptococcus pneumoniae* | 154 |
| 978338 | *Mycoplasma pneumoniae* | 8297 |
| 1043999 | *Haemophilus influenzae* | 1115 |
|  | *Klebsiella pneumoniae* | 32 |
| 1043855 | *Mycoplasma pneumoniae* | 46980 |
|  | *Haemophilus influenzae* | 319 |
| 1044116 | *Human adenovirus type 3* | 72839 |
|  | *Staphylococcus aureus* | 123 |
| 1043402 | *Influenza A virus* | 468 |
|  | *Streptococcus intermedius* | 145 |
| 1042168 | *Human adenovirus type 3* | 89448 |
|  | *Haemophilus influenzae* | 279 |
| 1043772 | *Influenza A virus H3N2* | 47673 |
| 1043303 | *Influenza A virus H3N2* | 9843 |
|  | *Moraxella catarrhalis* | 739 |
|  | *Acinetobacter baumannii* | 226 |
|  | *Haemophilus influenzae* | 70 |
| 1044184 | *Influenza A virus H3N2* | 59403 |
|  | *Haemophilus influenzae* | 21 |
| 978186 | *Human Metapneumovirus (HMPV)* | 7586 |
|  | *Streptococcus intermedius* | 862 |
| 1041962 | *Human adenovirus type 3* | 79630 |
|  | *Rhinovirus type B* | 3834 |
|  | *Haemophilus influenzae* | 2835 |
|  | *Streptococcus intermedius* | 68 |
| 1044259 | *Mycoplasma pneumoniae* | 22266 |
| 1042088 | *Mycoplasma pneumoniae* | 4003 |
|  | *Streptococcus anginosus* | 39 |
| 1042177 | *Mycoplasma pneumoniae* | 50523 |
|  | *Rhinovirus A* | 635 |
|  | *Bordetella pertussis* | 68 |
| 1044150 | *Human Metapneumovirus (HMPV)* | 1988 |
|  | *Staphylococcus aureus* | 344 |
|  | *Streptococcus intermedius* | 32 |
| 980054 | *Streptococcus pneumoniae* | 252 |
| 1045593 | *Human adenovirus type 3* | 69656 |
|  | *Haemophilus influenzae* | 110 |
| 1045660 | *Human adenovirus type 3* | 33509 |
| 917824 | *Mycoplasma pneumoniae* | 85 |
| 1045033 | *Human respiratory syncytial virus type B* | 14879 |
|  | *Human coronavirus 229E* | 132 |
|  | *Streptococcus intermedius* | 84 |
|  | *Haemophilus influenzae* | 46 |
| 1045152 | *Mycoplasma pneumoniae* | 7177 |
|  | *Haemophilus influenzae* | 32 |
| 1045358 | *Mycoplasma pneumoniae* | 10900 |
| 1045585 | *Staphylococcus aureus* | 3375 |
|  | *Klebsiella pneumoniae* | 2826 |
|  | *Acinetobacter baumannii* | 1486 |
|  | *Human adenovirus type 5* | 114 |
|  | *Moraxella catarrhalis* | 40 |
| 1045817 | *Human respiratory syncytial virus type B* | 15265 |
|  | *Mycoplasma pneumoniae* | 12202 |
|  | *Bordetella pertussis* | 24 |
| 1045858 | *Human adenovirus type 3* | 46118 |
|  | *Mycoplasma pneumoniae* | 5545 |
|  | *Influenza A virus H3N2* | 3488 |
| 1045583 | *Human adenovirus type 3* | 54798 |
|  | *Moraxella catarrhalis* | 865 |
| 1045818 | *Rhinovirus type B* | 63 |
| 1045589 | *Mycoplasma pneumoniae* | 712 |
|  | *Streptococcus pneumoniae* | 130 |
| 1045458 | *Mycoplasma pneumoniae* | 46245 |
|  | *Human respiratory syncytial virus type B* | 4870 |
| 1045873 | *Human respiratory syncytial virus type A* | 27 |
|  | *Mycoplasma pneumoniae* | 17 |
| 1045575 | *Human adenovirus type 3* | 65623 |
|  | *Mycoplasma pneumoniae* | 401 |
| 1045268 | *Acinetobacter baumannii* | 475 |
|  | *Influenza A virus* | 234 |
| 1045906 | *Human respiratory syncytial virus type B* | 53094 |
|  | *Haemophilus influenzae* | 1764 |
|  | *Mycoplasma pneumoniae* | 138 |
|  | *Human Metapneumovirus (HMPV)* | 68 |
| 1045248 | *Influenza A virus H3N2* | 3341 |
|  | *Haemophilus influenzae* | 1643 |
|  | *Moraxella catarrhalis* | 359 |
| 1045181 | *Human adenovirus type 3* | 68431 |
|  | *Streptococcus pneumoniae* | 2569 |
|  | *Moraxella catarrhalis* | 113 |
| 1045280 | *Human adenovirus type 3* | 57550 |
|  | *Staphylococcus aureus* | 125 |
| 1045790 | *Human adenovirus type 3* | 14230 |
|  | *Mycoplasma pneumoniae* | 5398 |
|  | *Streptococcus pneumoniae* | 30 |
| 1045763 | *Mycoplasma pneumoniae* | 26 |
| 1045711 | *Human adenovirus type 3* | 42343 |
|  | *Streptococcus pneumoniae* | 8112 |
| 1045694 | *Mycoplasma pneumoniae* | 10313 |
| 1045601 | *Human Metapneumovirus (HMPV)* | 215 |
| 874435 | *Staphylococcus aureus* | 37 |
|  | *Herpes simplex virus type 1* | 26 |
| 981050 | *Mycoplasma pneumoniae* | 122 |
|  | *Acinetobacter baumannii* | 67 |
| 1045792 | *Haemophilus influenzae* | 106 |
| 1045918 | *Mycoplasma pneumoniae* | 4031 |
|  | *Rhinovirus A* | 412 |
|  | *Herpes simplex virus type 1* | 143 |
| 1045791 | *Mycoplasma pneumoniae* | 4495 |
| 1045278 | *Mycoplasma pneumoniae* | 4677 |
| 1045170 | *Human adenovirus type 3* | 81823 |
| 1045498 | *Human adenovirus type 3* | 27215 |
| 1045134 | *Mycoplasma pneumoniae* | 4632 |
|  | *Human respiratory syncytial virus type B* | 3189 |
|  | *Haemophilus influenzae* | 1011 |
|  | *Moraxella catarrhalis* | 34 |
| 1044944 | *Mycoplasma pneumoniae* | 2807 |
|  | *Streptococcus pneumoniae* | 458 |
|  | *Haemophilus influenzae* | 109 |
|  | *Influenza A virus* | 43 |
| 1044953 | *Rhinovirus* | 44077 |
| 1044975 | *Human Metapneumovirus (HMPV)* | 59 |
|  | *Streptococcus intermedius* | 36 |
| 1045765 | *Influenza A virus H3N2* | 55124 |
|  | *Haemophilus influenzae* | 187 |
| 1044959 | *Influenza A virus* | 2336 |
| 959630 | *Human Metapneumovirus (HMPV)* | 7484 |
| 1045209 | *Human adenovirus type 3* | 57379 |
|  | *Haemophilus influenzae* | 753 |
| 921567 | *Mycoplasma pneumoniae* | 30821 |
|  | *Herpes simplex virus type 1* | 320 |
| 1044841 | *Human adenovirus type 3* | 147 |
| 1044939 | *Human Metapneumovirus (HMPV)* | 185 |
| 1045131 | *Human respiratory syncytial virus type B* | 47253 |
|  | *Influenza A virus* | 7958 |
|  | *Human adenovirus type 3* | 1084 |
|  | *Human Metapneumovirus (HMPV)* | 399 |
| 1045150 | *Bordetella pertussis* | 59220 |
|  | *Human parainfluenza virus type 1* | 33 |
| 1044938 | *Mycoplasma pneumoniae* | 2186 |
|  | *Streptococcus pneumoniae* | 1334 |
| 1045144 | *Human respiratory syncytial virus type B* | 14114 |
| 1045998 | *Mycoplasma pneumoniae* | 17155 |
| 1023344 | *Human respiratory syncytial virus type B* | 7649 |
| 1045236 | *Human adenovirus type 3* | 9544 |
|  | *Mycoplasma pneumoniae* | 7931 |
| 925535 | *Human adenovirus type 3* | 32755 |
| 1045889 | *Human adenovirus type 3* | 2546 |
| 874174 | *Human Metapneumovirus (HMPV)* | 935 |
|  | *Streptococcus pneumoniae* | 14 |
| 1045357 | *Mycoplasma pneumoniae* | 1785 |
| 1044947 | *Mycoplasma pneumoniae* | 1398 |
| 1045811 | *Mycoplasma pneumoniae* | 19537 |
|  | *Haemophilus influenzae* | 118 |
|  | *Streptococcus pneumoniae* | 30 |
| 1045546 | *Mycoplasma pneumoniae* | 13463 |
|  | *Human respiratory syncytial virus type B* | 943 |
|  | *Streptococcus pneumoniae* | 26 |
| 1045928 | *Human adenovirus type 3* | 75134 |
|  | *Bordetella pertussis* | 1551 |
|  | *Influenza A virus* | 1072 |
|  | *Haemophilus influenzae* | 104 |
| 895464 | *Influenza A virus H3N2* | 41105 |
|  | *Haemophilus influenzae* | 1346 |
| 1034777 | *Human respiratory syncytial virus type A* | 13323 |
|  | *Klebsiella pneumoniae* | 49 |
