# Supplementary material for: Diagnostic and Clinical Value of Targeted Next‐Generation Sequencing for Pediatric Respiratory Infections in Northern China
Source: Clin Respir J. 2026 Apr 12;20(4):e70185. doi: 10.1111/crj.70185 (PMC13070711; doi:10.1111/crj.70185)
Supplement: Supplementary file 5 — Table S4: Criteria of treatment plan adjustments and response based on tNGS diagnosis. [file CRJ-20-e70185-s006.docx]

| **Supplementary Table 4 Criteria of Treatment Plan Adjustments and Response Based on tNGS Diagnosis.** | | |
| --- | --- | --- |
| **Criteria for Evaluating Treatment Plan Adjustments:** |  |  |
| Escalation | De-escalation | No Change |
| Addition of new antibiotics | Discontinuation of antibiotics | Keep original treatment plan |
| Increase in variety of antibiotics | Reduction in antibiotic potency | Parental refusal to change the treatment |
| Escalation to higher-grade antibiotics | Shortening of antibiotic treatment duration |  |
| Extension of antibiotic treatment duration | Decrease in combination antibiotic use |  |
| Transition to second-line drugs | Discontinuation of antiviral drugs |  |
| Addition of antiviral treatment | Shortening of antiviral treatment duration |  |
| Extension of antiviral treatment duration | Reduction in diagnostic tests and examinations |  |
| Increase in diagnostic tests and examinations | Reduction in symptomatic treatment drugs |  |
| Addition of symptomatic treatment drugs | Shortening of symptomatic treatment duration |  |
| Extension of symptomatic treatment duration | Decrease in frequency of symptomatic treatments |  |
| Increase in frequency of symptomatic treatments | Reduction of non-pharmacological treatments |  |
| Introduction of non-pharmacological treatment methods |  |  |
| Transfer to a higher-level hospital for further treatment |  |  |
| **Criteria for Evaluating Treatment Response:** |  |  |
| Improvement | Deterioration | No Change |
| Improvement in clinical symptoms and signs | Recurrent or increased body temperature | Patient discharged |
| Normalization or improvement in body temperature | Recurrent or worsened cough | Short hospital stay with no significant changes |
| Reduction or disappearance of cough | Recurrent or worsened chest tightness |  |
| Reduction or disappearance of chest tightness | Recurrent or worsened abdominal pain |  |
| Reduction or disappearance of abdominal pain | Recurrent or worsened other discomforts |  |
| Relief or disappearance of other discomforts | Worsened pharyngeal congestion |  |
| Reduction in pharyngeal congestion | Increased tonsillar congestion |  |
| Reduction or disappearance of tonsillar congestion | Increased or expanded purulent tonsillar secretions |  |
| Reduction or disappearance of purulent tonsillar secretions | Increased lung rales |  |
| Reduction or disappearance of lung rales |  |  |
| Reduction or disappearance of other positive signs |  |  |
