# Supplementary material for: Diagnostic and Clinical Value of Targeted Next‐Generation Sequencing for Pediatric Respiratory Infections in Northern China
Source: Clin Respir J. 2026 Apr 12;20(4):e70185. doi: 10.1111/crj.70185 (PMC13070711; doi:10.1111/crj.70185)
Supplement: Supplementary file 6 — Table S5: Clinical diagnosis, treatment, and outcomes for collected patients. [file CRJ-20-e70185-s003.docx]

| **Supplementary Table 5. Clinical diagnosis, treatment and outcomes for collected patients.** | | | | |  |  |  |  |
| --- | --- | --- | --- | --- | --- | --- | --- | --- |
| **ID** | **Diagnosis** | **CMT Pathogens** | **tNGS Infection** | **tNGS Pathogens** | **CT Infection** | **Treatment adjustment** | **Clinical outcome** | **Treatment adjustment-detail** |
| 1029240 | Pneumonia-mycoplasma pneumoniae infection | Rotavirus | 0 | NA | 1 | Unchanged | Improved | Unchanged |
| 1044828 | Mycoplasma pneumonia, gallbladder stones | Rotavirus | 1 | Mycoplasma pneumoniae; Rhinovirus type C | 1 | Unchanged | Improved | Unchanged |
| 1029192 | Bronchopneumonia | Rotavirus | 0 | NA | 1 | De-escalated | Improved | Discharge |
| 1029332 | Acute asthmatic bronchitis | Rotavirus | 1 | Rhinovirus | 1 | Escalated | Improved | Nebulization of interferon |
| 1009681 | Upper respiratory tract infection, pneumonia (recovery period) | NA | 1 | Influenza A virus H3N2 | 1 | Escalated | Improved | Others |
| 989903 | Severe pneumonia | NA | 1 | Human coronavirus OC43; Streptococcus pneumoniae | 1 | De-escalated | Improved | Discontinue medication |
| 1045873 | Severe pneumonia | NA | 1 | Human respiratory syncytial virus type A; Mycoplasma pneumoniae | 1 | Unchanged | Improved | Unchanged |
| 1045858 | Severe pneumonia | NA | 1 | Human adenovirus type 3; Influenza A virus H3N2; Mycoplasma pneumoniae | 1 | Escalated | Aggravated | Others |
| 1045357 | Severe pneumonia | NA | 1 | Mycoplasma pneumoniae | 1 | Unchanged | Unchanged | Unchanged |
| 1044953 | Severe pneumonia | NA | 1 | Rhinovirus | 1 | Escalated | Improved | Nebulization of interferon |
| 1044947 | Severe pneumonia | NA | 1 | Mycoplasma pneumoniae | 1 | Escalated | Improved | Medication added |
| 1044468 | Severe pneumonia | NA | 1 | Mycoplasma pneumoniae; Streptococcus pneumoniae | 1 | De-escalated | Improved | Reduce the amount |
| 1044259 | Severe pneumonia | NA | 1 | Mycoplasma pneumoniae | 1 | Escalated | Improved | Others |
| 1044123 | Severe pneumonia | NA | 1 | Mycoplasma pneumoniae; Rhinovirus type C; Streptococcus pneumoniae | 1 | Escalated | Improved | Medication added |
| 1043999 | Severe pneumonia | NA | 0 | NA | 1 | Unchanged | Improved | Unchanged |
| 1043691 | Severe pneumonia | NA | 1 | Human adenovirus type 3; Mycoplasma pneumoniae | 1 | Unchanged | Unchanged | Unchanged |
| 1043514 | Severe pneumonia | NA | 1 | Mycoplasma pneumoniae | 1 | De-escalated | Improved | Discontinue medication |
| 1043330 | Severe pneumonia | NA | 0 | NA | 1 | Unchanged | Improved | Unchanged |
| 1042446 | Severe pneumonia | NA | 1 | Human respiratory syncytial virus type B; Mycoplasma pneumoniae | 1 | Escalated | Improved | Prolong the duration of treatment |
| 1040925 | Severe pneumonia | NA | 1 | Mycoplasma pneumoniae | 1 | Unchanged | Improved | Unchanged |
| 1040212 | Severe pneumonia | NA | 1 | Mycoplasma pneumoniae | 1 | Unchanged | Improved | Unchanged |
| 1039945 | Severe pneumonia | NA | 1 | Mycoplasma pneumoniae | 1 | Escalated | Improved | Medications changed |
| 1039359 | Severe pneumonia | NA | 0 | NA | 1 | Unchanged | Improved | Unchanged |
| 1039034 | Severe pneumonia | NA | 1 | Mycoplasma pneumoniae | 1 | Escalated | Improved | Others |
| 1038384 | Severe pneumonia | NA | 1 | Human parainfluenza virus type 3; Mycoplasma pneumoniae; Rhinovirus type C | 1 | Unchanged | Improved | Unchanged |
| 1038344 | Severe pneumonia | NA | 1 | Mycoplasma pneumoniae; Epstein-Barr virus | 1 | Unchanged | Improved | Unchanged |
| 1038301 | Severe pneumonia | NA | 0 | NA | 1 | Unchanged | Improved | Unchanged |
| 1037848 | Severe pneumonia | NA | 1 | Human bocavirus type 1 | 1 | Unchanged | Improved | Unchanged |
| 1035061 | Severe pneumonia | NA | 1 | Herpes simplex virus type 1 | 1 | Unchanged | Improved | Unchanged |
| 1034117 | Severe pneumonia | NA | 1 | Human metapneumovirus; Haemophilus influenzae | 1 | De-escalated | Improved | Discontinue medication |
| 1033746 | Severe pneumonia | NA | 1 | Human parainfluenza virus type 3; Haemophilus influenzae | 1 | Unchanged | Improved | Unchanged |
| 1033660 | Severe pneumonia | NA | 1 | Human bocavirus type 1 | 1 | Unchanged | Improved | Unchanged |
| 1032859 | Severe pneumonia | NA | 1 | Streptococcus pyogenes; Enterovirus group B; Rhinovirus type B; Streptococcus pneumoniae; Haemophilus influenzae | 1 | De-escalated | Improved | Reduce the amount |
| 1032423 | Severe pneumonia | NA | 1 | Human respiratory syncytial virus type A; Moraxella catarrhalis | 1 | Escalated | Improved | Medication added |
| 1031840 | Severe pneumonia | NA | 1 | Mycoplasma pneumoniae | 1 | Escalated | Improved | Medication added |
| 1031308 | Severe pneumonia | NA | 1 | Human bocavirus type 1 | 1 | De-escalated | Improved | Discontinue medication |
| 1030918 | Severe pneumonia | NA | 1 | Human parainfluenza virus type 3; Haemophilus influenzae | 1 | Unchanged | Improved | Unchanged |
| 1028991 | Severe pneumonia | NA | 1 | Human respiratory syncytial virus type B | 1 | Unchanged | Improved | Unchanged |
| 1028978 | Severe pneumonia | NA | 1 | Human bocavirus type 1 | 1 | Unchanged | Aggravated | Unchanged |
| 1027215 | Severe pneumonia | NA | 1 | Human respiratory syncytial virus type A; Rhinovirus type C; Streptococcus pneumoniae; Cytomegalovirus; Moraxella catarrhalis | 1 | Unchanged | Aggravated | Unchanged |
| 1027131 | Severe pneumonia | NA | 1 | Human respiratory syncytial virus type A; Human bocavirus type 1; Herpes simplex virus type 1 | 1 | Escalated | Improved | Medications changed |
| 1033574 | Refractory Mycoplasma Pneumonia | NA | 1 | Mycoplasma pneumoniae | 1 | Escalated | Aggravated | Medication added |
| 1041964 | Pneumonia-mycoplasma pneumoniae infection | NA | 1 | Influenza A virus H3N2 | 1 | Escalated | Improved | Medication added |
| 1041921 | Pneumonia-mycoplasma pneumoniae infection | NA | 0 | NA | 1 | Unchanged | Improved | Unchanged |
| 1041820 | Pneumonia-mycoplasma pneumoniae infection | NA | 1 | Human bocavirus type 1 | 1 | Escalated | Improved | Medication added |
| 1041615 | Pneumonia-mycoplasma pneumoniae infection | NA | 1 | Mycoplasma pneumoniae; Rhinovirus type A | 1 | De-escalated | Improved | Reduce the amount |
| 1041507 | Pneumonia-mycoplasma pneumoniae infection | NA | 1 | Rhinovirus type C | 1 | Unchanged | Improved | Unchanged |
| 1041424 | Pneumonia-mycoplasma pneumoniae infection | NA | 1 | Mycoplasma pneumoniae | 1 | Unchanged | Improved | Unchanged |
| 1041221 | Pneumonia-mycoplasma pneumoniae infection | NA | 1 | Influenza A virus H3N2 | 1 | Unchanged | Improved | Unchanged |
| 1041106 | Pneumonia-mycoplasma pneumoniae infection | NA | 1 | Rhinovirus type B; Haemophilus influenzae | 1 | Escalated | Improved | Medication added |
| 1040832 | Pneumonia-mycoplasma pneumoniae infection | NA | 1 | Mycoplasma pneumoniae | 1 | Unchanged | Improved | Unchanged |
| 1040407 | Pneumonia-mycoplasma pneumoniae infection | NA | 1 | Mycoplasma pneumoniae; Rhinovirus type B | 1 | Unchanged | Improved | Unchanged |
| 1040308 | Pneumonia-mycoplasma pneumoniae infection | NA | 1 | Human respiratory syncytial virus type B; Epstein-Barr virus | 1 | Unchanged | Improved | Unchanged |
| 1040153 | Pneumonia-mycoplasma pneumoniae infection | NA | 1 | Human metapneumovirus; Mycoplasma pneumoniae; Bordetella pertussis | 1 | Escalated | Improved | Medication added |
| 1039767 | Pneumonia-mycoplasma pneumoniae infection | NA | 1 | Mycoplasma pneumoniae | 1 | Escalated | Improved | Medications changed |
| 1039333 | Pneumonia-mycoplasma pneumoniae infection | NA | 1 | Human metapneumovirus; Influenza A virus H3N2 | 1 | Escalated | Improved | Medication added |
| 1039198 | Pneumonia-mycoplasma pneumoniae infection | NA | 1 | SARS-CoV-2 omicron XBB; Mycoplasma pneumoniae | 1 | Unchanged | Improved | Unchanged |
| 1038931 | Pneumonia-mycoplasma pneumoniae infection | NA | 1 | Human respiratory syncytial virus type B | 1 | Unchanged | Improved | Unchanged |
| 1038527 | Pneumonia-mycoplasma pneumoniae infection | NA | 1 | Rhinovirus type B | 1 | Unchanged | Improved | Unchanged |
| 1034828 | Pneumonia-mycoplasma pneumoniae infection | NA | 1 | Rhinovirus type A; Bordetella pertussis; Haemophilus influenzae | 1 | Unchanged | Improved | Unchanged |
| 1034789 | Pneumonia-mycoplasma pneumoniae infection | NA | 1 | Human parainfluenza virus type 4; Human parainfluenza virus type 1 | 1 | Unchanged | Improved | Unchanged |
| 1033849 | Pneumonia-mycoplasma pneumoniae infection | NA | 1 | Mycoplasma pneumoniae; Rhinovirus type A | 1 | Unchanged | Improved | Unchanged |
| 1033840 | Pneumonia-mycoplasma pneumoniae infection | NA | 0 | NA | 1 | Unchanged | Improved | Unchanged |
| 1033440 | Pneumonia-mycoplasma pneumoniae infection | NA | 1 | Human parainfluenza virus type 3 | 1 | Unchanged | Improved | Unchanged |
| 1033418 | Pneumonia-mycoplasma pneumoniae infection | NA | 1 | Human parainfluenza virus type 3; Rhinovirus type A; Streptococcus pneumoniae | 1 | Escalated | Improved | Medication added |
| 1033347 | Pneumonia-mycoplasma pneumoniae infection | NA | 1 | Human parainfluenza virus type 1 | 1 | De-escalated | Improved | Reduce the amount |
| 1033001 | Pneumonia-mycoplasma pneumoniae infection | NA | 1 | Human metapneumovirus | 0 | Unchanged | Improved | Unchanged |
| 1032817 | Pneumonia-mycoplasma pneumoniae infection | NA | 1 | Human adenovirus type 5; Human parainfluenza virus type 3; Rhinovirus type A; Haemophilus influenzae | 0 | Escalated | Improved | Medication added |
| 1032287 | Pneumonia-mycoplasma pneumoniae infection | NA | 1 | Human metapneumovirus | 1 | Unchanged | Improved | Unchanged |
| 1031663 | Pneumonia-mycoplasma pneumoniae infection | NA | 1 | Human parainfluenza virus type 3 | 0 | De-escalated | Improved | Discharge |
| 1029651 | Pneumonia-mycoplasma pneumoniae infection | NA | 1 | Rhinovirus type A | 0 | Escalated | Improved | Medication added |
| 1029164 | Pneumonia-mycoplasma pneumoniae infection | NA | 1 | Human respiratory syncytial virus type A; Streptococcus pneumoniae | 1 | Escalated | Improved | Medication added |
| 1029144 | Pneumonia-mycoplasma pneumoniae infection | NA | 1 | Human respiratory syncytial virus type A; Human coronavirus OC43 | 1 | De-escalated | Improved | Discontinue medication |
| 1028301 | Pneumonia-mycoplasma pneumoniae infection | NA | 1 | Human coronavirus OC43 | 0 | Unchanged | Improved | Unchanged |
| 1026467 | Pneumonia-mycoplasma pneumoniae infection | NA | 1 | Human respiratory syncytial virus type A | 1 | Unchanged | Improved | Unchanged |
| 1020887 | Pneumonia-mycoplasma pneumoniae infection | NA | 1 | Human parainfluenza virus type 3 | 0 | De-escalated | Improved | Discharge |
| 1007704 | Pneumonia, whooping cough, rhinitis | NA | 1 | Rhinovirus type A; Bordetella pertussis | 1 | Escalated | Improved | Others |
| 1019640 | Pneumonia, purulent tonsillitis, herpangina | NA | 1 | Coxsackievirus type A6 | 1 | De-escalated | Improved | Discharge |
| 1026394 | Pneumonia, otitis media, systemic inflammatory response syndrome | NA | 1 | Herpes simplex virus type 1; Rhinovirus type A | 1 | De-escalated | Improved | Discontinue medication |
| 1010239 | Pneumonia, laryngitis | NA | 1 | Human respiratory syncytial virus type A | 1 | De-escalated | Improved | Discontinue medication |
| 1025631 | Pneumonia, febrile convulsions | NA | 1 | Human respiratory syncytial virus type A | 1 | De-escalated | Improved | Discharge |
| 1026294 | Pneumonia, enteritis | NA | 1 | Human respiratory syncytial virus type A | 1 | De-escalated | Improved | Discharge |
| 993513 | Pneumonia | NA | 1 | Mycoplasma pneumoniae; Herpes simplex virus type 1 | 1 | Escalated | Improved | Prolong the duration of treatment |
| 992734 | Pneumonia | NA | 1 | Human parainfluenza virus type 3 | 1 | Escalated | Improved | Medication added |
| 992215 | Pneumonia | NA | 1 | Human adenovirus type 3 | 1 | De-escalated | Improved | Discontinue medication |
| 990144 | Pneumonia | NA | 1 | Influenza A virus H3N2 | 1 | Unchanged | Improved | Unchanged |
| 989615 | Pneumonia | NA | 1 | Influenza A virus H3N2 | 1 | Escalated | Improved | Medication added |
| 988570 | Pneumonia | NA | 1 | Human metapneumovirus | 1 | De-escalated | Improved | Discontinue medication |
| 987826 | Pneumonia | NA | 1 | Mycoplasma pneumoniae | 1 | De-escalated | Improved | Discontinue medication |
| 987246 | Pneumonia | NA | 1 | Coxsackievirus type A6; Influenza C virus; Moraxella catarrhalis | 1 | De-escalated | Improved | Discontinue medication |
| 987085 | Pneumonia | NA | 1 | Human metapneumovirus | 1 | De-escalated | Improved | Others |
| 985612 | Pneumonia | NA | 1 | Human adenovirus type 3 | 1 | Escalated | Improved | Medication added |
| 985342 | Pneumonia | NA | 1 | Rhinovirus type A | NA | Escalated | Improved | Medication added |
| 983914 | Pneumonia | NA | 1 | Human metapneumovirus | 1 | De-escalated | Improved | Discontinue medication |
| 983245 | Pneumonia | NA | 1 | Human parainfluenza virus type 3; Haemophilus influenzae | 1 | De-escalated | Improved | Discontinue medication |
| 982003 | Pneumonia | NA | 1 | Mycoplasma pneumoniae | 1 | Escalated | Improved | Medications changed |
| 981329 | Pneumonia | NA | 0 | NA | 1 | De-escalated | Improved | Discontinue medication |
| 981050 | Pneumonia | NA | 1 | Mycoplasma pneumoniae | 1 | De-escalated | Improved | Reduce the amount |
| 980802 | Pneumonia | NA | 1 | Human parainfluenza virus type 4; Human metapneumovirus | 1 | De-escalated | Improved | Discontinue medication |
| 980125 | Pneumonia | NA | 1 | Human parainfluenza virus type 1; Rhinovirus type A | 1 | Escalated | Improved | Medication added |
| 978186 | Pneumonia | NA | 1 | Human metapneumovirus | 1 | De-escalated | Improved | Discontinue medication |
| 976749 | Pneumonia | NA | 1 | Herpes simplex virus type 1 | 1 | De-escalated | Improved | Discontinue medication |
| 976447 | Pneumonia | NA | 1 | Human adenovirus type 3; Human parainfluenza virus type 1 | 1 | De-escalated | Improved | Discontinue medication |
| 975356 | Pneumonia | NA | 1 | Mycoplasma pneumoniae | 1 | Escalated | Improved | Medication added |
| 972926 | Pneumonia | NA | 1 | Streptococcus agalactiae; Human metapneumovirus; Influenza A virus; Mycoplasma pneumoniae | 1 | De-escalated | Improved | Discontinue medication |
| 972682 | Pneumonia | NA | 1 | Human metapneumovirus | 1 | De-escalated | Improved | Others |
| 970345 | Pneumonia | NA | 1 | Human respiratory syncytial virus type A; Streptococcus pneumoniae | 1 | De-escalated | Improved | Discontinue medication |
| 964386 | Pneumonia | NA | 1 | Human parainfluenza virus type 3; Coxsackievirus type A10 | 1 | Unchanged | Improved | Unchanged |
| 959630 | Pneumonia | NA | 1 | Human metapneumovirus | 1 | Escalated | Improved | Medication added |
| 956238 | Pneumonia | NA | 1 | Human parainfluenza virus type 3 | 1 | De-escalated | Improved | Discontinue medication |
| 955886 | Pneumonia | NA | 1 | Rhinovirus type C; Epstein-Barr virus | 1 | De-escalated | Improved | Others |
| 953013 | Pneumonia | NA | 1 | Mycoplasma pneumoniae | 1 | De-escalated | Improved | Discontinue medication |
| 948385 | Pneumonia | NA | 1 | Human respiratory syncytial virus type A; Coxsackievirus type A10; Mycoplasma pneumoniae; Streptococcus pneumoniae | NA | De-escalated | Improved | Discontinue medication |
| 925535 | Pneumonia | NA | 1 | Human adenovirus type 3 | 1 | Escalated | Improved | Nebulization of interferon |
| 925496 | Pneumonia | NA | 1 | Rhinovirus type A | 1 | Escalated | Improved | Others |
| 919935 | Pneumonia | NA | 1 | Human parainfluenza virus type 1; Epstein-Barr virus | 1 | Escalated | Improved | Medication added |
| 917824 | Pneumonia | NA | 1 | Mycoplasma pneumoniae | 1 | Escalated | Improved | Prolong the duration of treatment |
| 917695 | Pneumonia | NA | 1 | Human parainfluenza virus type 1; Mycoplasma pneumoniae | 1 | De-escalated | Improved | Discontinue medication |
| 916484 | Pneumonia | NA | 1 | Human respiratory syncytial virus type B; Mycoplasma pneumoniae; Bordetella pertussis | 1 | Escalated | Improved | Others |
| 909410 | Pneumonia | NA | 1 | SARS-CoV-2 omicron XBB; Rhinovirus; Bordetella pertussis | 1 | Escalated | Improved | Medication added |
| 908645 | Pneumonia | NA | 1 | Human parainfluenza virus type 4; Herpes simplex virus type 1; Haemophilus influenzae | 1 | De-escalated | Improved | Discontinue medication |
| 898825 | Pneumonia | NA | 1 | Human adenovirus type 3 | 1 | Unchanged | Improved | Unchanged |
| 897777 | Pneumonia | NA | 1 | Human metapneumovirus | 1 | Escalated | Improved | Medication added |
| 885247 | Pneumonia | NA | 0 | NA | NA | Unchanged | Improved | Unchanged |
| 882943 | Pneumonia | NA | 1 | Rhinovirus type C; Epstein-Barr virus; Haemophilus influenzae | 1 | Escalated | Improved | Nebulization of interferon |
| 879128 | Pneumonia | NA | 1 | Rhinovirus | 1 | De-escalated | Improved | Discontinue medication |
| 874174 | Pneumonia | NA | 1 | Human metapneumovirus | 1 | Unchanged | Improved | Unchanged |
| 853311 | Pneumonia | NA | 1 | Mycoplasma pneumoniae; Rhinovirus type C | 1 | Unchanged | Unchanged | Unchanged |
| 831862 | Pneumonia | NA | 1 | Mycoplasma pneumoniae | 1 | De-escalated | Improved | Others |
| 813339 | Pneumonia | NA | 1 | Influenza A virus; Epstein-Barr virus | 1 | De-escalated | Improved | Discharge |
| 1045998 | Pneumonia | NA | 1 | Mycoplasma pneumoniae | 1 | Escalated | Improved | Prolong the duration of treatment |
| 1045790 | Pneumonia | NA | 1 | Human adenovirus type 3; Mycoplasma pneumoniae | 1 | Escalated | Improved | Medication added |
| 1045763 | Pneumonia | NA | 1 | Mycoplasma pneumoniae | 1 | Escalated | Improved | Prolong the duration of treatment |
| 1045660 | Pneumonia | NA | 1 | Human adenovirus type 3 | 1 | De-escalated | Improved | Discontinue medication |
| 1045601 | Pneumonia | NA | 1 | Human metapneumovirus; Cytomegalovirus | NA | Escalated | Improved | Others |
| 1045593 | Pneumonia | NA | 1 | Human adenovirus type 3 | 1 | Escalated | Improved | Medication added |
| 1045583 | Pneumonia | NA | 1 | Human adenovirus type 3 | 1 | Escalated | Improved | Nebulization of interferon |
| 1045575 | Pneumonia | NA | 1 | Human adenovirus type 3; Mycoplasma pneumoniae | 1 | Escalated | Improved | Nebulization of interferon |
| 1045458 | Pneumonia | NA | 1 | Human respiratory syncytial virus type B; Mycoplasma pneumoniae | 1 | Escalated | Improved | Medication added |
| 1045278 | Pneumonia | NA | 1 | Mycoplasma pneumoniae | 1 | Escalated | Improved | Medication added |
| 1045268 | Pneumonia | NA | 1 | Influenza A virus | 1 | De-escalated | Improved | Discontinue medication |
| 1045248 | Pneumonia | NA | 1 | Influenza A virus H3N2 | 1 | Unchanged | Improved | Unchanged |
| 1045236 | Pneumonia | NA | 1 | Human adenovirus type 3; Mycoplasma pneumoniae | 1 | Escalated | Improved | Nebulization of interferon |
| 1045209 | Pneumonia | NA | 1 | Human adenovirus type 3; Epstein-Barr virus | 1 | Escalated | Improved | Medication added |
| 1045170 | Pneumonia | NA | 1 | Human adenovirus type 3 | 1 | Unchanged | Improved | Unchanged |
| 1045144 | Pneumonia | NA | 1 | Human respiratory syncytial virus type B | 1 | Escalated | Improved | Nebulization of interferon |
| 1045134 | Pneumonia | NA | 1 | Human respiratory syncytial virus type B; Mycoplasma pneumoniae | 1 | Escalated | Improved | Medication added |
| 1045033 | Pneumonia | NA | 1 | Human respiratory syncytial virus type B; Human coronavirus 229E | 1 | Escalated | Improved | Medication added |
| 1044975 | Pneumonia | NA | 1 | Human metapneumovirus | 1 | Escalated | Improved | Nebulization of interferon |
| 1044939 | Pneumonia | NA | 1 | Human metapneumovirus; Epstein-Barr virus | 1 | De-escalated | Improved | Discontinue medication |
| 1044841 | Pneumonia | NA | 1 | Human adenovirus type 3 | 1 | De-escalated | Improved | Discontinue medication |
| 1044824 | Pneumonia | NA | 1 | Mycoplasma pneumoniae | 1 | Escalated | Improved | Medication added |
| 1044813 | Pneumonia | NA | 0 | NA | 1 | Unchanged | Improved | Unchanged |
| 1044742 | Pneumonia | NA | 1 | Mycoplasma pneumoniae; Influenza C virus; Haemophilus influenzae | 1 | Escalated | Improved | Medication added |
| 1044728 | Pneumonia | NA | 1 | Influenza A virus; Haemophilus influenzae; Moraxella catarrhalis | 0 | De-escalated | Improved | Discontinue medication |
| 1044721 | Pneumonia | NA | 1 | Human adenovirus type 3; Influenza A virus | 1 | Escalated | Improved | Medication added |
| 1044651 | Pneumonia | NA | 1 | Influenza A virus H3N2 | 0 | Unchanged | Aggravated | Unchanged |
| 1044621 | Pneumonia | NA | 1 | Mycoplasma pneumoniae | 1 | Escalated | Improved | Prolong the duration of treatment |
| 1044528 | Pneumonia | NA | 1 | Rhinovirus type A; Bordetella pertussis | 1 | Unchanged | Improved | Unchanged |
| 1044465 | Pneumonia | NA | 1 | Human respiratory syncytial virus type B | 1 | Unchanged | Improved | Unchanged |
| 1044457 | Pneumonia | NA | 1 | Human respiratory syncytial virus type B; Streptococcus pneumoniae | 1 | De-escalated | Improved | Discontinue medication |
| 1044445 | Pneumonia | NA | 1 | Mycoplasma pneumoniae | 1 | De-escalated | Improved | Reduce the amount |
| 1044338 | Pneumonia | NA | 1 | Human metapneumovirus; Streptococcus pyogenes | 1 | De-escalated | Improved | Discontinue medication |
| 1044307 | Pneumonia | NA | 1 | Human metapneumovirus; Haemophilus influenzae | 1 | De-escalated | Improved | Reduce the amount |
| 1044236 | Pneumonia | NA | 1 | Human adenovirus type 3; Mycoplasma pneumoniae | 1 | Escalated | Improved | Medication added |
| 1044184 | Pneumonia | NA | 1 | Influenza A virus H3N2 | 1 | Escalated | Improved | Medication added |
| 1044136 | Pneumonia | NA | 1 | Bordetella pertussis | 1 | Escalated | Improved | Medication added |
| 1044121 | Pneumonia | NA | 1 | Mycoplasma pneumoniae | 1 | Unchanged | Improved | Unchanged |
| 1044039 | Pneumonia | NA | 1 | Human respiratory syncytial virus type A; Streptococcus pneumoniae | 1 | De-escalated | Improved | Discontinue medication |
| 1044035 | Pneumonia | NA | 1 | Mycoplasma pneumoniae; Rhinovirus type B; Epstein-Barr virus | 1 | Escalated | Improved | Medication added |
| 1043989 | Pneumonia | NA | 1 | Mycoplasma pneumoniae | 1 | Escalated | Improved | Prolong the duration of treatment |
| 1043914 | Pneumonia | NA | 1 | Mycoplasma pneumoniae | 1 | Escalated | Improved | Prolong the duration of treatment |
| 1043894 | Pneumonia | NA | 1 | Mycoplasma pneumoniae | 1 | De-escalated | Improved | Discontinue medication |
| 1043878 | Pneumonia | NA | 1 | Human respiratory syncytial virus type B; Influenza A virus H3N2; Mycoplasma pneumoniae; Herpes simplex virus type 1 | 1 | Escalated | Improved | Medication added |
| 1043877 | Pneumonia | NA | 1 | Human metapneumovirus; Mycoplasma pneumoniae; Rhinovirus | 1 | De-escalated | Improved | Discontinue medication |
| 1043801 | Pneumonia | NA | 1 | Influenza A virus H3N2; Haemophilus influenzae | 1 | De-escalated | Improved | Discontinue medication |
| 1043778 | Pneumonia | NA | 1 | Mycoplasma pneumoniae | 1 | Escalated | Improved | Medication added |
| 1043774 | Pneumonia | NA | 1 | Influenza A virus H3N2 | 1 | De-escalated | Improved | Discontinue medication |
| 1043772 | Pneumonia | NA | 1 | Influenza A virus H3N2 | 1 | De-escalated | Improved | Discontinue medication |
| 1043727 | Pneumonia | NA | 1 | Mycoplasma pneumoniae; Rhinovirus type C | 1 | De-escalated | Improved | Reduce the amount |
| 1043656 | Pneumonia | NA | 1 | Influenza A virus | 1 | Unchanged | Improved | Unchanged |
| 1043653 | Pneumonia | NA | 1 | Cytomegalovirus | 0 | Unchanged | Improved | Unchanged |
| 1043588 | Pneumonia | NA | 1 | Rhinovirus type B | 1 | Escalated | Improved | Others |
| 1043583 | Pneumonia | NA | 1 | Influenza A virus; Epstein-Barr virus; Haemophilus influenzae | 1 | Unchanged | Improved | Unchanged |
| 1043582 | Pneumonia | NA | 1 | Influenza A virus H3N2; Mycoplasma pneumoniae; Bordetella pertussis; Streptococcus pneumoniae | 1 | Escalated | Improved | Prolong the duration of treatment |
| 1043541 | Pneumonia | NA | 1 | Influenza A virus H3N2; Bordetella pertussis | 1 | Escalated | Improved | Prolong the duration of treatment |
| 1043536 | Pneumonia | NA | 0 | NA | 1 | De-escalated | Improved | Others |
| 1043530 | Pneumonia | NA | 1 | Mycoplasma pneumoniae | 1 | Escalated | Improved | Prolong the duration of treatment |
| 1043528 | Pneumonia | NA | 1 | Mycoplasma pneumoniae | 1 | De-escalated | Improved | Discontinue medication |
| 1043524 | Pneumonia | NA | 1 | Influenza A virus | 1 | Escalated | Improved | Medication added |
| 1043518 | Pneumonia | NA | 1 | Influenza A virus H3N2; Rhinovirus type B; Epstein-Barr virus | 1 | Escalated | Improved | Medication added |
| 1043517 | Pneumonia | NA | 1 | Human adenovirus type 5 | 1 | De-escalated | Improved | Discharge |
| 1043516 | Pneumonia | NA | 1 | Mycoplasma pneumoniae | 1 | De-escalated | Improved | Discontinue medication |
| 1043511 | Pneumonia | NA | 1 | Human adenovirus type 3; Mycoplasma pneumoniae | 1 | Escalated | Improved | Others |
| 1043506 | Pneumonia | NA | 1 | Human adenovirus type 3; Epstein-Barr virus | 1 | Escalated | Improved | Medication added |
| 1043433 | Pneumonia | NA | 1 | Mycoplasma pneumoniae; Rhinovirus type B | 1 | De-escalated | Improved | Discontinue medication |
| 1043394 | Pneumonia | NA | 1 | Influenza A virus H3N2; Bordetella pertussis; Streptococcus pneumoniae | 1 | Escalated | Improved | Medication added |
| 1043313 | Pneumonia | NA | 1 | Influenza A virus H3N2; Haemophilus influenzae | 0 | Escalated | Improved | Medication added |
| 1043303 | Pneumonia | NA | 1 | Influenza A virus H3N2 | 1 | Escalated | Improved | Medication added |
| 1043243 | Pneumonia | NA | 1 | Influenza A virus H3N2; Mycoplasma pneumoniae | 1 | Escalated | Improved | Medication added |
| 1043217 | Pneumonia | NA | 1 | Mycoplasma pneumoniae | 1 | Escalated | Improved | Medication added |
| 1043211 | Pneumonia | NA | 0 | NA | 1 | Escalated | Improved | Medication added |
| 1043193 | Pneumonia | NA | 1 | Influenza A virus H3N2; Mycoplasma pneumoniae | 1 | Escalated | Improved | Medication added |
| 1043188 | Pneumonia | NA | 1 | Influenza A virus H3N2; Mycoplasma pneumoniae; Rhinovirus type B | 1 | Escalated | Improved | Medication added |
| 1043140 | Pneumonia | NA | 1 | Mycoplasma pneumoniae | 1 | Escalated | Improved | Others |
| 1043132 | Pneumonia | NA | 1 | Influenza A virus H3N2; Epstein-Barr virus | 1 | Escalated | Improved | Medication added |
| 1043116 | Pneumonia | NA | 1 | Mycoplasma pneumoniae; Bordetella pertussis | 1 | Escalated | Improved | Medication added |
| 1043113 | Pneumonia | NA | 1 | Mycoplasma pneumoniae; Influenza C virus; Rhinovirus type C | 1 | Escalated | Improved | Medication added |
| 1043105 | Pneumonia | NA | 1 | Mycoplasma pneumoniae | 1 | Escalated | Improved | Medication added |
| 1043047 | Pneumonia | NA | 1 | Mycoplasma pneumoniae | 1 | De-escalated | Improved | Discontinue medication |
| 1043006 | Pneumonia | NA | 1 | Human adenovirus type 3; Mycoplasma pneumoniae | NA | De-escalated | Improved | Reduce the amount |
| 1042990 | Pneumonia | NA | 1 | Influenza A virus | 1 | Escalated | Improved | Medication added |
| 1042957 | Pneumonia | NA | 1 | Human adenovirus type 3; Rhinovirus type C; Haemophilus influenzae | NA | Escalated | Improved | Medication added |
| 1042930 | Pneumonia | NA | 1 | Influenza A virus H3N2; Rhinovirus type C; Bordetella pertussis | 1 | Escalated | Improved | Medication added |
| 1042893 | Pneumonia | NA | 1 | Mycoplasma pneumoniae | 1 | De-escalated | Improved | Discontinue medication |
| 1042888 | Pneumonia | NA | 1 | Human metapneumovirus; Mycoplasma pneumoniae; Moraxella catarrhalis; Haemophilus influenzae | 1 | Unchanged | Improved | Unchanged |
| 1042834 | Pneumonia | NA | 1 | Influenza A virus H3N2; Mycoplasma pneumoniae; Streptococcus pneumoniae | 1 | De-escalated | Improved | Discontinue medication |
| 1042830 | Pneumonia | NA | 1 | Human respiratory syncytial virus type B; Human bocavirus type 1 | 1 | De-escalated | Improved | Discontinue medication |
| 1042806 | Pneumonia | NA | 1 | Human bocavirus type 1; Haemophilus influenzae | 1 | De-escalated | Improved | Reduce the amount |
| 1042805 | Pneumonia | NA | 1 | Mycoplasma pneumoniae | 1 | De-escalated | Improved | Discontinue medication |
| 1042799 | Pneumonia | NA | 1 | Mycoplasma pneumoniae | 1 | De-escalated | Improved | Discontinue medication |
| 1042697 | Pneumonia | NA | 1 | Human adenovirus type 3; Influenza A virus | 1 | De-escalated | Improved | Discontinue medication |
| 1042609 | Pneumonia | NA | 1 | Influenza A virus | 1 | De-escalated | Improved | Discontinue medication |
| 1042589 | Pneumonia | NA | 1 | Human parainfluenza virus type 1; Rhinovirus type A | 1 | De-escalated | Improved | Reduce the amount |
| 1042562 | Pneumonia | NA | 1 | Human respiratory syncytial virus type B | 1 | Escalated | Improved | Medication added |
| 1042474 | Pneumonia | NA | 1 | Mycoplasma pneumoniae | 1 | De-escalated | Improved | Reduce the amount |
| 1042358 | Pneumonia | NA | 1 | Mycoplasma pneumoniae | 1 | De-escalated | Improved | Discontinue medication |
| 1042279 | Pneumonia | NA | 1 | Mycoplasma pneumoniae | 1 | De-escalated | Improved | Discontinue medication |
| 1042263 | Pneumonia | NA | 0 | NA | 1 | Escalated | Improved | Medication added |
| 1042176 | Pneumonia | NA | 1 | Influenza A virus H3N2 | 1 | Escalated | Improved | Medication added |
| 1042173 | Pneumonia | NA | 0 | NA | 1 | De-escalated | Improved | Discontinue medication |
| 1042154 | Pneumonia | NA | 1 | Mycoplasma pneumoniae; Rhinovirus type A; Bordetella pertussis | 1 | De-escalated | Improved | Reduce the amount |
| 1041962 | Pneumonia | NA | 1 | Human adenovirus type 3; Rhinovirus type B; Haemophilus influenzae | 1 | Escalated | Improved | Medication added |
| 1041623 | Pneumonia | NA | 1 | Mycoplasma pneumoniae; Rhinovirus type B | 1 | De-escalated | Improved | Reduce the amount |
| 1041531 | Pneumonia | NA | 1 | Influenza A virus H3N2 | 1 | Escalated | Improved | Medication added |
| 1041525 | Pneumonia | NA | 1 | Influenza A virus; Rhinovirus type C; Streptococcus pneumoniae | 1 | Escalated | Improved | Medication added |
| 1041303 | Pneumonia | NA | 1 | Human adenovirus type 3; Human metapneumovirus; Moraxella catarrhalis; Streptococcus pneumoniae | 1 | Escalated | Improved | Medication added |
| 1041287 | Pneumonia | NA | 1 | Human metapneumovirus | 1 | Unchanged | Improved | Unchanged |
| 1041059 | Pneumonia | NA | 1 | Influenza A virus H3N2 | 1 | Escalated | Improved | Others |
| 1041009 | Pneumonia | NA | 1 | Influenza A virus; Mycoplasma pneumoniae | 1 | Unchanged | Improved | Unchanged |
| 1041000 | Pneumonia | NA | 1 | Influenza A virus H3N2 | 1 | Escalated | Improved | Others |
| 1040988 | Pneumonia | NA | 1 | Human adenovirus type 3; Herpes simplex virus type 1; Epstein-Barr virus; Moraxella catarrhalis; Haemophilus influenzae | 1 | De-escalated | Improved | Reduce the amount |
| 1040687 | Pneumonia | NA | 1 | Human metapneumovirus | 1 | Escalated | Improved | Medication added |
| 1040396 | Pneumonia | NA | 1 | Rhinovirus type A; Haemophilus influenzae; Cytomegalovirus | 1 | Unchanged | Improved | Unchanged |
| 1040294 | Pneumonia | NA | 1 | Mycoplasma pneumoniae | 1 | Unchanged | Improved | Unchanged |
| 1040164 | Pneumonia | NA | 1 | Mycoplasma pneumoniae; Influenza C virus | 1 | Unchanged | Improved | Unchanged |
| 1040073 | Pneumonia | NA | 1 | Mycoplasma pneumoniae | 1 | Unchanged | Improved | Unchanged |
| 1039977 | Pneumonia | NA | 1 | Mycoplasma pneumoniae | 1 | Escalated | Improved | Medications changed |
| 1039706 | Pneumonia | NA | 1 | Human bocavirus type 1; Haemophilus influenzae | 1 | Escalated | Improved | Medication added |
| 1039369 | Pneumonia | NA | 1 | Bordetella pertussis | 1 | Unchanged | Improved | Unchanged |
| 1039120 | Pneumonia | NA | 1 | Mycoplasma pneumoniae; Epstein-Barr virus | 1 | Unchanged | Improved | Unchanged |
| 1039049 | Pneumonia | NA | 1 | Human respiratory syncytial virus type B | 1 | Unchanged | Improved | Unchanged |
| 1039045 | Pneumonia | NA | 1 | Human respiratory syncytial virus type A; Coxsackievirus type A6; Haemophilus influenzae | 1 | Escalated | Improved | Medication added |
| 1038695 | Pneumonia | NA | 1 | Mycoplasma pneumoniae | 1 | Unchanged | Improved | Unchanged |
| 1038387 | Pneumonia | NA | 0 | NA | 1 | Unchanged | Improved | Unchanged |
| 1038296 | Pneumonia | NA | 1 | Bordetella pertussis; Epstein-Barr virus; Haemophilus influenzae | 1 | Escalated | Improved | Medication added |
| 1038295 | Pneumonia | NA | 1 | Haemophilus influenzae | 1 | Unchanged | Improved | Unchanged |
| 1038130 | Pneumonia | NA | 1 | Mycoplasma pneumoniae | 1 | Unchanged | Improved | Unchanged |
| 1038028 | Pneumonia | NA | 1 | Mycoplasma pneumoniae | 1 | Unchanged | Improved | Unchanged |
| 1037883 | Pneumonia | NA | 1 | Human parainfluenza virus type 1; Human bocavirus type 1; Streptococcus pneumoniae | 1 | Unchanged | Improved | Unchanged |
| 1037839 | Pneumonia | NA | 1 | Human respiratory syncytial virus type A | 1 | Unchanged | Improved | Unchanged |
| 1037740 | Pneumonia | NA | 1 | SARS-CoV-2 omicron XBB; Mycoplasma pneumoniae | 1 | De-escalated | Improved | Reduce the amount |
| 1037293 | Pneumonia | NA | 1 | Human metapneumovirus; Moraxella catarrhalis | 1 | Unchanged | Improved | Unchanged |
| 1037082 | Pneumonia | NA | 1 | Mycoplasma pneumoniae | 1 | Unchanged | Improved | Unchanged |
| 1036765 | Pneumonia | NA | 1 | Mycoplasma pneumoniae | 1 | Escalated | Improved | Medication added |
| 1036547 | Pneumonia | NA | 1 | SARS-CoV-2 omicron XBB; Mycoplasma pneumoniae | 1 | Unchanged | Improved | Unchanged |
| 1036491 | Pneumonia | NA | 1 | Human bocavirus type 1 | 1 | Unchanged | Improved | Unchanged |
| 1036450 | Pneumonia | NA | 0 | NA | 1 | Unchanged | Improved | Unchanged |
| 1036239 | Pneumonia | NA | 1 | Rhinovirus type A; Haemophilus influenzae | 1 | Unchanged | Improved | Unchanged |
| 1035403 | Pneumonia | NA | 1 | Human bocavirus type 1 | 1 | Unchanged | Improved | Unchanged |
| 1035292 | Pneumonia | NA | 1 | Bordetella pertussis | 1 | De-escalated | Improved | Discontinue medication |
| 1035167 | Pneumonia | NA | 1 | Human metapneumovirus; Streptococcus pneumoniae | 1 | Unchanged | Improved | Unchanged |
| 1034957 | Pneumonia | NA | 1 | SARS-CoV-2 omicron XBB; Bordetella pertussis | 1 | Unchanged | Improved | Unchanged |
| 1034033 | Pneumonia | NA | 1 | Human parainfluenza virus type 4 | 1 | Unchanged | Improved | Unchanged |
| 1034011 | Pneumonia | NA | 1 | Human parainfluenza virus type 3 | 1 | Escalated | Improved | Medication added |
| 1033932 | Pneumonia | NA | 1 | Human metapneumovirus; Streptococcus pneumoniae | 1 | Unchanged | Improved | Unchanged |
| 1033846 | Pneumonia | NA | 1 | Human metapneumovirus; Rhinovirus type C; Moraxella catarrhalis; Streptococcus pneumoniae | 1 | Unchanged | Improved | Unchanged |
| 1033825 | Pneumonia | NA | 1 | Human parainfluenza virus type 3; Coxsackievirus type A10; Haemophilus influenzae | 1 | Unchanged | Improved | Unchanged |
| 1033667 | Pneumonia | NA | 1 | Human metapneumovirus; Human parainfluenza virus type 1; Streptococcus pneumoniae | 1 | De-escalated | Improved | Discontinue medication |
| 1033662 | Pneumonia | NA | 1 | Coxsackievirus type A10; Rhinovirus type B | 1 | Unchanged | Improved | Unchanged |
| 1033651 | Pneumonia | NA | 1 | Coxsackievirus type A10; Rhinovirus type A | NA | Unchanged | Improved | Unchanged |
| 1033173 | Pneumonia | NA | 1 | Human parainfluenza virus type 3; Human coronavirus OC43; Bordetella pertussis | 1 | Escalated | Improved | Medication added |
| 1032688 | Pneumonia | NA | 1 | Rhinovirus type A | 1 | Unchanged | Improved | Unchanged |
| 1032583 | Pneumonia | NA | 1 | Human adenovirus type 2 | 1 | Escalated | Improved | Medication added |
| 1032422 | Pneumonia | NA | 1 | Human metapneumovirus | 1 | Unchanged | Improved | Unchanged |
| 1032250 | Pneumonia | NA | 1 | Human respiratory syncytial virus type B; Human bocavirus type 1 | 1 | Unchanged | Improved | Unchanged |
| 1032244 | Pneumonia | NA | 1 | Human parainfluenza virus type 3 | 1 | Escalated | Improved | Medication added |
| 1032050 | Pneumonia | NA | 0 | NA | 1 | Unchanged | Improved | Unchanged |
| 1031439 | Pneumonia | NA | 1 | Rhinovirus type A | 1 | Unchanged | Improved | Unchanged |
| 1031437 | Pneumonia | NA | 1 | Human metapneumovirus | 1 | Escalated | Improved | Medication added |
| 1031251 | Pneumonia | NA | 1 | Human coronavirus OC43 | 1 | Unchanged | Improved | Unchanged |
| 1031063 | Pneumonia | NA | 0 | NA | 1 | Unchanged | Improved | Unchanged |
| 1031055 | Pneumonia | NA | 1 | Rhinovirus type C; Bordetella pertussis | 1 | Escalated | Improved | Medication added |
| 1030733 | Pneumonia | NA | 1 | Human bocavirus type 1 | 0 | Unchanged | Improved | Unchanged |
| 1030651 | Pneumonia | NA | 1 | Human metapneumovirus; Streptococcus pneumoniae; Haemophilus influenzae | 1 | De-escalated | Improved | Reduce the amount |
| 1030311 | Pneumonia | NA | 1 | Mycoplasma pneumoniae | NA | Unchanged | Improved | Unchanged |
| 1029804 | Pneumonia | NA | 1 | Human coronavirus OC43; Rhinovirus type A; Streptococcus pneumoniae | 1 | Escalated | Improved | Medication added |
| 1028909 | Pneumonia | NA | 1 | Human respiratory syncytial virus type A; Rhinovirus type A | 1 | De-escalated | Improved | Discharge |
| 1028904 | Pneumonia | NA | 1 | Human respiratory syncytial virus type A | 0 | Unchanged | Aggravated | Unchanged |
| 1028511 | Pneumonia | NA | 0 | NA | 0 | De-escalated | Improved | Discharge |
| 1028214 | Pneumonia | NA | 0 | NA | 1 | De-escalated | Improved | Discontinue medication |
| 1028017 | Pneumonia | NA | 1 | Human respiratory syncytial virus type A | 0 | De-escalated | Improved | Discontinue medication |
| 1027751 | Pneumonia | NA | 1 | Rhinovirus type A | 1 | Unchanged | Improved | Unchanged |
| 1027678 | Pneumonia | NA | 1 | Human respiratory syncytial virus type A | 1 | De-escalated | Improved | Discontinue medication |
| 1027670 | Pneumonia | NA | 1 | Human respiratory syncytial virus type A | 1 | De-escalated | Improved | Discontinue medication |
| 1027392 | Pneumonia | NA | 1 | Human respiratory syncytial virus type A; Streptococcus pneumoniae | 1 | Escalated | Improved | Others |
| 1027134 | Pneumonia | NA | 1 | Human respiratory syncytial virus type A | 1 | De-escalated | Improved | Discharge |
| 1024755 | Pneumonia | NA | 1 | Mycoplasma pneumoniae | 1 | Escalated | Improved | Medication added |
| 1024185 | Pneumonia | NA | 1 | Human adenovirus type 3; Human coronavirus 229E | 1 | Unchanged | Improved | Unchanged |
| 1022877 | Pneumonia | NA | 1 | Human parainfluenza virus type 3 | NA | Unchanged | Improved | Unchanged |
| 1021870 | Pneumonia | NA | 1 | Mycoplasma pneumoniae; Rhinovirus | 1 | De-escalated | Improved | Discharge |
| 1021820 | Pneumonia | NA | 1 | Influenza A virus H3N2 | 1 | De-escalated | Improved | Discontinue medication |
| 1018349 | Pneumonia | NA | 0 | NA | 1 | Unchanged | Improved | Unchanged |
| 1015129 | Pneumonia | NA | 1 | Rhinovirus type B; Bordetella pertussis | 1 | De-escalated | Improved | Discontinue medication |
| 1014277 | Pneumonia | NA | 1 | Human bocavirus type 1 | 1 | Escalated | Improved | Medication added |
| 1013862 | Pneumonia | NA | 1 | Coxsackievirus type A10; Mycoplasma pneumoniae | 1 | De-escalated | Improved | Discharge |
| 1011611 | Pneumonia | NA | 1 | Human respiratory syncytial virus type A; Coxsackievirus type A6; Streptococcus pneumoniae | 1 | De-escalated | Improved | Discharge |
| 1011445 | Pneumonia | NA | 1 | Human respiratory syncytial virus type A | 1 | De-escalated | Improved | Discontinue medication |
| 1008417 | Pneumonia | NA | 1 | Human respiratory syncytial virus type A | 1 | Unchanged | Unchanged | Unchanged |
| 1006177 | Pneumonia | NA | 1 | Human parainfluenza virus type 4; Haemophilus influenzae | 1 | De-escalated | Improved | Discontinue medication |
| 1004262 | Pneumonia | NA | 1 | Human respiratory syncytial virus type A | 1 | De-escalated | Improved | Others |
| 1003043 | Pneumonia | NA | 1 | Human parainfluenza virus type 3; Rhinovirus type A; Haemophilus influenzae | 1 | De-escalated | Improved | Discontinue medication |
| 0997979 | Pneumonia | NA | 0 | NA | 1 | Unchanged | Improved | Unchanged |
| 1033517 | Neonatal pneumonia | NA | 1 | Human respiratory syncytial virus type A | 0 | Unchanged | Unchanged | Unchanged |
| 1025466 | Neonatal pneumonia | NA | 1 | Rhinovirus type A | 1 | Unchanged | Improved | Unchanged |
| 1031636 | Herpangina | NA | 1 | Human adenovirus type 2; Coxsackievirus type A10; Moraxella catarrhalis | NA | Unchanged | Improved | Unchanged |
| 1030644 | Herpangina | NA | 1 | Coxsackievirus type A5 | NA | Unchanged | Aggravated | Unchanged |
| 1001542 | Herpangina | NA | 1 | Coxsackievirus type A10 |  | De-escalated | Improved | Discharge |
| 1020419 | Febrile convulsions | NA | 1 | Human parainfluenza virus type 3 | NA | Unchanged | Improved | Unchanged |
| 1026370 | Bronchopneumonia, neutropenia | NA | 1 | Human respiratory syncytial virus type A | 1 | De-escalated | Improved | Discontinue medication |
| 1025090 | Bronchopneumonia thrush | NA | 1 | Human respiratory syncytial virus type A | 1 | De-escalated | Improved | Discharge |
| 1042088 | Bronchopneumonia | NA | 1 | Mycoplasma pneumoniae | 1 | Escalated | Improved | Medication added |
| 1041954 | Bronchopneumonia | NA | 1 | Influenza A virus H3N2 | 1 | Escalated | Improved | Others |
| 1041916 | Bronchopneumonia | NA | 1 | Influenza A virus H3N2; Bordetella pertussis | 1 | Escalated | Improved | Others |
| 1041533 | Bronchopneumonia | NA | 1 | Influenza A virus; Haemophilus influenzae | 1 | Escalated | Improved | Medication added |
| 1041505 | Bronchopneumonia | NA | 1 | Influenza A virus H3N2 | 1 | Escalated | Improved | Others |
| 1041438 | Bronchopneumonia | NA | 1 | Influenza A virus H3N2; Streptococcus pneumoniae | 1 | Escalated | Improved | Medication added |
| 1041326 | Bronchopneumonia | NA | 1 | Influenza A virus H3N2 | 1 | Escalated | Improved | Medication added |
| 1041325 | Bronchopneumonia | NA | 1 | Human metapneumovirus; Influenza A virus H3N2 | 1 | Escalated | Improved | Others |
| 1041003 | Bronchopneumonia | NA | 1 | Human bocavirus type 1 | 1 | Unchanged | Improved | Unchanged |
| 1040916 | Bronchopneumonia | NA | 1 | Mycoplasma pneumoniae; Rhinovirus type C | 1 | Escalated | Improved | Medication added |
| 1040773 | Bronchopneumonia | NA | 1 | Influenza A virus H3N2; Bordetella pertussis | 1 | De-escalated | Improved | Reduce the amount |
| 1040509 | Bronchopneumonia | NA | 1 | Influenza A virus H3N2; Mycoplasma pneumoniae; Epstein-Barr virus | 1 | Unchanged | Improved | Unchanged |
| 1040420 | Bronchopneumonia | NA | 1 | Streptococcus pneumoniae | 1 | Unchanged | Improved | Unchanged |
| 1040366 | Bronchopneumonia | NA | 1 | Rhinovirus type C; Bordetella pertussis | 1 | Unchanged | Improved | Unchanged |
| 1040327 | Bronchopneumonia | NA | 1 | Influenza A virus H3N2 | 1 | Escalated | Improved | Others |
| 1039924 | Bronchopneumonia | NA | 1 | Rhinovirus type C | 1 | Unchanged | Improved | Unchanged |
| 1039519 | Bronchopneumonia | NA | 1 | Human parainfluenza virus type 3 | 1 | Unchanged | Improved | Unchanged |
| 1039341 | Bronchopneumonia | NA | 1 | Cytomegalovirus | 1 | Unchanged | Improved | Unchanged |
| 1039295 | Bronchopneumonia | NA | 1 | Human parainfluenza virus type 3 | 1 | Unchanged | Improved | Unchanged |
| 1039225 | Bronchopneumonia | NA | 0 | NA | 1 | Unchanged | Improved | Unchanged |
| 1039216 | Bronchopneumonia | NA | 1 | Human coronavirus OC43; Rhinovirus type C | 1 | Unchanged | Improved | Unchanged |
| 1039024 | Bronchopneumonia | NA | 1 | Rhinovirus type C; Streptococcus pneumoniae | 1 | Unchanged | Improved | Unchanged |
| 1038947 | Bronchopneumonia | NA | 1 | Human parainfluenza virus type 1; Haemophilus influenzae | 1 | De-escalated | Improved | Reduce the amount |
| 1038824 | Bronchopneumonia | NA | 1 | Human parainfluenza virus type 3; Human bocavirus type 1; Influenza A virus | 1 | Escalated | Improved | Medication added |
| 1038805 | Bronchopneumonia | NA | 1 | Human metapneumovirus | 1 | Escalated | Improved | Medication added |
| 1038756 | Bronchopneumonia | NA | 0 | NA | 1 | Unchanged | Improved | Unchanged |
| 1038598 | Bronchopneumonia | NA | 1 | Mycoplasma pneumoniae | 1 | Unchanged | Improved | Unchanged |
| 1038298 | Bronchopneumonia | NA | 0 | NA | 1 | Unchanged | Improved | Unchanged |
| 1038292 | Bronchopneumonia | NA | 1 | Rhinovirus type A; Bordetella pertussis; Streptococcus pneumoniae | 1 | Escalated | Improved | Others |
| 1038274 | Bronchopneumonia | NA | 1 | Human parainfluenza virus type 1 | 1 | Unchanged | Improved | Unchanged |
| 1038152 | Bronchopneumonia | NA | 1 | Influenza A virus H3N2; Herpes simplex virus type 1; Haemophilus influenzae | 1 | Escalated | Improved | Medication added |
| 1038147 | Bronchopneumonia | NA | 1 | Human parainfluenza virus type 3; Coxsackievirus type A6; Stenotrophomonas maltophilia; Cytomegalovirus | 1 | Unchanged | Improved | Unchanged |
| 1038071 | Bronchopneumonia | NA | 1 | Influenza A virus H3N2 | 1 | Unchanged | Improved | Unchanged |
| 1037937 | Bronchopneumonia | NA | 0 | NA | 1 | Unchanged | Improved | Unchanged |
| 1037818 | Bronchopneumonia | NA | 1 | Rhinovirus type A; Streptococcus pneumoniae | 1 | Unchanged | Improved | Unchanged |
| 1037578 | Bronchopneumonia | NA | 1 | Human respiratory syncytial virus type A; Mycoplasma pneumoniae | 1 | Unchanged | Improved | Unchanged |
| 1037508 | Bronchopneumonia | NA | 1 | Human coronavirus OC43; Rhinovirus type A; Haemophilus influenzae | 1 | Unchanged | Improved | Unchanged |
| 1037254 | Bronchopneumonia | NA | 1 | Epstein-Barr virus | 1 | Unchanged | Improved | Unchanged |
| 1037185 | Bronchopneumonia | NA | 1 | Human parainfluenza virus type 4; Herpes simplex virus type 1 | 1 | Unchanged | Improved | Unchanged |
| 1037175 | Bronchopneumonia | NA | 1 | Mycoplasma pneumoniae | 1 | Unchanged | Improved | Unchanged |
| 1037174 | Bronchopneumonia | NA | 1 | Bordetella pertussis; Haemophilus influenzae | 1 | Escalated | Improved | Others |
| 1036917 | Bronchopneumonia | NA | 1 | Bordetella pertussis | 1 | Unchanged | Improved | Unchanged |
| 1036854 | Bronchopneumonia | NA | 0 | NA | NA | Unchanged | Improved | Unchanged |
| 1036792 | Bronchopneumonia | NA | 1 | Human adenovirus type 3 | 1 | Escalated | Improved | Medication added |
| 1036753 | Bronchopneumonia | NA | 1 | Human metapneumovirus | 1 | Unchanged | Improved | Unchanged |
| 1036632 | Bronchopneumonia | NA | 1 | Human parainfluenza virus type 1; Streptococcus pneumoniae | 1 | Unchanged | Improved | Unchanged |
| 1036469 | Bronchopneumonia | NA | 1 | Human metapneumovirus; Haemophilus influenzae; Streptococcus pneumoniae | 1 | Unchanged | Improved | Unchanged |
| 1035804 | Bronchopneumonia | NA | 0 | NA | 1 | Unchanged | Improved | Unchanged |
| 1035769 | Bronchopneumonia | NA | 1 | Human metapneumovirus; Coxsackievirus type A6 | 1 | Escalated | Improved | Medication added |
| 1035574 | Bronchopneumonia | NA | 1 | Human metapneumovirus | 1 | Unchanged | Improved | Unchanged |
| 1035567 | Bronchopneumonia | NA | 1 | Human parainfluenza virus type 3; Rhinovirus type A; Haemophilus influenzae | 1 | Unchanged | Improved | Unchanged |
| 1035548 | Bronchopneumonia | NA | 1 | Human metapneumovirus; Haemophilus influenzae | 1 | Escalated | Improved | Medication added |
| 1035497 | Bronchopneumonia | NA | 1 | Influenza A virus H3N2; Streptococcus pneumoniae | 1 | Unchanged | Improved | Unchanged |
| 1035495 | Bronchopneumonia | NA | 1 | Human metapneumovirus; Streptococcus pneumoniae | 1 | Unchanged | Improved | Unchanged |
| 1035479 | Bronchopneumonia | NA | 1 | Rhinovirus type A | 1 | Unchanged | Improved | Unchanged |
| 1035468 | Bronchopneumonia | NA | 1 | Human metapneumovirus | 1 | De-escalated | Improved | Discontinue medication |
| 1035405 | Bronchopneumonia | NA | 1 | Human metapneumovirus | 1 | Unchanged | Improved | Unchanged |
| 1035387 | Bronchopneumonia | NA | 1 | Human parainfluenza virus type 3 | 1 | Unchanged | Improved | Unchanged |
| 1035371 | Bronchopneumonia | NA | 1 | Human respiratory syncytial virus type B; Human parainfluenza virus type 3; Human bocavirus type 1; Herpes simplex virus type 1 | 1 | Escalated | Improved | Medication added |
| 1035172 | Bronchopneumonia | NA | 1 | Human metapneumovirus; Human respiratory syncytial virus type A | 1 | De-escalated | Improved | Discontinue medication |
| 1034961 | Bronchopneumonia | NA | 1 | Human metapneumovirus | 1 | Unchanged | Improved | Unchanged |
| 1034872 | Bronchopneumonia | NA | 1 | Mycoplasma pneumoniae | 1 | Unchanged | Improved | Unchanged |
| 1034785 | Bronchopneumonia | NA | 0 | NA | 1 | Unchanged | Improved | Unchanged |
| 1034777 | Bronchopneumonia | NA | 1 | Human respiratory syncytial virus type A | 1 | Unchanged | Improved | Unchanged |
| 1034700 | Bronchopneumonia | NA | 1 | Human respiratory syncytial virus type A; Coxsackievirus type A10 | 1 | Unchanged | Improved | Unchanged |
| 1034699 | Bronchopneumonia | NA | 1 | Human metapneumovirus | 1 | Unchanged | Improved | Unchanged |
| 1034691 | Bronchopneumonia | NA | 1 | Human metapneumovirus | 1 | Unchanged | Improved | Unchanged |
| 1034680 | Bronchopneumonia | NA | 1 | Haemophilus influenzae | 1 | Escalated | Improved | Medications changed |
| 1034477 | Bronchopneumonia | NA | 0 | NA | 1 | Unchanged | Improved | Unchanged |
| 1034475 | Bronchopneumonia | NA | 1 | Epstein-Barr virus | 1 | Unchanged | Improved | Unchanged |
| 1034467 | Bronchopneumonia | NA | 1 | Human metapneumovirus | 1 | Unchanged | Improved | Unchanged |
| 1034298 | Bronchopneumonia | NA | 1 | Streptococcus pyogenes | 1 | Unchanged | Improved | Unchanged |
| 1034269 | Bronchopneumonia | NA | 1 | Human parainfluenza virus type 4; Rhinovirus type A; Streptococcus pneumoniae | 1 | Unchanged | Improved | Unchanged |
| 1034206 | Bronchopneumonia | NA | 1 | Human metapneumovirus; Streptococcus pneumoniae | 1 | Unchanged | Improved | Unchanged |
| 1034198 | Bronchopneumonia | NA | 0 | NA | 1 | Unchanged | Improved | Unchanged |
| 1034191 | Bronchopneumonia | NA | 1 | Epstein-Barr virus; Streptococcus pneumoniae; Haemophilus influenzae | 1 | Unchanged | Improved | Unchanged |
| 1034123 | Bronchopneumonia | NA | 1 | Human metapneumovirus; Streptococcus pneumoniae | 1 | Unchanged | Improved | Unchanged |
| 1034015 | Bronchopneumonia | NA | 1 | Bordetella pertussis | 1 | Unchanged | Improved | Unchanged |
| 1033835 | Bronchopneumonia | NA | 1 | Human metapneumovirus | 1 | Unchanged | Improved | Unchanged |
| 1033833 | Bronchopneumonia | NA | 1 | Human parainfluenza virus type 3; Rhinovirus type A | 1 | Unchanged | Improved | Unchanged |
| 1033824 | Bronchopneumonia | NA | 1 | Human parainfluenza virus type 4; Coxsackievirus type A6 | 1 | Unchanged | Improved | Unchanged |
| 1033820 | Bronchopneumonia | NA | 1 | Human bocavirus type 1 | 1 | Unchanged | Improved | Unchanged |
| 1033760 | Bronchopneumonia | NA | 1 | Human metapneumovirus | 1 | Unchanged | Improved | Unchanged |
| 1033744 | Bronchopneumonia | NA | 1 | Human metapneumovirus | 1 | Unchanged | Improved | Unchanged |
| 1033738 | Bronchopneumonia | NA | 1 | Human metapneumovirus; Human bocavirus type 1; Coxsackievirus type A10; Stenotrophomonas maltophilia | 1 | Unchanged | Improved | Unchanged |
| 1033668 | Bronchopneumonia | NA | 1 | Human metapneumovirus | 1 | De-escalated | Improved | Discontinue medication |
| 1033664 | Bronchopneumonia | NA | 1 | Human parainfluenza virus type 1 | 1 | Unchanged | Improved | Unchanged |
| 1033645 | Bronchopneumonia | NA | 1 | Human parainfluenza virus type 1; Rhinovirus type A; Streptococcus pneumoniae | 1 | De-escalated | Improved | Discontinue medication |
| 1033589 | Bronchopneumonia | NA | 0 | NA | 1 | Unchanged | Improved | Unchanged |
| 1033579 | Bronchopneumonia | NA | 1 | Human parainfluenza virus type 3; Cytomegalovirus | 1 | Escalated | Improved | Medication added |
| 1033513 | Bronchopneumonia | NA | 1 | Human metapneumovirus; Human parainfluenza virus type 3; Haemophilus influenzae | 0 | Unchanged | Improved | Unchanged |
| 1033504 | Bronchopneumonia | NA | 0 | NA | 0 | Unchanged | Improved | Unchanged |
| 1033434 | Bronchopneumonia | NA | 1 | Human parainfluenza virus type 3; Human bocavirus type 1 | 1 | De-escalated | Improved | Discontinue medication |
| 1033348 | Bronchopneumonia | NA | 0 | NA | 1 | Escalated | Improved | Others |
| 1033329 | Bronchopneumonia | NA | 1 | Human parainfluenza virus type 3 | 1 | De-escalated | Improved | Others |
| 1033233 | Bronchopneumonia | NA | 1 | Human parainfluenza virus type 3; Haemophilus influenzae | 1 | Escalated | Improved | Medication added |
| 1033217 | Bronchopneumonia | NA | 1 | Human metapneumovirus; Human coronavirus HKU1; Haemophilus influenzae | 0 | De-escalated | Improved | Discontinue medication |
| 1033209 | Bronchopneumonia | NA | 1 | Human metapneumovirus | 1 | De-escalated | Improved | Others |
| 1033139 | Bronchopneumonia | NA | 1 | Human parainfluenza virus type 3; Rhinovirus type A; Streptococcus pneumoniae | 1 | De-escalated | Improved | Discontinue medication |
| 1033076 | Bronchopneumonia | NA | 1 | Human respiratory syncytial virus type A; Haemophilus influenzae | 1 | Unchanged | Improved | Unchanged |
| 1033065 | Bronchopneumonia | NA | 1 | Human adenovirus type 1; Human metapneumovirus | 0 | Escalated | Improved | Medication added |
| 1032993 | Bronchopneumonia | NA | 1 | Moraxella catarrhalis | 0 | De-escalated | Improved | Discontinue medication |
| 1032991 | Bronchopneumonia | NA | 1 | Human metapneumovirus; Bordetella pertussis | 1 | Unchanged | Improved | Unchanged |
| 1032956 | Bronchopneumonia | NA | 1 | Human parainfluenza virus type 1; Mycoplasma pneumoniae; Rhinovirus type A | 1 | De-escalated | Improved | Discontinue medication |
| 1032880 | Bronchopneumonia | NA | 1 | Human respiratory syncytial virus type A | 1 | Unchanged | Improved | Unchanged |
| 1032813 | Bronchopneumonia | NA | 1 | Human metapneumovirus; Haemophilus influenzae; Streptococcus pneumoniae | 0 | Escalated | Improved | Medication added |
| 1032801 | Bronchopneumonia | NA | 1 | Human respiratory syncytial virus type A; Human parainfluenza virus type 1 | 1 | Unchanged | Improved | Unchanged |
| 1032695 | Bronchopneumonia | NA | 1 | Rhinovirus type A | 0 | Unchanged | Improved | Unchanged |
| 1032691 | Bronchopneumonia | NA | 1 | Human metapneumovirus; Streptococcus pneumoniae | 1 | Unchanged | Improved | Unchanged |
| 1032607 | Bronchopneumonia | NA | 1 | Human metapneumovirus | 0 | Unchanged | Improved | Unchanged |
| 1032593 | Bronchopneumonia | NA | 1 | Human respiratory syncytial virus type A | 1 | Unchanged | Improved | Unchanged |
| 1032586 | Bronchopneumonia | NA | 1 | Rhinovirus type A | 1 | Unchanged | Improved | Unchanged |
| 1032418 | Bronchopneumonia | NA | 1 | Rhinovirus type A; Bordetella pertussis | 1 | Escalated | Improved | Medication added |
| 1032145 | Bronchopneumonia | NA | 1 | Human parainfluenza virus type 4; Human bocavirus type 1; Streptococcus pneumoniae | 1 | Unchanged | Improved | Unchanged |
| 1032130 | Bronchopneumonia | NA | 1 | Human parainfluenza virus type 1 | 1 | De-escalated | Improved | Discharge |
| 1032047 | Bronchopneumonia | NA | 1 | Rhinovirus type A; Moraxella catarrhalis; Streptococcus pneumoniae; Haemophilus influenzae | 1 | De-escalated | Improved | Discontinue medication |
| 1031949 | Bronchopneumonia | NA | 1 | Human metapneumovirus; Rhinovirus type B; Haemophilus influenzae | 1 | Unchanged | Improved | Unchanged |
| 1031896 | Bronchopneumonia | NA | 1 | Herpes simplex virus type 1 | 1 | Escalated | Improved | Medication added |
| 1031894 | Bronchopneumonia | NA | 1 | Human parainfluenza virus type 3; Rhinovirus type A; Streptococcus pneumoniae; Cytomegalovirus | 1 | Unchanged | Improved | Unchanged |
| 1031893 | Bronchopneumonia | NA | 1 | Human parainfluenza virus type 3; Human bocavirus type 1; Haemophilus influenzae | 0 | Escalated | Improved | Medication added |
| 1031814 | Bronchopneumonia | NA | 0 | NA | 1 | De-escalated | Improved | Discontinue medication |
| 1031740 | Bronchopneumonia | NA | 1 | Bordetella pertussis | 1 | Unchanged | Improved | Unchanged |
| 1031635 | Bronchopneumonia | NA | 1 | Human metapneumovirus; Haemophilus influenzae | 1 | Escalated | Improved | Medication added |
| 1031215 | Bronchopneumonia | NA | 1 | Rhinovirus type A; Haemophilus influenzae | 0 | Escalated | Improved | Medication added |
| 1031058 | Bronchopneumonia | NA | 1 | Human respiratory syncytial virus type A | 0 | Unchanged | Improved | Unchanged |
| 1031057 | Bronchopneumonia | NA | 1 | Human adenovirus group C; Streptococcus pneumoniae | 1 | Unchanged | Improved | Unchanged |
| 1030939 | Bronchopneumonia | NA | 1 | Human metapneumovirus; Moraxella catarrhalis | 1 | Unchanged | Improved | Unchanged |
| 1030913 | Bronchopneumonia | NA | 1 | Haemophilus influenzae | NA | Escalated | Improved | Medication added |
| 1030834 | Bronchopneumonia | NA | 1 | Epstein-Barr virus | 1 | De-escalated | Improved | Discharge |
| 1030561 | Bronchopneumonia | NA | 1 | Human bocavirus type 1 | 1 | Unchanged | Improved | Unchanged |
| 1030430 | Bronchopneumonia | NA | 1 | Human metapneumovirus; Bordetella pertussis; Haemophilus influenzae; Staphylococcus aureus | 1 | Unchanged | Improved | Unchanged |
| 1030372 | Bronchopneumonia | NA | 1 | Rhinovirus type A; Bordetella pertussis; Streptococcus pneumoniae | 1 | Escalated | Improved | Medication added |
| 1030321 | Bronchopneumonia | NA | 1 | Human metapneumovirus | 1 | Unchanged | Improved | Unchanged |
| 1030200 | Bronchopneumonia | NA | 0 | NA | 1 | Unchanged | Improved | Unchanged |
| 1030142 | Bronchopneumonia | NA | 1 | Human respiratory syncytial virus type B | 1 | De-escalated | Improved | Discontinue medication |
| 1029905 | Bronchopneumonia | NA | 1 | Human respiratory syncytial virus type B | 1 | Unchanged | Improved | Unchanged |
| 1029898 | Bronchopneumonia | NA | 1 | Human bocavirus type 1; Epstein-Barr virus | 1 | Unchanged | Improved | Unchanged |
| 1029893 | Bronchopneumonia | NA | 1 | Rhinovirus type A; Haemophilus influenzae; Streptococcus pneumoniae | 1 | Unchanged | Improved | Unchanged |
| 1029877 | Bronchopneumonia | NA | 1 | Human metapneumovirus; Haemophilus influenzae | 0 | Unchanged | Improved | Unchanged |
| 1029709 | Bronchopneumonia | NA | 1 | Human respiratory syncytial virus type A | 1 | Unchanged | Improved | Unchanged |
| 1029705 | Bronchopneumonia | NA | 1 | Human parainfluenza virus type 1; Moraxella catarrhalis; Streptococcus pneumoniae | 0 | Unchanged | Improved | Unchanged |
| 1029692 | Bronchopneumonia | NA | 0 | NA | 0 | Unchanged | Improved | Unchanged |
| 1029628 | Bronchopneumonia | NA | 1 | Human respiratory syncytial virus type A | 0 | Escalated | Improved | Medication added |
| 1029331 | Bronchopneumonia | NA | 1 | Human coronavirus OC43; Rhinovirus type A | 1 | Unchanged | Improved | Unchanged |
| 1029330 | Bronchopneumonia | NA | 1 | Rhinovirus type A | 0 | De-escalated | Improved | Discharge |
| 1029256 | Bronchopneumonia | NA | 1 | Human respiratory syncytial virus type A | 1 | Escalated | Improved | Medication added |
| 1029244 | Bronchopneumonia | NA | 0 | NA | 1 | Unchanged | Improved | Unchanged |
| 1029214 | Bronchopneumonia | NA | 1 | Human respiratory syncytial virus type B | 1 | De-escalated | Improved | Reduce the amount |
| 1029111 | Bronchopneumonia | NA | 1 | Human respiratory syncytial virus type A | 1 | Unchanged | Improved | Unchanged |
| 1029095 | Bronchopneumonia | NA | 1 | Human respiratory syncytial virus type B; Haemophilus influenzae; Streptococcus pneumoniae | 1 | Unchanged | Improved | Unchanged |
| 1029073 | Bronchopneumonia | NA | 1 | Human respiratory syncytial virus type A | 0 | De-escalated | Improved | Discharge |
| 1029063 | Bronchopneumonia | NA | 1 | Human respiratory syncytial virus type A | 1 | De-escalated | Improved | Discharge |
| 1028889 | Bronchopneumonia | NA | 1 | Human respiratory syncytial virus type A | 1 | Unchanged | Improved | Unchanged |
| 1028807 | Bronchopneumonia | NA | 0 | NA | 1 | Unchanged | Improved | Unchanged |
| 1028732 | Bronchopneumonia | NA | 1 | Human coronavirus OC43; Moraxella catarrhalis | 0 | Unchanged | Improved | Unchanged |
| 1028714 | Bronchopneumonia | NA | 1 | Human adenovirus group C; Human respiratory syncytial virus type A; Human coronavirus OC43; Streptococcus pneumoniae | 1 | Unchanged | Improved | Unchanged |
| 1028706 | Bronchopneumonia | NA | 0 | NA | 0 | Unchanged | Improved | Unchanged |
| 1028598 | Bronchopneumonia | NA | 1 | Human metapneumovirus | 1 | Unchanged | Improved | Unchanged |
| 1028456 | Bronchopneumonia | NA | 1 | Streptococcus pyogenes; Epstein-Barr virus | 1 | Escalated | Improved | Medications changed |
| 1028312 | Bronchopneumonia | NA | 1 | Human respiratory syncytial virus type A; Human coronavirus OC43 | 1 | Unchanged | Improved | Unchanged |
| 1028261 | Bronchopneumonia | NA | 1 | Human respiratory syncytial virus type A | 1 | Escalated | Improved | Medication added |
| 1028123 | Bronchopneumonia | NA | 1 | Human coronavirus OC43 | 1 | Unchanged | Improved | Unchanged |
| 1028116 | Bronchopneumonia | NA | 1 | Human bocavirus type 1 | 0 | De-escalated | Improved | Discontinue medication |
| 1027925 | Bronchopneumonia | NA | 1 | Human coronavirus OC43; Haemophilus influenzae | 1 | Unchanged | Improved | Unchanged |
| 1027910 | Bronchopneumonia | NA | 0 | NA | 1 | Escalated | Improved | Medications changed |
| 1027742 | Bronchopneumonia | NA | 1 | Human respiratory syncytial virus type A; Streptococcus pneumoniae | 1 | De-escalated | Improved | Discontinue medication |
| 1027689 | Bronchopneumonia | NA | 1 | Human coronavirus OC43; Streptococcus pneumoniae | 1 | De-escalated | Improved | Discontinue medication |
| 1027508 | Bronchopneumonia | NA | 1 | Human parainfluenza virus type 3 | 1 | Unchanged | Improved | Unchanged |
| 1027471 | Bronchopneumonia | NA | 1 | Human respiratory syncytial virus type A; Cytomegalovirus | 1 | Unchanged | Improved | Unchanged |
| 1027468 | Bronchopneumonia | NA | 1 | Human respiratory syncytial virus type A | 1 | Escalated | Improved | Medication added |
| 1027431 | Bronchopneumonia | NA | 1 | Human respiratory syncytial virus type A | 1 | Unchanged | Improved | Unchanged |
| 1027184 | Bronchopneumonia | NA | 1 | Human respiratory syncytial virus type A | 1 | Escalated | Improved | Medication added |
| 1027039 | Bronchopneumonia | NA | 1 | Human coronavirus OC43; Moraxella catarrhalis | 1 | Escalated | Improved | Medication added |
| 1026997 | Bronchopneumonia | NA | 1 | Rhinovirus type A | 1 | Unchanged | Improved | Unchanged |
| 1026727 | Bronchopneumonia | NA | 1 | Influenza A virus | 0 | Escalated | Improved | Medication added |
| 1026630 | Bronchopneumonia | NA | 1 | Human respiratory syncytial virus type A | 1 | Unchanged | Improved | Unchanged |
| 1026596 | Bronchopneumonia | NA | 1 | Influenza A virus; Moraxella catarrhalis; Cytomegalovirus | 1 | Escalated | Improved | Others |
| 1026570 | Bronchopneumonia | NA | 1 | Human respiratory syncytial virus type A | 0 | Unchanged | Improved | Unchanged |
| 1026562 | Bronchopneumonia | NA | 1 | Human respiratory syncytial virus type A | 1 | Unchanged | Improved | Unchanged |
| 1026045 | Bronchopneumonia | NA | 0 | NA | 1 | De-escalated | Improved | Discontinue medication |
| 1025973 | Bronchopneumonia | NA | 0 | NA | 1 | Unchanged | Improved | Unchanged |
| 1025778 | Bronchopneumonia | NA | 0 | NA | 1 | Unchanged | Improved | Unchanged |
| 1025713 | Bronchopneumonia | NA | 0 | NA | 1 | De-escalated | Improved | Discontinue medication |
| 1025639 | Bronchopneumonia | NA | 1 | Influenza A virus | 1 | De-escalated | Improved | Discharge |
| 1025209 | Bronchopneumonia | NA | 1 | Human respiratory syncytial virus type A; Streptococcus pneumoniae | 1 | Unchanged | Improved | Unchanged |
| 1025201 | Bronchopneumonia | NA | 1 | Human respiratory syncytial virus type A; Streptococcus pneumoniae | 1 | Unchanged | Improved | Unchanged |
| 1024908 | Bronchopneumonia | NA | 1 | Human adenovirus type 1; Bordetella pertussis; Streptococcus pneumoniae | NA | Escalated | Improved | Medication added |
| 1024632 | Bronchopneumonia | NA | 1 | Influenza A virus H3N2 | 1 | De-escalated | Improved | Discharge |
| 1024515 | Bronchopneumonia | NA | 1 | Human metapneumovirus; Rhinovirus type A; Streptococcus pneumoniae | 1 | De-escalated | Improved | Discontinue medication |
| 1024336 | Bronchopneumonia | NA | 1 | Human respiratory syncytial virus type A | 1 | Escalated | Improved | Medication added |
| 1023512 | Bronchopneumonia | NA | 1 | Rhinovirus type B; Haemophilus influenzae | 1 | Escalated | Improved | Medication added |
| 1022505 | Bronchopneumonia | NA | 1 | Human parainfluenza virus type 3; Cytomegalovirus | 0 | Escalated | Improved | Others |
| 994161 | Bronchitis | NA | 1 | Bordetella pertussis; Streptococcus pneumoniae; Haemophilus influenzae | 1 | Escalated | Improved | Medication added |
| 961500 | Bronchitis | NA | 1 | Influenza A virus | 1 | De-escalated | Improved | Discontinue medication |
| 960973 | Bronchitis | NA | 1 | Streptococcus pneumoniae | 1 | Unchanged | Improved | Unchanged |
| 1045711 | Bronchitis | NA | 1 | Human adenovirus type 3; Streptococcus pneumoniae | 1 | De-escalated | Improved | Discontinue medication |
| 1041812 | Bronchitis | NA | 1 | Human adenovirus type 1; Influenza A virus H3N2; Rhinovirus type A; Haemophilus influenzae | 1 | Escalated | Improved | Medication added |
| 1037736 | Bronchitis | NA | 1 | Coxsackievirus type A6; Epstein-Barr virus | 1 | Escalated | Improved | Others |
| 1034499 | Bronchitis | NA | 1 | Rhinovirus type A | 1 | Escalated | Improved | Medication added |
| 1032016 | Bronchitis | NA | 1 | Human metapneumovirus; Streptococcus pneumoniae | 1 | Escalated | Improved | Medication added |
| 1028896 | Bronchitis | NA | 1 | Human respiratory syncytial virus type B; Staphylococcus aureus; Streptococcus pneumoniae | 0 | Escalated | Improved | Medication added |
| 922029 | Bronchiolitis | NA | 0 | NA | 1 | De-escalated | Improved | Discontinue medication |
| 1018870 | Bronchiolitis | NA | 1 | Human respiratory syncytial virus type A; Klebsiella pneumoniae | 1 | Escalated | Improved | Medication added |
| 1004571 | Bronchiolitis | NA | 1 | Rhinovirus type C; Haemophilus influenzae | 1 | De-escalated | Improved | Discontinue medication |
| 1033069 | Bacterial pneumonia | NA | 1 | Human metapneumovirus; Human parainfluenza virus type 1 | 1 | De-escalated | Improved | Discontinue medication |
| 995399 | Asthematoid bronchopneumonia | NA | 1 | Human parainfluenza virus type 1; Coxsackievirus type A6; Rhinovirus type A | 1 | De-escalated | Improved | Others |
| 1044711 | Asthematoid bronchopneumonia | NA | 1 | Human metapneumovirus | 1 | De-escalated | Improved | Discontinue medication |
| 1044525 | Asthematoid bronchopneumonia | NA | 1 | Human respiratory syncytial virus type A; Influenza A virus; Cytomegalovirus | 1 | Escalated | Improved | Medication added |
| 1035192 | Asthematoid bronchopneumonia | NA | 1 | Human parainfluenza virus type 3 | 1 | Unchanged | Improved | Unchanged |
| 1035062 | Asthematoid bronchopneumonia | NA | 0 | NA | 1 | Unchanged | Improved | Unchanged |
| 1034559 | Asthematoid bronchopneumonia | NA | 1 | Human adenovirus type 1; Human parainfluenza virus type 3; Epstein-Barr virus | 1 | Unchanged | Improved | Unchanged |
| 1033946 | Asthematoid bronchopneumonia | NA | 1 | Human bocavirus type 1 | 1 | Unchanged | Improved | Unchanged |
| 1033759 | Asthematoid bronchopneumonia | NA | 1 | Human parainfluenza virus type 3; Haemophilus influenzae | 1 | Unchanged | Improved | Unchanged |
| 1032716 | Asthematoid bronchopneumonia | NA | 1 | Rhinovirus type A; Streptococcus pneumoniae; Moraxella catarrhalis | 1 | De-escalated | Improved | Discontinue medication |
| 1032128 | Asthematoid bronchopneumonia | NA | 1 | Human respiratory syncytial virus type A; Coxsackievirus type A10; Cytomegalovirus | 0 | Unchanged | Improved | Unchanged |
| 1031339 | Asthematoid bronchopneumonia | NA | 1 | Human respiratory syncytial virus type A; Cytomegalovirus | 0 | Unchanged | Aggravated | Unchanged |
| 1031054 | Asthematoid bronchopneumonia | NA | 1 | Human metapneumovirus | 1 | Unchanged | Improved | Unchanged |
| 1030484 | Asthematoid bronchopneumonia | NA | 1 | Human bocavirus type 1 | 1 | Escalated | Improved | Medication added |
| 1030447 | Asthematoid bronchopneumonia | NA | 1 | Human bocavirus type 1 | 0 | De-escalated | Improved | Discontinue medication |
| 1029735 | Asthematoid bronchopneumonia | NA | 1 | Human respiratory syncytial virus type A | 1 | De-escalated | Improved | Reduce the amount |
| 1029076 | Asthematoid bronchopneumonia | NA | 1 | Human respiratory syncytial virus type A | 1 | De-escalated | Improved | Discontinue medication |
| 1027851 | Asthematoid bronchopneumonia | NA | 1 | Human respiratory syncytial virus type B | 1 | Escalated | Improved | Medication added |
| 1027661 | Asthematoid bronchopneumonia | NA | 0 | NA | 1 | De-escalated | Improved | Discontinue medication |
| 1027388 | Asthematoid bronchopneumonia | NA | 1 | Human respiratory syncytial virus type A; Haemophilus influenzae | 0 | De-escalated | Improved | Discontinue medication |
| 1027286 | Asthematoid bronchopneumonia | NA | 1 | Human respiratory syncytial virus type A | 0 | Escalated | Improved | Medication added |
| 1022594 | Asthematoid bronchopneumonia | NA | 1 | Rhinovirus type A | 1 | De-escalated | Improved | Discontinue medication |
| 1021413 | Asthematoid bronchopneumonia | NA | 1 | Human respiratory syncytial virus type A | 0 | Unchanged | Improved | Unchanged |
| 0997104 | Asthematoid bronchopneumonia | NA | 0 | NA | 1 | De-escalated | Improved | Discontinue medication |
| 1020418 | Acute upper respiratory tract infection, rhinitis | NA | 1 | Human coronavirus OC43 | NA | Unchanged | Improved | Unchanged |
| 980753 | Acute upper respiratory tract infection | NA | 0 | NA | NA | Unchanged | Unchanged | Unchanged |
| 957818 | Acute upper respiratory tract infection | NA | 0 | NA | NA | De-escalated | Improved | Discontinue medication |
| 953019 | Acute upper respiratory tract infection | NA | 1 | Coxsackievirus type A10 | NA | De-escalated | Improved | Discontinue medication |
| 876702 | Acute upper respiratory tract infection | NA | 0 | NA | NA | De-escalated | Improved | Discharge |
| 872669 | Acute upper respiratory tract infection | NA | 1 | Human adenovirus type 3 | 0 | De-escalated | Improved | Discontinue medication |
| 1045585 | Acute upper respiratory tract infection | NA | 1 | Human adenovirus type 5; Staphylococcus aureus; Klebsiella pneumoniae | NA | Escalated | Improved | Medication added |
| 1044294 | Acute upper respiratory tract infection | NA | 1 | Influenza A virus H3N2 | NA | Escalated | Improved | Nebulization of interferon |
| 1042142 | Acute upper respiratory tract infection | NA | 1 | Streptococcus pneumoniae | NA | Unchanged | Improved | Unchanged |
| 1040688 | Acute upper respiratory tract infection | NA | 1 | Human adenovirus type 3 | NA | Unchanged | Improved | Unchanged |
| 1038346 | Acute upper respiratory tract infection | NA | 0 | NA | NA | Unchanged | Improved | Unchanged |
| 1036995 | Acute upper respiratory tract infection | NA | 1 | Rhinovirus type A | NA | Unchanged | Improved | Unchanged |
| 1036889 | Acute upper respiratory tract infection | NA | 1 | Human adenovirus type 3; Herpes simplex virus type 1 | NA | Unchanged | Improved | Unchanged |
| 1035904 | Acute upper respiratory tract infection | NA | 1 | Coxsackievirus type A10 | NA | Unchanged | Improved | Unchanged |
| 1035460 | Acute upper respiratory tract infection | NA | 1 | SARS-CoV-2 omicron XBB | NA | Unchanged | Improved | Unchanged |
| 1035375 | Acute upper respiratory tract infection | NA | 1 | Herpes simplex virus type 1; Rhinovirus type A; Moraxella catarrhalis | NA | Escalated | Improved | Medication added |
| 1034139 | Acute upper respiratory tract infection | NA | 1 | Human adenovirus type 1; Coxsackievirus type A10; Haemophilus influenzae; Moraxella catarrhalis | NA | Unchanged | Improved | Unchanged |
| 1033426 | Acute upper respiratory tract infection | NA | 1 | Human adenovirus group C | NA | De-escalated | Improved | Discharge |
| 1032686 | Acute upper respiratory tract infection | NA | 0 | NA | NA | Unchanged | Improved | Unchanged |
| 1031572 | Acute upper respiratory tract infection | NA | 0 | NA | NA | Unchanged | Improved | Unchanged |
| 1031555 | Acute upper respiratory tract infection | NA | 1 | Herpes simplex virus type 1 | 0 | Escalated | Improved | Medications changed |
| 1031132 | Acute upper respiratory tract infection | NA | 0 | NA | NA | Unchanged | Improved | Unchanged |
| 1031125 | Acute upper respiratory tract infection | NA | 1 | Coxsackievirus type A10 | NA | Unchanged | Improved | Unchanged |
| 1031050 | Acute upper respiratory tract infection | NA | 0 | NA | NA | Unchanged | Improved | Unchanged |
| 1030761 | Acute upper respiratory tract infection | NA | 1 | Influenza A virus | NA | Unchanged | Improved | Unchanged |
| 1030724 | Acute upper respiratory tract infection | NA | 1 | Enterovirus group A | NA | Unchanged | Improved | Unchanged |
| 1030618 | Acute upper respiratory tract infection | NA | 1 | Epstein-Barr virus | NA | Unchanged | Improved | Unchanged |
| 1030384 | Acute upper respiratory tract infection | NA | 0 | NA | NA | Unchanged | Improved | Unchanged |
| 1029826 | Acute upper respiratory tract infection | NA | 1 | Rhinovirus type A | NA | Escalated | Improved | Medication added |
| 1029455 | Acute upper respiratory tract infection | NA | 0 | NA | NA | De-escalated | Improved | Discontinue medication |
| 1029259 | Acute upper respiratory tract infection | NA | 0 | NA | NA | De-escalated | Improved | Discharge |
| 1028993 | Acute upper respiratory tract infection | NA | 1 | Human coronavirus OC43 | NA | De-escalated | Improved | Discharge |
| 1028429 | Acute upper respiratory tract infection | NA | 0 | NA | NA | De-escalated | Improved | Discharge |
| 1024910 | Acute upper respiratory tract infection | NA | 0 | NA | 0 | Unchanged | Improved | Unchanged |
| 1038890 | Acute tonsillitis | NA | 1 | Staphylococcus aureus | NA | Unchanged | Improved | Unchanged |
| 1031726 | Acute tonsillitis | NA | 1 | Human adenovirus type 1 | NA | De-escalated | Improved | Discharge |
| 1045928 | Acute suppurative tonsillitis | NA | 1 | Human adenovirus type 3; Influenza A virus; Bordetella pertussis | 1 | Escalated | Improved | Medications changed |
| 1042389 | Acute suppurative tonsillitis | NA | 1 | Human adenovirus type 3; Mycoplasma pneumoniae; Herpes simplex virus type 1 | 1 | De-escalated | Improved | Discontinue medication |
| 1042254 | Acute suppurative tonsillitis | NA | 1 | Human adenovirus type 3 | 0 | Escalated | Improved | Nebulization of interferon |
| 1041912 | Acute suppurative tonsillitis | NA | 1 | Coxsackievirus type A6; Streptococcus pyogenes; Rhinovirus type C | NA | Unchanged | Improved | Unchanged |
| 1032327 | Acute suppurative tonsillitis | NA | 1 | Human adenovirus group C | NA | Unchanged | Improved | Unchanged |
| 1032259 | Acute suppurative tonsillitis | NA | 1 | Human adenovirus group C; Human parainfluenza virus type 4; Streptococcus pneumoniae; Haemophilus influenzae | 0 | Escalated | Improved | Medication added |
| 1030615 | Acute suppurative tonsillitis | NA | 1 | Human coronavirus OC43; Haemophilus influenzae | NA | De-escalated | Improved | Discharge |
| 1022201 | Acute suppurative tonsillitis | NA | 1 | Human adenovirus type 2 | NA | Unchanged | Improved | Unchanged |
| 1042259 | Acute laryngobronchitis | NA | 1 | Influenza A virus H3N2 | 0 | Escalated | Improved | Nebulization of interferon |
| 1020148 | Acute bronchitis, herpangina | NA | 1 | Coxsackievirus type A6 | NA | Unchanged | Improved | Unchanged |
| 1024190 | Acute bronchitis, acute myositis | NA | 1 | Influenza A virus H1N1 | 0 | De-escalated | Improved | Discharge |
| 980054 | Acute bronchitis | NA | 0 | NA | 0 | Unchanged | Unchanged | Unchanged |
| 973091 | Acute bronchitis | NA | 1 | Coxsackievirus type A5 | 1 | De-escalated | Improved | Others |
| 952541 | Acute bronchitis | NA | 1 | Bordetella pertussis | 1 | Unchanged | Improved | Unchanged |
| 948914 | Acute bronchitis | NA | 1 | Streptococcus agalactiae; Human parainfluenza virus type 3; Human bocavirus type 1; Coxsackievirus type A10 | 1 | De-escalated | Improved | Discontinue medication |
| 947153 | Acute bronchitis | NA | 1 | Human parainfluenza virus type 1; Streptococcus pyogenes; Epstein-Barr virus | 0 | Escalated | Improved | Medication added |
| 931421 | Acute bronchitis | NA | 1 | Rhinovirus type A | 1 | De-escalated | Improved | Discontinue medication |
| 902408 | Acute bronchitis | NA | 1 | Coxsackievirus type A6; Rhinovirus type A; Haemophilus influenzae | 1 | De-escalated | Improved | Discharge |
| 895464 | Acute bronchitis | NA | 1 | Human coronavirus OC43; Influenza A virus H3N2; Herpes simplex virus type 1 | NA | De-escalated | Improved | Discontinue medication |
| 874435 | Acute bronchitis | NA | 1 | Herpes simplex virus type 1 | 0 | De-escalated | Improved | Discontinue medication |
| 824505 | Acute bronchitis | NA | 1 | Influenza A virus H3N2; Streptococcus pneumoniae | 1 | De-escalated | Improved | Discharge |
| 1045765 | Acute bronchitis | NA | 1 | Influenza A virus H3N2 | NA | Escalated | Improved | Medication added |
| 1043402 | Acute bronchitis | NA | 1 | Influenza A virus | 0 | Escalated | Improved | Medication added |
| 1043111 | Acute bronchitis | NA | 1 | Influenza A virus; Rhinovirus type A; Bordetella pertussis | 1 | Escalated | Improved | Medication added |
| 1042915 | Acute bronchitis | NA | 1 | Influenza A virus H3N2; Rhinovirus type C; Haemophilus influenzae; Streptococcus pneumoniae | 0 | De-escalated | Improved | Discontinue medication |
| 1042552 | Acute bronchitis | NA | 1 | Human adenovirus type 3 | 1 | De-escalated | Improved | Discontinue medication |
| 1042168 | Acute bronchitis | NA | 1 | Human adenovirus type 3 | 1 | Escalated | Improved | Nebulization of interferon |
| 1038547 | Acute bronchitis | NA | 1 | Rhinovirus type A | 1 | Escalated | Improved | Medications changed |
| 1038513 | Acute bronchitis | NA | 1 | Human adenovirus type 3 | 1 | Escalated | Improved | Medication added |
| 1037498 | Acute bronchitis | NA | 1 | Influenza A virus H3N2 | 1 | Unchanged | Improved | Unchanged |
| 1035662 | Acute bronchitis | NA | 1 | Human parainfluenza virus type 3; Streptococcus pneumoniae | 1 | Unchanged | Improved | Unchanged |
| 1035378 | Acute bronchitis | NA | 1 | Human adenovirus type 3 | NA | Unchanged | Improved | Unchanged |
| 1035063 | Acute bronchitis | NA | 0 | NA | 1 | Unchanged | Improved | Unchanged |
| 1034703 | Acute bronchitis | NA | 1 | Human metapneumovirus | 1 | Unchanged | Improved | Unchanged |
| 1034302 | Acute bronchitis | NA | 1 | Human adenovirus type 2 | 1 | Unchanged | Improved | Unchanged |
| 1034289 | Acute bronchitis | NA | 1 | Coxsackievirus type A6; Streptococcus pneumoniae; Haemophilus influenzae | NA | Unchanged | Improved | Unchanged |
| 1034211 | Acute bronchitis | NA | 1 | Human bocavirus type 1 | NA | De-escalated | Improved | Discontinue medication |
| 1034200 | Acute bronchitis | NA | 1 | Rhinovirus type A; Bordetella pertussis; Haemophilus influenzae; Streptococcus pneumoniae | 0 | Unchanged | Improved | Unchanged |
| 1033924 | Acute bronchitis | NA | 1 | Human metapneumovirus | 1 | Unchanged | Improved | Unchanged |
| 1033594 | Acute bronchitis | NA | 1 | Rhinovirus type A; Streptococcus pneumoniae | NA | Unchanged | Improved | Unchanged |
| 1033509 | Acute bronchitis | NA | 0 | NA | 0 | De-escalated | Improved | Discontinue medication |
| 1033505 | Acute bronchitis | NA | 1 | Human parainfluenza virus type 3; Human bocavirus type 1; Streptococcus pneumoniae | 0 | Unchanged | Improved | Unchanged |
| 1033338 | Acute bronchitis | NA | 1 | Coxsackievirus type A5 | 0 | Unchanged | Improved | Unchanged |
| 1032889 | Acute bronchitis | NA | 1 | Human parainfluenza virus type 1 | NA | Unchanged | Improved | Unchanged |
| 1032810 | Acute bronchitis | NA | 1 | Human bocavirus type 1 | 0 | De-escalated | Improved | Discontinue medication |
| 1032730 | Acute bronchitis | NA | 1 | Coxsackievirus type A10 | NA | Escalated | Improved | Medication added |
| 1032689 | Acute bronchitis | NA | 1 | Human parainfluenza virus type 3; Rhinovirus type A; Moraxella catarrhalis | 0 | Escalated | Improved | Medication added |
| 1032189 | Acute bronchitis | NA | 1 | Human bocavirus type 1 | 0 | Unchanged | Improved | Unchanged |
| 1032162 | Acute bronchitis | NA | 1 | Human adenovirus type 2 | NA | Escalated | Improved | Nebulization of interferon |
| 1031548 | Acute bronchitis | NA | 1 | Human parainfluenza virus type 3 | 0 | Escalated | Improved | Medication added |
| 1030151 | Acute bronchitis | NA | 1 | Human adenovirus type 1 | 0 | Escalated | Improved | Medication added |
| 1029888 | Acute bronchitis | NA | 1 | Human respiratory syncytial virus type A | 0 | Unchanged | Improved | Unchanged |
| 1029880 | Acute bronchitis | NA | 1 | Rhinovirus type A | NA | Unchanged | Improved | Unchanged |
| 1029815 | Acute bronchitis | NA | 1 | Human adenovirus type 2; Human metapneumovirus | 0 | De-escalated | Improved | Discontinue medication |
| 1029603 | Acute bronchitis | NA | 1 | Human coronavirus OC43 | 0 | Unchanged | Improved | Unchanged |
| 1029341 | Acute bronchitis | NA | 0 | NA | NA | Escalated | Improved | Medication added |
| 1028972 | Acute bronchitis | NA | 1 | Human coronavirus OC43; Streptococcus pneumoniae | NA | De-escalated | Improved | Discharge |
| 1027860 | Acute bronchitis | NA | 0 | NA | 0 | Escalated | Improved | Medication added |
| 1027767 | Acute bronchitis | NA | 1 | Human respiratory syncytial virus type A | 0 | Unchanged | Improved | Unchanged |
| 1026668 | Acute bronchitis | NA | 1 | Coxsackievirus type A6 | NA | Unchanged | Improved | Unchanged |
| 1026195 | Acute bronchitis | NA | 1 | Mycoplasma pneumoniae | 0 | De-escalated | Improved | Discharge |
| 1024919 | Acute bronchitis | NA | 1 | Human metapneumovirus; Cytomegalovirus | NA | Unchanged | Improved | Unchanged |
| 1024306 | Acute bronchitis | NA | 1 | Influenza A virus H3N2; Streptococcus pyogenes; Streptococcus pneumoniae | 0 | De-escalated | Improved | Discontinue medication |
| 1024095 | Acute bronchitis | NA | 1 | Human respiratory syncytial virus type A; Rhinovirus type C; Moraxella catarrhalis | 0 | De-escalated | Improved | Discharge |
| 1024043 | Acute bronchitis | NA | 1 | Human respiratory syncytial virus type B; Human parainfluenza virus type 3 | 0 | De-escalated | Improved | Reduce the amount |
| 1023835 | Acute bronchitis | NA | 1 | Human respiratory syncytial virus type A; Influenza A virus H1N1 ; Streptococcus pneumoniae | NA | Escalated | Improved | Medication added |
| 1021620 | Acute bronchitis | NA | 1 | Human coronavirus OC43 | NA | Unchanged | Improved | Unchanged |
| 1002057 | Acute bronchitis | NA | 1 | Human coronavirus OC43; Streptococcus pneumoniae; Moraxella catarrhalis |  | Escalated | Improved | Medication added |
| 1045818 | Acute asthmatic bronchitis | NA | 1 | Rhinovirus type B | 0 | De-escalated | Improved | Discontinue medication |
| 1023441 | Acute asthmatic bronchitis | NA | 1 | Influenza A virus H3N2; Mycoplasma pneumoniae; Streptococcus pneumoniae | 0 | De-escalated | Improved | Discharge |
| 1005193 | Viral meningitis, acute upper respiratory tract infection | Mycoplasma pneumoniae | 1 | Rhinovirus type A | 1 | Unchanged | Improved | Unchanged |
| 1028012 | Severe pneumonia, herpetic angina | Mycoplasma pneumoniae | 1 | Human coronavirus OC43 | 1 | De-escalated | Improved | Discontinue medication |
| 981828 | Severe pneumonia, acute suppurative tonsillitis | Mycoplasma pneumoniae | 1 | Mycoplasma pneumoniae; Haemophilus influenzae | 1 | Escalated | Improved | Prolong the duration of treatment |
| 1045791 | Severe pneumonia | Mycoplasma pneumoniae | 1 | Mycoplasma pneumoniae | 1 | Escalated | Improved | Medication added |
| 1045546 | Severe pneumonia | Mycoplasma pneumoniae | 1 | Human respiratory syncytial virus type B; Mycoplasma pneumoniae | 1 | Escalated | Improved | Medications changed |
| 1044534 | Severe pneumonia | Mycoplasma pneumoniae | 1 | Influenza A virus H3N2; Mycoplasma pneumoniae; Epstein-Barr virus | 1 | De-escalated | Improved | Reduce the amount |
| 1044245 | Severe pneumonia | Mycoplasma pneumoniae | 1 | Mycoplasma pneumoniae | 1 | De-escalated | Improved | Reduce the amount |
| 1043885 | Severe pneumonia | Mycoplasma pneumoniae | 1 | Mycoplasma pneumoniae | 1 | De-escalated | Improved | Reduce the amount |
| 1043112 | Severe pneumonia | Mycoplasma pneumoniae | 1 | Influenza A virus H3N2 | 1 | De-escalated | Improved | Discontinue medication |
| 1043035 | Severe pneumonia | Mycoplasma pneumoniae | 1 | Influenza A virus H3N2; Mycoplasma pneumoniae; Rhinovirus type C | 1 | Escalated | Improved | Medication added |
| 1043010 | Severe pneumonia | Mycoplasma pneumoniae | 1 | Mycoplasma pneumoniae | 1 | Escalated | Improved | Medications changed |
| 1042686 | Severe pneumonia | Mycoplasma pneumoniae | 1 | Mycoplasma pneumoniae | 1 | Escalated | Improved | Medication added |
| 1042599 | Severe pneumonia | Mycoplasma pneumoniae | 1 | Mycoplasma pneumoniae; Rhinovirus type C | 1 | De-escalated | Improved | Discontinue medication |
| 1042597 | Severe pneumonia | Mycoplasma pneumoniae | 1 | Mycoplasma pneumoniae | 1 | De-escalated | Improved | Reduce the amount |
| 1042352 | Severe pneumonia | Mycoplasma pneumoniae | 1 | Mycoplasma pneumoniae | 1 | De-escalated | Improved | Reduce the amount |
| 1042180 | Severe pneumonia | Mycoplasma pneumoniae | 1 | Streptococcus pneumoniae | 1 | Unchanged | Aggravated | Unchanged |
| 1041624 | Severe pneumonia | Mycoplasma pneumoniae | 1 | Mycoplasma pneumoniae; Herpes simplex virus type 1; Streptococcus pneumoniae | 1 | Escalated | Improved | Medication added |
| 1041340 | Severe pneumonia | Mycoplasma pneumoniae | 1 | Mycoplasma pneumoniae; Rhinovirus type B | 1 | De-escalated | Improved | Reduce the amount |
| 1041330 | Severe pneumonia | Mycoplasma pneumoniae | 1 | Mycoplasma pneumoniae | 1 | De-escalated | Improved | Reduce the amount |
| 1040765 | Severe pneumonia | Mycoplasma pneumoniae | 1 | Mycoplasma pneumoniae; Rhinovirus type B | 1 | Escalated | Improved | Prolong the duration of treatment |
| 1040520 | Severe pneumonia | Mycoplasma pneumoniae | 1 | Mycoplasma pneumoniae | 1 | De-escalated | Improved | Reduce the amount |
| 1040296 | Severe pneumonia | Mycoplasma pneumoniae | 1 | Human coronavirus OC43; Mycoplasma pneumoniae | 1 | Unchanged | Improved | Unchanged |
| 1040234 | Severe pneumonia | Mycoplasma pneumoniae | 1 | Mycoplasma pneumoniae | 1 | Unchanged | Improved | Unchanged |
| 1040152 | Severe pneumonia | Mycoplasma pneumoniae | 1 | Mycoplasma pneumoniae | 1 | Unchanged | Improved | Unchanged |
| 1040133 | Severe pneumonia | Mycoplasma pneumoniae | 1 | Mycoplasma pneumoniae; Streptococcus pneumoniae | 1 | Unchanged | Aggravated | Unchanged |
| 1039942 | Severe pneumonia | Mycoplasma pneumoniae | 1 | Mycoplasma pneumoniae | 1 | Unchanged | Unchanged | Unchanged |
| 1039940 | Severe pneumonia | Mycoplasma pneumoniae | 1 | Coxsackievirus type A6 | 1 | Escalated | Improved | Nebulization of interferon |
| 1039882 | Severe pneumonia | Mycoplasma pneumoniae | 1 | Mycoplasma pneumoniae | 1 | Escalated | Improved | Others |
| 1039505 | Severe pneumonia | Mycoplasma pneumoniae | 1 | Mycoplasma pneumoniae | 1 | Escalated | Improved | Prolong the duration of treatment |
| 1039428 | Severe pneumonia | Mycoplasma pneumoniae | 1 | Human bocavirus type 1 | 1 | Escalated | Improved | Medication added |
| 1039274 | Severe pneumonia | Mycoplasma pneumoniae | 1 | Mycoplasma pneumoniae | 1 | Escalated | Improved | Prolong the duration of treatment |
| 1039011 | Severe pneumonia | Mycoplasma pneumoniae | 1 | Mycoplasma pneumoniae; Rhinovirus type A | 1 | Escalated | Improved | Prolong the duration of treatment |
| 1038929 | Severe pneumonia | Mycoplasma pneumoniae | 1 | Mycoplasma pneumoniae | 1 | Escalated | Improved | Others |
| 1036205 | Severe pneumonia | Mycoplasma pneumoniae | 1 | Mycoplasma pneumoniae | 1 | Escalated | Improved | Others |
| 1036118 | Severe pneumonia | Mycoplasma pneumoniae | 1 | Mycoplasma pneumoniae; Cytomegalovirus | 1 | De-escalated | Improved | Others |
| 1032812 | Severe pneumonia | Mycoplasma pneumoniae | 1 | Human metapneumovirus; Streptococcus pneumoniae | 1 | Escalated | Improved | Medication added |
| 1026733 | Severe pneumonia | Mycoplasma pneumoniae | 1 | Human respiratory syncytial virus type A; Streptococcus pneumoniae | 1 | Escalated | Improved | Others |
| 991823 | Severe pneumonia | Mycoplasma pneumoniae | 0 | NA | 1 | De-escalated | Improved | Discontinue medication |
| 914412 | Severe pneumonia | Mycoplasma pneumoniae | 1 | Mycoplasma pneumoniae | 1 | Unchanged | Improved | Unchanged |
| 835529 | Severe pneumonia | Mycoplasma pneumoniae | 1 | Mycoplasma pneumoniae | 1 | Escalated | Improved | Medications changed |
| 1038753 | Refractory Mycoplasma Pneumonia | Mycoplasma pneumoniae | 1 | Mycoplasma pneumoniae | 1 | Escalated | Improved | Medications changed |
| 1028532 | Pneumonia-mycoplasma pneumoniae infection, acute laryngitis | Mycoplasma pneumoniae | 1 | Human respiratory syncytial virus type B; Streptococcus pneumoniae | 1 | De-escalated | Improved | Discontinue medication |
| 1042818 | Pneumonia-mycoplasma pneumoniae infection | Mycoplasma pneumoniae | 1 | Mycoplasma pneumoniae | 1 | De-escalated | Improved | Reduce the amount |
| 1042713 | Pneumonia-mycoplasma pneumoniae infection | Mycoplasma pneumoniae | 1 | Mycoplasma pneumoniae | 1 | Unchanged | Improved | Unchanged |
| 1042172 | Pneumonia-mycoplasma pneumoniae infection | Mycoplasma pneumoniae | 1 | Influenza A virus H3N2; Mycoplasma pneumoniae; Rhinovirus; Bordetella pertussis | 1 | Escalated | Improved | Medication added |
| 1041944 | Pneumonia-mycoplasma pneumoniae infection | Mycoplasma pneumoniae | 1 | Mycoplasma pneumoniae | 1 | Escalated | Improved | Medication added |
| 1041332 | Pneumonia-mycoplasma pneumoniae infection | Mycoplasma pneumoniae | 1 | Mycoplasma pneumoniae | 1 | Escalated | Improved | Medication added |
| 1040314 | Pneumonia-mycoplasma pneumoniae infection | Mycoplasma pneumoniae | 1 | Mycoplasma pneumoniae | 1 | Unchanged | Improved | Unchanged |
| 1039591 | Pneumonia-mycoplasma pneumoniae infection | Mycoplasma pneumoniae | 1 | Mycoplasma pneumoniae | 1 | Escalated | Improved | Others |
| 1037830 | Pneumonia-mycoplasma pneumoniae infection | Mycoplasma pneumoniae | 1 | Influenza A virus H3N2 | 1 | Escalated | Improved | Others |
| 1033337 | Pneumonia-mycoplasma pneumoniae infection | Mycoplasma pneumoniae | 1 | Human bocavirus type 1 | 1 | Escalated | Improved | Medication added |
| 1032589 | Pneumonia-mycoplasma pneumoniae infection | Mycoplasma pneumoniae | 1 | Mycoplasma pneumoniae | 1 | Escalated | Improved | Prolong the duration of treatment |
| 1032413 | Pneumonia-mycoplasma pneumoniae infection | Mycoplasma pneumoniae | 1 | Human parainfluenza virus type 3; Streptococcus pneumoniae | 1 | Escalated | Improved | Prolong the duration of treatment |
| 1031041 | Pneumonia-mycoplasma pneumoniae infection | Mycoplasma pneumoniae | 1 | Mycoplasma pneumoniae; Haemophilus influenzae | 1 | Escalated | Improved | Prolong the duration of treatment |
| 1023009 | Pneumonia-mycoplasma pneumoniae infection | Mycoplasma pneumoniae | 1 | Mycoplasma pneumoniae | 1 | De-escalated | Improved | Discontinue medication |
| 1022428 | Pneumonia-mycoplasma pneumoniae infection | Mycoplasma pneumoniae | 1 | Human metapneumovirus; Human respiratory syncytial virus type A; Streptococcus pneumoniae | 1 | Escalated | Improved | Medication added |
| 992214 | Pneumonia-mycoplasma pneumoniae infection | Mycoplasma pneumoniae | 1 | Human metapneumovirus; Coxsackievirus type A6 | 1 | Escalated | Improved | Others |
| 989701 | Pneumonia-mycoplasma pneumoniae infection | Mycoplasma pneumoniae | 1 | Influenza A virus | 1 | De-escalated | Improved | Others |
| 866088 | Pneumonia-mycoplasma pneumoniae infection | Mycoplasma pneumoniae | 1 | Human parainfluenza virus type 3; Mycoplasma pneumoniae | 1 | Escalated | Improved | Medication added |
| 1045889 | Pneumonia, suppurative tonsillitis | Mycoplasma pneumoniae | 1 | Human adenovirus type 3 | 1 | Escalated | Improved | Medication added |
| 1045589 | Pneumonia, purulent tonsillitis, rhinitis | Mycoplasma pneumoniae | 1 | Mycoplasma pneumoniae; Epstein-Barr virus | 1 | De-escalated | Improved | Reduce the amount |
| 1007122 | Pneumonia, laryngitis | Mycoplasma pneumoniae | 1 | Human coronavirus HKU1; Human coronavirus 229E; Rhinovirus type B | 1 | De-escalated | Improved | Discharge |
| 1028009 | Pneumonia, herpangina | Mycoplasma pneumoniae | 1 | Human respiratory syncytial virus type A | 1 | Escalated | Improved | Medication added |
| 1044452 | Pneumonia, bullae | Mycoplasma pneumoniae | 1 | Human metapneumovirus; Streptococcus pneumoniae; Moraxella catarrhalis | 1 | De-escalated | Improved | Reduce the amount |
| 984935 | Pneumonia, acute suppurative tonsillitis | Mycoplasma pneumoniae | 1 | Human parainfluenza virus type 4; Coxsackievirus type A10 | 1 | Unchanged | Improved | Unchanged |
| 901984 | Pneumonia, acute suppurative tonsillitis | Mycoplasma pneumoniae | 1 | Mycoplasma pneumoniae; Streptococcus pneumoniae | 1 | Escalated | Improved | Prolong the duration of treatment |
| 1045918 | Pneumonia | Mycoplasma pneumoniae | 1 | Mycoplasma pneumoniae; Herpes simplex virus type 1; Rhinovirus type A | 1 | Escalated | Improved | Medication added |
| 1045906 | Pneumonia | Mycoplasma pneumoniae | 1 | Human metapneumovirus; Human respiratory syncytial virus type B; Mycoplasma pneumoniae | 1 | De-escalated | Improved | Discontinue medication |
| 1045817 | Pneumonia | Mycoplasma pneumoniae | 1 | Human respiratory syncytial virus type B; Mycoplasma pneumoniae; Bordetella pertussis | 1 | De-escalated | Improved | Discontinue medication |
| 1045152 | Pneumonia | Mycoplasma pneumoniae | 1 | Mycoplasma pneumoniae | 1 | Escalated | Improved | Medication added |
| 1044938 | Pneumonia | Mycoplasma pneumoniae | 1 | Mycoplasma pneumoniae | 1 | De-escalated | Improved | Discontinue medication |
| 1044737 | Pneumonia | Mycoplasma pneumoniae | 1 | Human adenovirus type 3; Mycoplasma pneumoniae; Epstein-Barr virus | 1 | De-escalated | Improved | Reduce the amount |
| 1044630 | Pneumonia | Mycoplasma pneumoniae | 1 | Mycoplasma pneumoniae; Epstein-Barr virus | 1 | Escalated | Improved | Medication added |
| 1044432 | Pneumonia | Mycoplasma pneumoniae | 1 | Human adenovirus type 3; Influenza A virus; Mycoplasma pneumoniae | 1 | De-escalated | Improved | Others |
| 1044286 | Pneumonia | Mycoplasma pneumoniae | 1 | Human adenovirus type 3; Mycoplasma pneumoniae | 1 | Escalated | Improved | Medication added |
| 1044257 | Pneumonia | Mycoplasma pneumoniae | 1 | Human adenovirus type 3 | 1 | Escalated | Improved | Medication added |
| 1044248 | Pneumonia | Mycoplasma pneumoniae | 1 | Human respiratory syncytial virus type B | 1 | De-escalated | Improved | Others |
| 1043855 | Pneumonia | Mycoplasma pneumoniae | 1 | Mycoplasma pneumoniae | 1 | De-escalated | Improved | Reduce the amount |
| 1043496 | Pneumonia | Mycoplasma pneumoniae | 1 | Human bocavirus type 1; Influenza A virus H3N2 | 1 | De-escalated | Improved | Discharge |
| 1043415 | Pneumonia | Mycoplasma pneumoniae | 1 | Mycoplasma pneumoniae | 1 | De-escalated | Improved | Discontinue medication |
| 1043212 | Pneumonia | Mycoplasma pneumoniae | 1 | Human adenovirus type 3; Influenza A virus H3N2; Mycoplasma pneumoniae | 1 | De-escalated | Improved | Discontinue medication |
| 1043192 | Pneumonia | Mycoplasma pneumoniae | 1 | Mycoplasma pneumoniae | 1 | Escalated | Improved | Medication added |
| 1043123 | Pneumonia | Mycoplasma pneumoniae | 1 | Haemophilus influenzae | 1 | Escalated | Improved | Medications changed |
| 1043041 | Pneumonia | Mycoplasma pneumoniae | 1 | Streptococcus dysgalactiae | 1 | De-escalated | Improved | Discontinue medication |
| 1043009 | Pneumonia | Mycoplasma pneumoniae | 1 | Mycoplasma pneumoniae | 1 | Unchanged | Improved | Unchanged |
| 1042934 | Pneumonia | Mycoplasma pneumoniae | 1 | Mycoplasma pneumoniae | 1 | Escalated | Improved | Medications changed |
| 1042835 | Pneumonia | Mycoplasma pneumoniae | 1 | Influenza A virus; Streptococcus pneumoniae | 1 | De-escalated | Improved | Reduce the amount |
| 1042428 | Pneumonia | Mycoplasma pneumoniae | 1 | Influenza A virus H3N2; Streptococcus pneumoniae; Moraxella catarrhalis | 1 | Escalated | Improved | Medication added |
| 1042187 | Pneumonia | Mycoplasma pneumoniae | 1 | Rhinovirus type C; Haemophilus influenzae | 1 | De-escalated | Improved | Discontinue medication |
| 1042143 | Pneumonia | Mycoplasma pneumoniae | 1 | Human adenovirus type 3; Mycoplasma pneumoniae; Rhinovirus type C | 1 | Escalated | Improved | Nebulization of interferon |
| 1041695 | Pneumonia | Mycoplasma pneumoniae | 1 | Mycoplasma pneumoniae | 1 | De-escalated | Improved | Reduce the amount |
| 1041652 | Pneumonia | Mycoplasma pneumoniae | 1 | Mycoplasma pneumoniae | 1 | Escalated | Improved | Medication added |
| 1041428 | Pneumonia | Mycoplasma pneumoniae | 1 | Human coronavirus OC43; Rhinovirus type B | 1 | Unchanged | Improved | Unchanged |
| 1041408 | Pneumonia | Mycoplasma pneumoniae | 1 | Influenza A virus H3N2 | 1 | Unchanged | Improved | Unchanged |
| 1041320 | Pneumonia | Mycoplasma pneumoniae | 1 | Mycoplasma pneumoniae | 1 | Escalated | Improved | Medications changed |
| 1041135 | Pneumonia | Mycoplasma pneumoniae | 1 | Mycoplasma pneumoniae | 1 | Unchanged | Improved | Unchanged |
| 1041100 | Pneumonia | Mycoplasma pneumoniae | 1 | Human adenovirus type 3; Mycoplasma pneumoniae | 1 | Escalated | Improved | Nebulization of interferon |
| 1040848 | Pneumonia | Mycoplasma pneumoniae | 1 | Mycoplasma pneumoniae; Rhinovirus type B | 1 | Escalated | Improved | Prolong the duration of treatment |
| 1040793 | Pneumonia | Mycoplasma pneumoniae | 1 | Mycoplasma pneumoniae; Haemophilus influenzae | 1 | Escalated | Improved | Prolong the duration of treatment |
| 1040762 | Pneumonia | Mycoplasma pneumoniae | 1 | Mycoplasma pneumoniae; Rhinovirus type C; Streptococcus pneumoniae | 1 | De-escalated | Improved | Discontinue medication |
| 1040724 | Pneumonia | Mycoplasma pneumoniae | 1 | Mycoplasma pneumoniae | 1 | De-escalated | Improved | Discontinue medication |
| 1040625 | Pneumonia | Mycoplasma pneumoniae | 1 | Mycoplasma pneumoniae | 1 | De-escalated | Improved | Reduce the amount |
| 1040582 | Pneumonia | Mycoplasma pneumoniae | 1 | Mycoplasma pneumoniae | 1 | Escalated | Improved | Medication added |
| 1040444 | Pneumonia | Mycoplasma pneumoniae | 1 | Mycoplasma pneumoniae | 1 | De-escalated | Improved | Discontinue medication |
| 1039982 | Pneumonia | Mycoplasma pneumoniae | 1 | Mycoplasma pneumoniae; Rhinovirus type B | 1 | Escalated | Improved | Medication added |
| 1039682 | Pneumonia | Mycoplasma pneumoniae | 1 | Human parainfluenza virus type 4; Rhinovirus type A; Bordetella pertussis | 1 | Escalated | Improved | Medication added |
| 1039358 | Pneumonia | Mycoplasma pneumoniae | 1 | Mycoplasma pneumoniae | 1 | De-escalated | Improved | Reduce the amount |
| 1038940 | Pneumonia | Mycoplasma pneumoniae | 1 | Mycoplasma pneumoniae | 1 | Escalated | Improved | Prolong the duration of treatment |
| 1035151 | Pneumonia | Mycoplasma pneumoniae | 1 | Mycoplasma pneumoniae | 1 | Escalated | Improved | Prolong the duration of treatment |
| 1030201 | Pneumonia | Mycoplasma pneumoniae | 1 | Human parainfluenza virus type 3 | 1 | Unchanged | Improved | Unchanged |
| 1030153 | Pneumonia | Mycoplasma pneumoniae | 1 | Mycoplasma pneumoniae | 1 | De-escalated | Improved | Discontinue medication |
| 1027213 | Pneumonia | Mycoplasma pneumoniae | 1 | Human respiratory syncytial virus type A | 1 | Escalated | Improved | Prolong the duration of treatment |
| 1005156 | Pneumonia | Mycoplasma pneumoniae | 1 | Mycoplasma pneumoniae; Rhinovirus type A; Moraxella catarrhalis | 1 | Escalated | Improved | Prolong the duration of treatment |
| 990617 | Pneumonia | Mycoplasma pneumoniae | 1 | Influenza A virus H3N2 | 1 | Escalated | Improved | Others |
| 986464 | Pneumonia | Mycoplasma pneumoniae | 1 | Human metapneumovirus | 1 | De-escalated | Improved | Discontinue medication |
| 978338 | Pneumonia | Mycoplasma pneumoniae | 1 | Mycoplasma pneumoniae | 1 | Escalated | Improved | Others |
| 959951 | Pneumonia | Mycoplasma pneumoniae | 1 | Human parainfluenza virus type 1; Mycoplasma pneumoniae | 1 | De-escalated | Improved | Discontinue medication |
| 1044435 | Febrile seizure, acute bronchitis | Mycoplasma pneumoniae | 1 | Influenza A virus H3N2; Streptococcus pneumoniae | 0 | Escalated | Improved | Medication added |
| 1044346 | Febrile seizure, acute bronchitis | Mycoplasma pneumoniae | 1 | Influenza A virus H3N2; Moraxella catarrhalis | 0 | Escalated | Improved | Medication added |
| 1043031 | Febrile convulsions | Mycoplasma pneumoniae | 1 | Influenza A virus H3N2 | 0 | Escalated | Improved | Medication added |
| 1045150 | Bronchopneumonia, whooping cough | Mycoplasma pneumoniae | 1 | Human parainfluenza virus type 1; Bordetella pertussis | 1 | Unchanged | Improved | Unchanged |
| 1027501 | Bronchopneumonia, systemic inflammatory response syndrome | Mycoplasma pneumoniae | 1 | Human respiratory syncytial virus type A; Staphylococcus aureus; Streptococcus pneumoniae | 1 | De-escalated | Improved | Discontinue medication |
| 939842 | Bronchopneumonia, herpangina | Mycoplasma pneumoniae | 1 | Human metapneumovirus; Coxsackievirus type A6 | 1 | Escalated | Improved | Medication added |
| 1044116 | Bronchopneumonia | Mycoplasma pneumoniae | 1 | Human adenovirus type 3; Epstein-Barr virus | 1 | Unchanged | Improved | Unchanged |
| 1042667 | Bronchopneumonia | Mycoplasma pneumoniae | 1 | Human metapneumovirus | 1 | Unchanged | Improved | Unchanged |
| 1042449 | Bronchopneumonia | Mycoplasma pneumoniae | 0 | NA | 1 | Unchanged | Improved | Unchanged |
| 1042157 | Bronchopneumonia | Mycoplasma pneumoniae | 1 | Human adenovirus type 3 | 1 | Escalated | Improved | Nebulization of interferon |
| 1042156 | Bronchopneumonia | Mycoplasma pneumoniae | 1 | Epstein-Barr virus | 1 | Unchanged | Improved | Unchanged |
| 1041327 | Bronchopneumonia | Mycoplasma pneumoniae | 1 | Mycoplasma pneumoniae; Epstein-Barr virus | 1 | De-escalated | Improved | Discharge |
| 1041313 | Bronchopneumonia | Mycoplasma pneumoniae | 1 | Influenza A virus H3N2 | 1 | Unchanged | Improved | Unchanged |
| 1041129 | Bronchopneumonia | Mycoplasma pneumoniae | 1 | Mycoplasma pneumoniae | 1 | De-escalated | Improved | Reduce the amount |
| 1040808 | Bronchopneumonia | Mycoplasma pneumoniae | 1 | Mycoplasma pneumoniae | 1 | Escalated | Improved | Prolong the duration of treatment |
| 1040546 | Bronchopneumonia | Mycoplasma pneumoniae | 1 | Mycoplasma pneumoniae; Herpes simplex virus type 1; Rhinovirus type A | 1 | Unchanged | Improved | Unchanged |
| 1040411 | Bronchopneumonia | Mycoplasma pneumoniae | 1 | Mycoplasma pneumoniae | 1 | Escalated | Improved | Medication added |
| 1040056 | Bronchopneumonia | Mycoplasma pneumoniae | 1 | Mycoplasma pneumoniae; Epstein-Barr virus; Epstein-Barr virus | 1 | Escalated | Improved | Medication added |
| 1040040 | Bronchopneumonia | Mycoplasma pneumoniae | 1 | Human adenovirus type 3; Herpes simplex virus type 1 | 1 | De-escalated | Improved | Discontinue medication |
| 1039936 | Bronchopneumonia | Mycoplasma pneumoniae | 1 | Rhinovirus type C | 1 | De-escalated | Improved | Discontinue medication |
| 1036680 | Bronchopneumonia | Mycoplasma pneumoniae | 1 | Mycoplasma pneumoniae | 1 | Unchanged | Improved | Unchanged |
| 1036226 | Bronchopneumonia | Mycoplasma pneumoniae | 1 | Human parainfluenza virus type 3; Epstein-Barr virus | 1 | Unchanged | Improved | Unchanged |
| 1035159 | Bronchopneumonia | Mycoplasma pneumoniae | 1 | Human metapneumovirus; Human parainfluenza virus type 3 | 1 | Escalated | Improved | Prolong the duration of treatment |
| 1035047 | Bronchopneumonia | Mycoplasma pneumoniae | 1 | Human parainfluenza virus type 1; Human bocavirus type 1; Enterovirus group A | 1 | Escalated | Improved | Nebulization of interferon |
| 1034603 | Bronchopneumonia | Mycoplasma pneumoniae | 1 | Human metapneumovirus; Rhinovirus type C; Haemophilus influenzae | 1 | Escalated | Improved | Prolong the duration of treatment |
| 1034482 | Bronchopneumonia | Mycoplasma pneumoniae | 1 | Human parainfluenza virus type 3 | 1 | De-escalated | Improved | Discontinue medication |
| 1034252 | Bronchopneumonia | Mycoplasma pneumoniae | 1 | Rhinovirus type A | 1 | Unchanged | Improved | Unchanged |
| 1033484 | Bronchopneumonia | Mycoplasma pneumoniae | 1 | Mycoplasma pneumoniae | 1 | Escalated | Improved | Prolong the duration of treatment |
| 1033325 | Bronchopneumonia | Mycoplasma pneumoniae | 1 | Human parainfluenza virus type 3 | 1 | Escalated | Improved | Prolong the duration of treatment |
| 1033208 | Bronchopneumonia | Mycoplasma pneumoniae | 0 | NA | 1 | De-escalated | Improved | Discontinue medication |
| 1032696 | Bronchopneumonia | Mycoplasma pneumoniae | 1 | Staphylococcus aureus; Haemophilus influenzae | 1 | Escalated | Improved | Medication added |
| 1032355 | Bronchopneumonia | Mycoplasma pneumoniae | 1 | Human metapneumovirus | 1 | Escalated | Improved | Medication added |
| 1030557 | Bronchopneumonia | Mycoplasma pneumoniae | 1 | Rhinovirus type A | 1 | De-escalated | Improved | Discontinue medication |
| 1030428 | Bronchopneumonia | Mycoplasma pneumoniae | 1 | Human metapneumovirus; Bordetella pertussis; Streptococcus pneumoniae | 1 | Escalated | Improved | Prolong the duration of treatment |
| 1029909 | Bronchopneumonia | Mycoplasma pneumoniae | 1 | Human respiratory syncytial virus type A | 1 | Escalated | Improved | Prolong the duration of treatment |
| 1028308 | Bronchopneumonia | Mycoplasma pneumoniae | 1 | Human respiratory syncytial virus type B | 1 | Escalated | Improved | Prolong the duration of treatment |
| 1027492 | Bronchopneumonia | Mycoplasma pneumoniae | 0 | NA | 1 | De-escalated | Improved | Discontinue medication |
| 1026295 | Bronchopneumonia | Mycoplasma pneumoniae | 1 | Human metapneumovirus | 1 | De-escalated | Improved | Discontinue medication |
| 989387 | Bronchopneumonia | Mycoplasma pneumoniae | 1 | Rhinovirus type A; Epstein-Barr virus | 1 | De-escalated | Improved | Discharge |
| 921567 | Bronchopneumonia | Mycoplasma pneumoniae | 1 | Mycoplasma pneumoniae; Herpes simplex virus type 1 | 1 | De-escalated | Improved | Discontinue medication |
| 921079 | Bronchopneumonia | Mycoplasma pneumoniae | 1 | Human adenovirus type 3; Mycoplasma pneumoniae; Streptococcus pneumoniae | 1 | Escalated | Improved | Medication added |
| 907729 | Bronchopneumonia | Mycoplasma pneumoniae | 0 | NA | 1 | De-escalated | Improved | Discontinue medication |
| 856337 | Bronchopneumonia | Mycoplasma pneumoniae | 1 | Mycoplasma pneumoniae | 1 | Escalated | Improved | Prolong the duration of treatment |
| 1045131 | Bronchiolitis | Mycoplasma pneumoniae | 1 | Human adenovirus type 3; Human metapneumovirus; Human respiratory syncytial virus type B; Influenza A virus | 1 | Escalated | Improved | Medication added |
| 1044150 | Asthematoid bronchopneumonia | Mycoplasma pneumoniae | 1 | Human metapneumovirus | 1 | De-escalated | Improved | Reduce the amount |
| 1043909 | Asthematoid bronchopneumonia | Mycoplasma pneumoniae | 1 | Influenza A virus H3N2; Herpes simplex virus type 1 | 1 | De-escalated | Improved | Discontinue medication |
| 1037092 | Asthematoid bronchopneumonia | Mycoplasma pneumoniae | 1 | Mycoplasma pneumoniae | 1 | Unchanged | Improved | Unchanged |
| 1031719 | Asthematoid bronchopneumonia | Mycoplasma pneumoniae | 1 | Human bocavirus type 1 | 1 | De-escalated | Improved | Discontinue medication |
| 1023344 | Asthematoid bronchopneumonia | Mycoplasma pneumoniae | 1 | Human respiratory syncytial virus type B | 1 | Escalated | Improved | Medication added |
| 1030297 | Acute upper respiratory tract infection | Mycoplasma pneumoniae | 1 | Streptococcus agalactiae; Human coronavirus OC43 | 0 | Escalated | Improved | Others |
| 1034582 | Acute suppurative tonsillitis | Mycoplasma pneumoniae | 1 | Rhinovirus type A; Streptococcus pneumoniae | 1 | Unchanged | Improved | Unchanged |
| 1045792 | Acute bronchitis, sinusitis | Mycoplasma pneumoniae | 0 | NA | 1 | Unchanged | Improved | Unchanged |
| 1024047 | Acute bronchitis, herpangina | Mycoplasma pneumoniae | 1 | Coxsackievirus type A10 | 0 | De-escalated | Improved | Others |
| 1043804 | Acute bronchitis, convulsions | Mycoplasma pneumoniae | 1 | Human metapneumovirus; Influenza A virus H3N2; Enterovirus group B | 0 | Escalated | Improved | Medication added |
| 1042796 | Acute bronchitis | Mycoplasma pneumoniae | 1 | Human adenovirus type 3; Mycoplasma pneumoniae; Bordetella pertussis | 0 | Escalated | Improved | Medications changed |
| 1042111 | Acute bronchitis | Mycoplasma pneumoniae | 1 | Human parainfluenza virus type 4 | 1 | Escalated | Improved | Medication added |
| 1032893 | Acute bronchitis | Mycoplasma pneumoniae | 1 | Human parainfluenza virus type 1 | 0 | De-escalated | Improved | Discontinue medication |
| 1030986 | Acute bronchitis | Mycoplasma pneumoniae | 1 | Human parainfluenza virus type 3; Haemophilus influenzae | 1 | Escalated | Improved | Prolong the duration of treatment |
| 1030740 | Acute bronchitis | Mycoplasma pneumoniae | 1 | Human respiratory syncytial virus type A; Rhinovirus type A; Bordetella pertussis | 1 | Unchanged | Improved | Unchanged |
| 1026556 | Acute bronchitis | Mycoplasma pneumoniae | 0 | NA | 0 | Unchanged | Improved | Unchanged |
| 1024512 | Acute bronchitis | Mycoplasma pneumoniae | 1 | Human parainfluenza virus type 3 | 1 | Escalated | Improved | Nebulization of interferon |
| 988128 | Acute bronchitis | Mycoplasma pneumoniae | 1 | Human bocavirus type 1; Streptococcus pneumoniae | 0 | De-escalated | Improved | Discontinue medication |
| 1030931 | Acute asthmatic bronchopneumonia | Mycoplasma pneumoniae | 1 | Human respiratory syncytial virus type A; Cytomegalovirus | 0 | Escalated | Improved | Prolong the duration of treatment |
| 1042184 | Acute asthmatic bronchitis | Mycoplasma pneumoniae | 1 | Influenza A virus H3N2 | 1 | Escalated | Improved | Medication added |
| 1023832 | Acute asthmatic bronchitis | Mycoplasma pneumoniae | 1 | Rhinovirus type C | 1 | De-escalated | Improved | Discontinue medication |
| 1030303 | Pneumonia-mycoplasma pneumoniae infection | Mycobacterium tuberculosis | 1 | Human metapneumovirus; Haemophilus influenzae | 1 | Escalated | Improved | Medication added |
| 1033067 | Pneumonia | Mycobacterium tuberculosis | 1 | Human metapneumovirus; Human parainfluenza virus type 1; Coxsackievirus type A10 | 1 | Escalated | Improved | Prolong the duration of treatment |
| 1030964 | Pneumonia | Mycobacterium tuberculosis | 1 | Human parainfluenza virus type 1 | 0 | De-escalated | Improved | Discontinue medication |
| 1036325 | Bronchopneumonia | Mycobacterium tuberculosis | 1 | Human parainfluenza virus type 1 | 1 | De-escalated | Improved | Discontinue medication |
| 1034141 | Bronchopneumonia | Mycobacterium tuberculosis | 1 | Human parainfluenza virus type 3; Human bocavirus type 1; Moraxella catarrhalis | 1 | Escalated | Improved | Prolong the duration of treatment |
| 912993 | Pneumonia | Haemophilus influenzae | 1 | Haemophilus influenzae | 1 | Escalated | Improved | Medications changed |
| 1044516 | Severe pneumonia | Epstein-Barr virus; Mycoplasma pneumoniae | 1 | Mycoplasma pneumoniae; Rhinovirus type B | 1 | Escalated | Improved | Others |
| 1042836 | Severe pneumonia | Epstein-Barr virus; Mycoplasma pneumoniae | 1 | Mycoplasma pneumoniae | 1 | Unchanged | Improved | Unchanged |
| 1042027 | Severe pneumonia | Epstein-Barr virus; Mycoplasma pneumoniae | 1 | Mycoplasma pneumoniae | 1 | Escalated | Aggravated | Medications changed |
| 1045358 | Pneumonia | Epstein-Barr virus; Mycoplasma pneumoniae | 1 | Mycoplasma pneumoniae | 1 | Escalated | Improved | Medication added |
| 1044120 | Pneumonia | Epstein-Barr virus; Mycoplasma pneumoniae | 1 | Mycoplasma pneumoniae; Epstein-Barr virus | 1 | De-escalated | Improved | Reduce the amount |
| 1043802 | Pneumonia | Epstein-Barr virus; Mycoplasma pneumoniae | 1 | Mycoplasma pneumoniae | 1 | De-escalated | Improved | Discontinue medication |
| 1043687 | Pneumonia | Epstein-Barr virus; Mycoplasma pneumoniae | 1 | Human adenovirus type 3; Influenza A virus; Streptococcus pyogenes | 1 | De-escalated | Improved | Discontinue medication |
| 1043289 | Pneumonia | Epstein-Barr virus; Mycoplasma pneumoniae | 1 | Mycoplasma pneumoniae | 1 | Escalated | Improved | Medication added |
| 1043198 | Pneumonia | Epstein-Barr virus; Mycoplasma pneumoniae | 1 | Mycoplasma pneumoniae | 1 | De-escalated | Improved | Discontinue medication |
| 1042811 | Pneumonia | Epstein-Barr virus; Mycoplasma pneumoniae | 1 | Human adenovirus type 3; Mycoplasma pneumoniae | 1 | De-escalated | Improved | Reduce the amount |
| 1042685 | Pneumonia | Epstein-Barr virus; Mycoplasma pneumoniae | 1 | Epstein-Barr virus | 1 | Unchanged | Improved | Unchanged |
| 1032707 | Pneumonia | Epstein-Barr virus; Mycoplasma pneumoniae | 1 | Mycoplasma pneumoniae | 1 | De-escalated | Improved | Discontinue medication |
| 1037841 | Bronchopneumonia | Epstein-Barr virus; Mycoplasma pneumoniae | 1 | Human bocavirus type 1 | 1 | Unchanged | Improved | Unchanged |
| 1036598 | Bronchopneumonia | Epstein-Barr virus; Mycoplasma pneumoniae | 1 | Mycoplasma pneumoniae | 1 | Escalated | Improved | Prolong the duration of treatment |
| 1034979 | Bronchopneumonia | Epstein-Barr virus; Mycoplasma pneumoniae | 1 | Human metapneumovirus; Human respiratory syncytial virus type A; Human parainfluenza virus type 3; Rhinovirus type A; Moraxella catarrhalis | 1 | Escalated | Improved | Medication added |
| 1032076 | Bronchopneumonia | Epstein-Barr virus; Mycoplasma pneumoniae | 1 | Human bocavirus type 1; Streptococcus pneumoniae | 1 | De-escalated | Improved | Discontinue medication |
| 1027667 | Acute upper respiratory tract infection, systemic inflammatory response syndrome | Epstein-Barr virus; Mycoplasma pneumoniae | 1 | Streptococcus pneumoniae | 1 | De-escalated | Improved | Discharge |
| 1036108 | Acute bronchitis | Epstein-Barr virus; Mycoplasma pneumoniae | 1 | Epstein-Barr virus | 1 | Escalated | Improved | Others |
| 1045498 | Severe pneumonia, purulent tonsillitis | Epstein-Barr virus | 1 | Human adenovirus type 3 | 1 | Escalated | Improved | Medication added |
| 1044616 | Severe pneumonia, abnormal liver function | Epstein-Barr virus | 1 | Human adenovirus type 3; Mycoplasma pneumoniae | 1 | Escalated | Improved | Medications changed |
| 1044944 | Severe pneumonia liver function damage | Epstein-Barr virus | 1 | Influenza A virus; Mycoplasma pneumoniae | 1 | De-escalated | Improved | Reduce the amount |
| 1042825 | Severe pneumonia | Epstein-Barr virus | 1 | Mycoplasma pneumoniae; Rhinovirus type A; Bordetella pertussis; Epstein-Barr virus | 1 | Escalated | Improved | Medications changed |
| 1042812 | Severe pneumonia | Epstein-Barr virus | 1 | Influenza A virus H3N2; Mycoplasma pneumoniae | 1 | Escalated | Improved | Medication added |
| 1040155 | Severe pneumonia | Epstein-Barr virus | 1 | Mycoplasma pneumoniae | 1 | Unchanged | Aggravated | Unchanged |
| 1034116 | Severe pneumonia | Epstein-Barr virus | 1 | Human parainfluenza virus type 4; Human parainfluenza virus type 3; Coxsackievirus type A10; Streptococcus pneumoniae | 1 | De-escalated | Improved | Discontinue medication |
| 879257 | Severe pneumonia | Epstein-Barr virus | 1 | Mycoplasma pneumoniae | 1 | Escalated | Improved | Prolong the duration of treatment |
| 1044865 | Pneumonia-mycoplasma pneumoniae infection | Epstein-Barr virus | 1 | Mycoplasma pneumoniae; Rhinovirus type A | 1 | Unchanged | Improved | Unchanged |
| 1044817 | Pneumonia-mycoplasma pneumoniae infection | Epstein-Barr virus | 0 | NA | 1 | De-escalated | Improved | Reduce the amount |
| 1043312 | Pneumonia-mycoplasma pneumoniae infection | Epstein-Barr virus | 1 | Mycoplasma pneumoniae | 1 | De-escalated | Improved | Discontinue medication |
| 1042177 | Pneumonia-mycoplasma pneumoniae infection | Epstein-Barr virus | 1 | Mycoplasma pneumoniae; Rhinovirus type A; Bordetella pertussis | 1 | Escalated | Improved | Medication added |
| 1040505 | Pneumonia-mycoplasma pneumoniae infection | Epstein-Barr virus | 1 | Mycoplasma pneumoniae | 1 | Unchanged | Improved | Unchanged |
| 1038950 | Pneumonia-mycoplasma pneumoniae infection | Epstein-Barr virus | 1 | Human coronavirus 229E; Mycoplasma pneumoniae | 1 | Escalated | Improved | Prolong the duration of treatment |
| 1037657 | Pneumonia-mycoplasma pneumoniae infection | Epstein-Barr virus | 0 | NA | 1 | Unchanged | Improved | Unchanged |
| 1034885 | Pneumonia-mycoplasma pneumoniae infection | Epstein-Barr virus | 1 | Human parainfluenza virus type 3 | 1 | Unchanged | Improved | Unchanged |
| 1029334 | Pneumonia-mycoplasma pneumoniae infection | Epstein-Barr virus | 1 | Mycoplasma pneumoniae | 1 | Escalated | Improved | Medication added |
| 1045280 | Pneumonia, suppurative tonsillitis | Epstein-Barr virus | 1 | Human adenovirus type 3 | 1 | Unchanged | Improved | Unchanged |
| 1044438 | Pneumonia, purulent tonsillitis, abnormal liver function | Epstein-Barr virus | 1 | Human adenovirus type 3; Mycoplasma pneumoniae | 1 | De-escalated | Improved | Reduce the amount |
| 1038894 | Pneumonia, laryngitis | Epstein-Barr virus | 1 | Human respiratory syncytial virus type B; Human parainfluenza virus type 1; Coxsackievirus type A6 | 1 | Escalated | Improved | Medication added |
| 1045811 | Pneumonia, EBV infection | Epstein-Barr virus | 1 | Mycoplasma pneumoniae | 1 | Escalated | Improved | Medication added |
| 1045694 | Pneumonia | Epstein-Barr virus | 1 | Mycoplasma pneumoniae | 1 | Escalated | Improved | Medication added |
| 1044959 | Pneumonia | Epstein-Barr virus | 1 | Influenza A virus | 1 | De-escalated | Improved | Others |
| 1044532 | Pneumonia | Epstein-Barr virus | 1 | Mycoplasma pneumoniae | 1 | Escalated | Improved | Prolong the duration of treatment |
| 1044519 | Pneumonia | Epstein-Barr virus | 1 | Influenza A virus H3N2; Mycoplasma pneumoniae | 1 | Unchanged | Improved | Unchanged |
| 1043992 | Pneumonia | Epstein-Barr virus | 1 | Influenza A virus H3N2; Streptococcus pneumoniae | 1 | Unchanged | Improved | Unchanged |
| 1043711 | Pneumonia | Epstein-Barr virus | 1 | Mycoplasma pneumoniae; Rhinovirus type C | 1 | De-escalated | Improved | Discontinue medication |
| 1043702 | Pneumonia | Epstein-Barr virus | 1 | Mycoplasma pneumoniae | 1 | De-escalated | Improved | Reduce the amount |
| 1043544 | Pneumonia | Epstein-Barr virus | 1 | Human adenovirus type 3; Rhinovirus type C; Haemophilus influenzae | 1 | Escalated | Improved | Medication added |
| 1043396 | Pneumonia | Epstein-Barr virus | 1 | Mycoplasma pneumoniae; Epstein-Barr virus | 1 | Unchanged | Improved | Unchanged |
| 1042919 | Pneumonia | Epstein-Barr virus | 1 | Mycoplasma pneumoniae | 1 | De-escalated | Improved | Discontinue medication |
| 1042802 | Pneumonia | Epstein-Barr virus | 1 | Mycoplasma pneumoniae | 1 | Unchanged | Improved | Unchanged |
| 1041679 | Pneumonia | Epstein-Barr virus | 1 | Influenza A virus H3N2; Mycoplasma pneumoniae; Rhinovirus type B | 1 | Unchanged | Improved | Unchanged |
| 1036617 | Pneumonia | Epstein-Barr virus | 1 | Human parainfluenza virus type 3; Streptococcus pyogenes; Epstein-Barr virus; Haemophilus influenzae | 1 | Escalated | Improved | Medications changed |
| 1035165 | Pneumonia | Epstein-Barr virus | 1 | Mycoplasma pneumoniae | 1 | Unchanged | Improved | Unchanged |
| 1035076 | Pneumonia | Epstein-Barr virus | 1 | Mycoplasma pneumoniae | 1 | Escalated | Improved | Prolong the duration of treatment |
| 1032339 | Pneumonia | Epstein-Barr virus | 0 | NA | 1 | De-escalated | Improved | Discontinue medication |
| 1031908 | Pneumonia | Epstein-Barr virus | 0 | NA | 1 | Unchanged | Improved | Unchanged |
| 1031666 | Pneumonia | Epstein-Barr virus | 1 | Human parainfluenza virus type 4; Epstein-Barr virus; Staphylococcus aureus | 1 | Escalated | Improved | Prolong the duration of treatment |
| 1031157 | Pneumonia | Epstein-Barr virus | 1 | Human parainfluenza virus type 1; Epstein-Barr virus; Moraxella catarrhalis | 1 | Escalated | Improved | Medication added |
| 1019050 | Pneumonia | Epstein-Barr virus | 1 | Rhinovirus type C; Streptococcus pneumoniae | 1 | De-escalated | Improved | Discontinue medication |
| 1031463 | Herpangina, acute suppurative tonsillitis | Epstein-Barr virus | 1 | Coxsackievirus type A10 | 0 | Escalated | Improved | Prolong the duration of treatment |
| 1039960 | Bronchopneumonia | Epstein-Barr virus | 1 | Human adenovirus type 3; Mycoplasma pneumoniae; Haemophilus influenzae | 1 | Unchanged | Improved | Unchanged |
| 1039605 | Bronchopneumonia | Epstein-Barr virus | 1 | Haemophilus influenzae | 1 | Escalated | Improved | Prolong the duration of treatment |
| 1038540 | Bronchopneumonia | Epstein-Barr virus | 1 | Epstein-Barr virus | 1 | Escalated | Improved | Others |
| 1035087 | Bronchopneumonia | Epstein-Barr virus | 1 | Human metapneumovirus; Streptococcus pneumoniae | 1 | Unchanged | Improved | Unchanged |
| 1034771 | Bronchopneumonia | Epstein-Barr virus | 1 | Human metapneumovirus; Epstein-Barr virus; Moraxella catarrhalis; Streptococcus pneumoniae | 1 | Escalated | Improved | Medication added |
| 1034125 | Bronchopneumonia | Epstein-Barr virus | 1 | Human metapneumovirus | 1 | De-escalated | Improved | Discontinue medication |
| 1033742 | Bronchopneumonia | Epstein-Barr virus | 1 | Human parainfluenza virus type 4; Epstein-Barr virus; Haemophilus influenzae | 1 | Escalated | Improved | Others |
| 1033117 | Bronchopneumonia | Epstein-Barr virus | 1 | Epstein-Barr virus | 1 | Escalated | Improved | Others |
| 1032981 | Bronchopneumonia | Epstein-Barr virus | 1 | Human bocavirus type 1; Epstein-Barr virus | 1 | Escalated | Improved | Prolong the duration of treatment |
| 1032504 | Bronchopneumonia | Epstein-Barr virus | 1 | Human bocavirus type 1; Epstein-Barr virus | 1 | Escalated | Improved | Others |
| 1032264 | Bronchopneumonia | Epstein-Barr virus | 1 | Human metapneumovirus; Haemophilus influenzae | 1 | De-escalated | Improved | Discontinue medication |
| 1031168 | Bronchopneumonia | Epstein-Barr virus | 1 | Human bocavirus type 1 | 1 | De-escalated | Improved | Discontinue medication |
| 1028825 | Bronchopneumonia | Epstein-Barr virus | 1 | Human respiratory syncytial virus type A | 1 | De-escalated | Improved | Discontinue medication |
| 1028419 | Bronchopneumonia | Epstein-Barr virus | 1 | Human respiratory syncytial virus type A; Human bocavirus type 1; Rhinovirus type A; Epstein-Barr virus | 1 | Escalated | Improved | Medication added |
| 1025558 | Bronchopneumonia | Epstein-Barr virus | 1 | Human respiratory syncytial virus type A | 1 | De-escalated | Improved | Discontinue medication |
| 1045181 | Bronchitis, suppurative tonsillitis | Epstein-Barr virus | 1 | Human adenovirus type 3; Streptococcus pneumoniae | 1 | Unchanged | Improved | Unchanged |
| 1018783 | Acute upper respiratory tract infection, systemic inflammatory response syndrome, herpangina | Epstein-Barr virus | 1 | Coxsackievirus type A10; Epstein-Barr virus | 0 | De-escalated | Improved | Discontinue medication |
| 1035482 | Acute upper respiratory tract infection | Epstein-Barr virus | 0 | NA | 0 | Unchanged | Improved | Unchanged |
| 1033265 | Acute upper respiratory tract infection | Epstein-Barr virus | 1 | Human adenovirus type 1; Rhinovirus type A; Haemophilus influenzae | 0 | Escalated | Improved | Others |
| 1023416 | Acute upper respiratory tract infection | Epstein-Barr virus | 0 | NA | 0 | De-escalated | Improved | Others |
| 1037424 | Acute suppurative tonsillitis, herpes simplex virus infection | Epstein-Barr virus | 1 | Herpes simplex virus type 1 | 1 | Escalated | Improved | Nebulization of interferon |
| 1029808 | Acute bronchitis, systemic inflammatory response syndrome | Epstein-Barr virus | 1 | Human adenovirus type 1; Rhinovirus type A; Haemophilus influenzae | 0 | Escalated | Improved | Prolong the duration of treatment |
| 1025251 | Acute bronchitis, acute tonsillitis, infectious mononucleosis | Epstein-Barr virus | 1 | Rhinovirus type C; Epstein-Barr virus | 0 | Unchanged | Improved | Unchanged |
| 1034694 | Acute asthmatic bronchopneumonia | Epstein-Barr virus | 1 | Human bocavirus type 1; Epstein-Barr virus; Cytomegalovirus | 1 | Escalated | Improved | Nebulization of interferon |
| 1043157 | Neonatal pneumonia | Enterococcus faecalis | 1 | Human respiratory syncytial virus type B | 1 | Escalated | Improved | Others |
| 884098 | Bronchopneumonia, myocardial damage | Adenovirus | 1 | Human metapneumovirus | 1 | Escalated | Improved | Others |
| 1008630 | Acute upper respiratory tract infection | Adenovirus | 1 | Rhinovirus type A | 1 | De-escalated | Improved | Discharge |
|  |  |  |  | ​ |  |  |  |  |
