# Supplementary material for: Diagnostic and Clinical Value of Targeted Next‐Generation Sequencing for Pediatric Respiratory Infections in Northern China
Source: Clin Respir J. 2026 Apr 12;20(4):e70185. doi: 10.1111/crj.70185 (PMC13070711; doi:10.1111/crj.70185)
Supplement: Supplementary file 7 — Table S6: Statistic of p values across different comparison groups. [file CRJ-20-e70185-s002.docx]

| **Supplementary Table 6. Statistic of P values across differet comparison groups.** | | | |
| --- | --- | --- | --- |
| Group | Pathogen | P value | Group with highest proportions |
| Season | Human parainfluenza virus type 4 | 0.000168681 | Autumn |
|  | Coxsackievirus type A6 | 0.000771891 | Autumn |
|  | *Bordetella pertussis* | 0.001654502 | Winter |
|  | Human respiratory syncytial virus type B | 0.001691139 | Winter |
|  | Influenza C virus | 0.018315639 | Winter |
|  | Human adenovirus type 2 | 0.030197383 | Autumn |
|  | Herpes simplex virus type 1 | 4.78E-02 | Winter |
|  | Human parainfluenza virus type 1 | 1.04E-06 | Autumn |
|  | Human parainfluenza virus type 3 | 1.49E-19 | Autumn |
|  | Human coronavirus OC43 | 1.73E-05 | Summer |
|  | Unknown subtype Influenza A virus | 1.79E-08 | Winter |
|  | Rhinovirus type C | 2.53E-05 | Winter |
|  | Coxsackievirus type A10 | 2.80E-08 | Autumn |
|  | *Mycoplasma pneumoniae* | 3.60E-51 | Winter |
|  | *Haemophilus influenzae* | 3.96E-05 | Autumn |
|  | Human metapneumovirus | 5.16E-12 | Autumn |
|  | Rhinovirus type B | 5.42E-06 | Winter |
|  | Human bocavirus type 1 | 6.14E-06 | Autumn |
|  | Human respiratory syncytial virus type A | 6.20E-15 | Summer |
|  | Human adenovirus type 3 | 6.21E-24 | Winter |
|  | Rhinovirus type A | 6.82E-05 | Summer |
|  | Influenza A virus H3N2 | 7.42E-25 | Winter |
|  | Epstein-Barr virus | 7.59E-09 | Winter |
| Age | Rhinovirus type B | 0.000144772 | 4-7Y |
|  | Coxsackievirus type A10 | 0.000204573 | 1-3Y |
|  | Herpes simplex virus type 1 | 0.001071519 | >7Y |
|  | Human parainfluenza virus type 4 | 0.001435586 | 4-7Y |
|  | Human adenovirus group C | 0.001816649 | 1-3Y |
|  | Human coronavirus OC43 | 0.004955951 | <1Y |
|  | Human respiratory syncytial virus type B | 0.005034199 | <1Y |
|  | Coxsackievirus type A6 | 0.00541582 | 1-3Y |
|  | SARS-CoV-2 omicron XBB | 0.010053444 | 4-7Y |
|  | *Moraxella catarrhalis* | 0.012079903 | <1Y |
|  | Unknown subtype Influenza A virus | 0.024563297 | <1Y |
|  | *Streptococcus pyogenes* | 0.026477676 | >7Y |
|  | *Mycobacterium tuberculosis* | 0.035110116 | 4-7Y |
|  | Human bocavirus type 1 | 1.04E-12 | 1-3Y |
|  | Human respiratory syncytial virus type A | 1.34E-08 | <1Y |
|  | *Haemophilus influenzae* | 1.36E-10 | <1Y |
|  | *Bordetella pertussis* | 1.37E-12 | >7Y |
|  | Cytomegalovirus | 1.46E-05 | <1Y |
|  | Human parainfluenza virus type 3 | 1.63E-07 | <1Y |
|  | Human metapneumovirus | 1.70E-17 | 4-7Y |
|  | Influenza A virus H3N2 | 1.73E-07 | >7Y |
|  | Human parainfluenza virus type 1 | 2.54E-06 | 4-7Y |
|  | Human adenovirus type 3 | 2.88E-14 | >7Y |
|  | *Streptococcus pneumoniae* | 3.19E-17 | 4-7Y |
|  | *Mycoplasma pneumoniae* | 3.80E-45 | >7Y |
|  | Rhinovirus type A | 4.04E-07 | 1-3Y |
|  | Epstein-Barr virus | 6.81E-18 | >7Y |
|  | Rhinovirus type C | 7.26E-05 | 4-7Y |
| Sex | *Haemophilus influenzae* | 0.033006258 | M |
|  | Human coronavirus 229E | 0.045500264 | F |
